# Phylogenetic Diversity and Genotypical Complexity of H9N2 Influenza A Viruses Revealed by Genomic Sequence Analysis

**DOI:** 10.1371/journal.pone.0017212

**Published:** 2011-02-28

**Authors:** Guoying Dong, Jing Luo, Hong Zhang, Chengmin Wang, Mingxing Duan, Thomas Jude Deliberto, Dale Louis Nolte, Guangju Ji, Hongxuan He

**Affiliations:** 1 Key Laboratory of Animal Ecology and Conservation Biology, National Research Center for Wildlife Born Diseases, Institute of Zoology, Chinese Academy of Sciences, Beijing, China; 2 Center for Computational and Systems Biology, Institute of Biophysics, Chinese Academy of Sciences, Beijing, China; 3 State Key Laboratory of Biomembrane and Membrane Biotechnology, School of Life Sciences, Tsinghua University, Beijing, People's Republic of China; 4 National Wildlife Disease Program, Wildlife Services, Animal and Plant Health Inspection Service, United States Department of Agriculture, Fort Collins, Colorado, United States of America; University of Georgia, United States of America

## Abstract

H9N2 influenza A viruses have become established worldwide in terrestrial poultry and wild birds, and are occasionally transmitted to mammals including humans and pigs. To comprehensively elucidate the genetic and evolutionary characteristics of H9N2 influenza viruses, we performed a large-scale sequence analysis of 571 viral genomes from the NCBI Influenza Virus Resource Database, representing the spectrum of H9N2 influenza viruses isolated from 1966 to 2009. Our study provides a panoramic framework for better understanding the genesis and evolution of H9N2 influenza viruses, and for describing the history of H9N2 viruses circulating in diverse hosts. Panorama phylogenetic analysis of the eight viral gene segments revealed the complexity and diversity of H9N2 influenza viruses. The 571 H9N2 viral genomes were classified into 74 separate lineages, which had marked host and geographical differences in phylogeny. Panorama genotypical analysis also revealed that H9N2 viruses include at least 98 genotypes, which were further divided according to their HA lineages into seven series (A–G). Phylogenetic analysis of the internal genes showed that H9N2 viruses are closely related to H3, H4, H5, H7, H10, and H14 subtype influenza viruses. Our results indicate that H9N2 viruses have undergone extensive reassortments to generate multiple reassortants and genotypes, suggesting that the continued circulation of multiple genotypical H9N2 viruses throughout the world in diverse hosts has the potential to cause future influenza outbreaks in poultry and epidemics in humans. We propose a nomenclature system for identifying and unifying all lineages and genotypes of H9N2 influenza viruses in order to facilitate international communication on the evolution, ecology and epidemiology of H9N2 influenza viruses.

## Introduction

Sixteen HA and nine NA subtype influenza A viruses have been detected in poultry and wild birds around the world [1]. Most of these viruses have become established in domestic poultry resulting in mild or severe disease and pose a threat of zoonotic infection [2]–[5]. H9 subtype viruses generally exist as low pathogenicity influenza viruses causing mild to moderate disease. However, they have been associated with severe morbidity and mortality in poultry as a result of co-infection with other pathogens [6], [7]. The first H9N2 influenza virus was isolated from turkeys in Wisconsin in 1966 [8]. Since then, H9N2 viruses have been detected mainly in shorebirds and wild ducks in North America [6]. Throughout Asia, the Middle East, Europe and Africa, H9N2 viruses have been isolated in multiple avian species, particularly in land-based poultry [9]–[13]. H9N2 viruses have also been found in pigs [14], which have been proposed to be ‘mixing vessels’ for the genesis of potentially pandemic influenza reassortments [15], [16]. More importantly, some H9N2 viruses have acquired the typical receptor specificity of human influenza viruses, and have been transmitted directly to humans causing mild respiratory disease [4], [5]. In addition, recent studies have shown that H9N2 viruses may have contributed to the genetic and geographic diversity of H5N1 viruses [5], [17], such as the 1997 Hong Kong H5N1 influenza viruses which had internal genes probably derived from the isolate A/Quail/HongKong/G1/97(H9N2) [5], [18]. These findings suggest that continuous circulation of H9N2 influenza viruses among different hosts has the potential to cause outbreaks in poultry and epidemics in humans.

During the last two decades, antigenic and genetic analyses of H9N2 isolates showed their gradual and complex evolution [19], [20]. Several distinct sublineages from the Eurasian lineage have become established in domestic birds [20], [21]. Phylogenetic and genotypical analysis revealed that H9N2 viruses have undergone extensive reassortments to generate multiple novel genotypes with gene segments from different lineages [22], [23]. Notably, previous studies of the phylogenetic diversity of H9N2 viruses have focused on limited periods, regions, hosts or viral lineages, and detailed characteristics of H9N2 viruses have not been well defined [24]. Moreover, H9N2 viruses have evolved into many different lineages and sublineages, but how many genotypes actually exist within H9N2 viruses is still unclear. Some lineages and sublineages have been recognised, such as the Ck/Bei-like lineage and G1 sublineage [25], [26]. Xu et al. classified some H9N2 isolates with different gene constellations into genotype series (A0 or B0) [3]. Current nomenclature method seems to be ambiguous, and sometimes even misleading [24]. Therefore, it is necessary to identify all lineages, sublineages, and in particular, genotypes within H9N2 viruses, and ultimately to unify their nomenclature for better understanding evolutionary characteristics of H9N2 influenza viruses.

In this study, we performed a large-scale genomic sequence analysis of 571 H9N2 influenza viruses isolated from 1966 to 2009. Our analysis identified 74 lineages and 98 genotypes that could be further divided into seven series. Our results suggest that phylogenetic diversity and genotypical complexity exist in H9N2 influenza viruses. We provide a framework for obtaining a panoramic understanding of H9N2 viral evolution and propose a precise nomenclature system for unifying all lineages and genotypes of H9N2 influenza viruses in order to improve our ability to predict the direction of evolution by monitoring changes in the viral genome.

## Results

### Host and regional distribution of H9N2 influenza A genomes

A total of 571 complete genomic sequences of H9N2 influenza viruses isolated from 1966 to 2009 was used in this study. These H9N2 viruses, including 552 avian, 14 swine, and 5 human isolates, were obtained from multiple countries in Asia, the Middle East, Europe, Africa, and North America. The numbers of viruses distributed in different hosts and regions are shown in Table 1. Results suggest that the only wide distribution of H9N2 influenza viruses over multiple hosts occurred in China.

**Table 1:** Distribution of H9N2 influenza viruses in different hosts and regions from 1966 to 2009.

Host	Numbers of H9N2 influenza A viruses in different hosts and regions													
	China	Japan	Korea	Pakistan	Israel	Iran	Dubai	Saudi Arabia	Ireland	Germany	The Netherlands	South Africa	Canada	The United States
Chicken	206	11	6	14	40	1	4	1		1				
Duck	41	2	1							1				
Goose														1
Quail	77						3							
Partridge^a^	32													
Chukkar^a^	13													
Pheasant^a^	24								1					
Silky chicken^a^	15		1											
Guinea fowl^a^	8													
Turkey					15									6
Avian					3									
Swine	12		2											
Human	4		1											
Pigeon	3													
Parakeet		2												
Wild Duck	2													
Mallard													3	
Laughing gull														1
Shorebird														2
Sanderling														1
Ostrich					1							1		
Eurasian wigeon											1			
Gadwall											1			
Bewick swan											1			
Bird	5													

^*a*^represents other minor poultry.

### Phylogenetic diversity of H9N2 influenza viruses

Eight viral gene segments of 571 H9N2 viruses were aligned and analyzed phylogenetically. The panorama view of the resulting phylogenetic trees (Figure S1) illustrates six main characteristics:

Firstly, each of the eight gene segments from H9N2 influenza genomes could be divided into distinct lineages, and a total of 74 lineages were recognized in this study. These distinct lineages are likely the result of long-term ecological and geographical separations of the hosts. Their evolutionary relationships are shown in Figure S1 and their distribution over eight gene segments is summarized in Table 2. The 17 viral isolates listed in Table 2 were chosen to represent distinct lineages whose characteristics are shown in Figure S2. These findings emphasize the complexity and diversity of H9N2 influenza viruses.

**Table 2:** Lineage distribution of the eight gene segments in the 571 H9N2 influenza A genomes from 1966 to 2009.

Lineage	Representative virus	Distribution of the eight gene segments							
		PB2	PB1	PA	HA	NP	NA	M	NS
HK/G1/97	A/quail/Hongkong/G1/97(H9N2)	+	+	+	+	+	+	+	+
BJ/1/94	A/chicken/Beijing/1/94(H9N2)	+	+	+	+	+	+	+	+
HK/289/78	A/duck/Hongkong/289/78(H9N2)	+	+	+	+	+	+	+	+
HK/AF157/92	A/quail/Hongkong/AF157/92(H9N2)	−	+	−	+	+	+	+	+
HK/G9/97	A/chicken/Hongkong/G9/97(H9N2)	−	−	−	−	−	+	−	−
SH/F/98	A/chicken/Shanghai/F/98(H9N2)	+	+	+	−	+	−	−	−
ST/163/04	A/duck/Shantou/163/2004(H9N2)	+	−	−	−	−	−	−	−
ST/5663/01	A/quail/Shantou/5663/2001(H9N2)	−	−	+	−	−	−	−	−
HK/Y439/97	A/duck/Hongkong/Y439/97(H9N2)	−	−	+	−	+	+	−	+
KR/96323/96	A/chicken/Korea/38349-p96323/96(H9N2)	+	+	+	+	+	+	+	+
HoK/49/98	A/duck/Hokkaido/49/98(H9N2)	−	+	+	−	−	−	−	−
IL/90658/00	A/chicken/Israel/90658/2000(H9N2)	−	+	−	−	−	−	−	+
DE/113/95	A/Duck/Germany/113/95(H9N2)	+	+	+	+	+	+	+	−
PK/UDL-01/05	A/chicken/Pakistan/UDL-01/2005(H9N2)	+	−	−	−	−	−	−	−
CA/189/66	A/turkey/California/189/66(H9N2)	−	−	−	−	−	−	−	+
WI/1/66	A/turkey/Wisconsin/1/1966(H9N2)	+	+	+	+	+	+	+	+
H5N1	A/duck/Guangxi/xa/2001(H5N1)	+	+	+	−	−	−	+	−

+, represents distributed lineage on eight gene segments. −, represents absent lineage on eight gene segments.

Secondly, the phylogenetic trees constructed for each gene segment differed in topology and in the way they classified lineages (Figure S1, Table 2). The average genetic distance between distinct lineages was greatest for the NS genes (153.0%, ranging from 40.6% to 420.5%), and smallest for the M genes (68.5%, ranging from 38.2% to 90.6%). The HK/G1/97, BJ/1/94, HK/289/78, KR/96323/96, and WI/1/66 lineages were present in all eight gene segments. In contrast, the ST/5663/01, HK/G9/97, CA/189/66, ST/163/04, and PK/UDL-01/05 lineages were only present in single gene segments. These results further highlight the phylogenetic diversity of H9N2 influenza viruses.

Thirdly, these H9N2 virus lineages had marked host and geographical differences in phylogeny (Figure S1A–H). In our panorama phylogenetic analysis, the BJ/1/94 lineage clustered most H9N2 isolates from chickens, pigs, and other minor poultry (e.g. partridge, chukkar, Guinea fowl, pheasant, and silky chicken) [25]. These isolates were mainly from China and Japan, and showed gradual evolution over the last several years. Most of them had 9-nt deletions at positions 206 to 214 in the NA stalk. Notably, the BJ/1/94 lineage has become the dominant chicken H9N2 virus lineage in China since the late 1990s [21]. The HK/G1/97 lineage included all Israeli, Pakistani, and Dubai H9N2 isolates from chickens and turkeys, and also clustered many China H9N2 isolates from quail. HK/G1/97-like viruses also had nucleotide deletions at positions 131 to 136, 157 to 168, and 167 to 184 in the NA stalk region. The KR/96323/96 lineage, possibly derived from migratory ducks [27], contained all analyzed H9N2 isolates detected in Korea from diverse hosts (chicken, duck, pig, and human) from 1996 to 2004. Similarly, the IL/90658/00 lineage also clustered all isolates from Israel (Figure S1D). These results indicate that H9N2 virus lineages have certain distinct geographical features in phylogeny.

In contrast to the BJ/1/94 lineage, the DE/113/95, HK/289/78, Hok/49/98, and HK/Y439/97 lineages were shown here to originate from aquatic birds (Figure S1A–H). The DE/113/95 lineage represent duck isolates from China, Germany, and Japan from 1995 onwards, while the HK/289/78 lineage clustered 1978–1979 duck isolates from Hong Kong and 1988–1991 mallard isolates from Canada. These two lineage viruses had deletions in the NA stalks at positions 187 to 261 and 193 to 255, respectively. Moreover, isolates A/duck/Germany/113/1995 and A/duck/Hongkong/289/78 showed a strikingly close relationship in the NS1 coding region, exhibiting 97.1% nucleotide identity (Figure S1H). The HK/Y439/97 lineage originated from A/duck/Hongkong/Y439/1997(H9N2) which had DE/113/95-like HA and M genes (Figure S1A and G), and clustered H9N2 duck isolates from Hokkaido (Figure S1F). Of special note, in the PB1 phylogenetic tree, H9N2 Shantou isolates derived mainly from quails and humans clustered into the HK/G1/97 lineage, while Shantou isolates mainly from ducks clustered into another lineage designated as Hok/49/98 (Figure S1D). These findings suggest that H9N2 virus lineages have certain distinct host features in phylogeny.

Fourthly, novel reassortant H9N2 viruses can be generated by exchanging gene segments from different lineages. For example, the WI/1/66 lineage clustered Canadian and United States isolates mainly from turkeys, mallard ducks, and shorebirds, and formed an independent North American cluster (Figure S1A–H). Notably, the H9N2 isolate A/chicken/Heilongjiang/35/00, containing BJ/1/94-like and HK/G1/97-like gene segments, belonged to the North American WI/1/66 lineage in the HA and NP phylogenetic trees (Figure S1A and F), sharing 99.9% homology with A/turkey/Wisconsin/1/1966, which was first isolated in North America in 1966 [8]. Similarly, another H9N2 isolate, A/swine/Korea/S190/2004, derived from the KR/96323/96 lineage, also grouped with the WI/1/66 lineage in the PB1, PA, NP, and M phylogenetic trees (Figure S1D–G). These results show that it is possible for H9N2 influenza viruses from different hosts and regions to generate novel reassortant viruses carrying evolutionarily distant gene segments by gene exchange.

Fifthly, some H9N2 virus lineages are represented by single virus or only distributed in single gene segments (Figure S1A–H). For example, the HK/AF157/92 lineage was represented by the single virus A/quail/Hongkong/AF157/92, while the following five lineages were only distributed in single gene segments: in the NA phylogenetic tree, the HK/G9/97 lineage clustered some H9N2 isolates from poultry, wild bird, pig, and human. These isolates had full-length NA genes and were mainly distributed in southern China (Figure S1B). In the PB2 phylogenetic tree, the PK/UDL-01/05-like viruses from chickens formed an independent lineage, while a few duck isolates from Shantou grouped into the ST/163/04 lineage (Figure S1C). In the PA phylogenetic tree, the Shantou H9N2 isolates, derived mainly from quails and other minor poultry, grouped into the ST/5663/01 lineage (Figure S1E). For the NS genes, H9N2 influenza viruses were classified into two primary clades (A and B) as previously described (Figure S1H) [28]. Clade A is represented by the CA/189/66 lineage viruses, which were mainly isolated from Japan, the Netherlands, and the United States. These results further reveal the complexity of H9N2 viral evolution.

Finally, these H9N2 viruses were closely related to H5N1, H5N3, H3N8, H4N3, H4N6, H7N7, H10N4, and H14N5 viruses in their internal gene phylogeny. To be specific, in phylogenetic trees of internal genes, HK/G1/97-like viruses (e.g. HK/1073/99 and HK/1074/99) showed a close evolutionary relationship with the 1997 Hong Kong H5N1 influenza viruses (Figure S1C–H). In phylogenetic trees of ribonucleoprotein complex genes, SH/F/98-like viruses, descended from A/chicken/Shanghai/F/1998 (H9N2) which may have been the donor of H5N1-like internal genes [3], [29], formed separate clusters containing A/duck/Shanghai/35/2002 (H5N1), implying that the SH/F/98 lineage is highly related to H5N1-like viruses (Figure S1C–F). In the PB1 phylogenetic tree, Guangxi H9N2 isolates from 2004 to 2006 formed a separate fork and clustered into the H5N1 lineage, exhibiting 93.1% to 99.2% homology to H5N1 isolates (Figure S1D). In the PB2 phylogenetic tree, ST/163/04-like viruses were closely related to the H7N7 human isolate A/Netherlands/219/2003 (Figure S1C). In the PA phylogenetic tree, the DE/113/95 lineage contained A/mallard duck/AST/266/1982(H14N5) (Figure S1E). In the NP phylogenetic tree, isolates A/mallard/Sweden/65/2005 (H4N3) and A/migratory duck/Jiangxi/6568/2004 (H4N6) grouped the HK/Y439/97 lineage, while the isolate A/northern pintail/Alaska/44202-103/2006(H3N8) clustered into the WI/1/66 lineage (Figure S1F). In M and NS phylogenetic trees, A/mallard/Sweden/4/2005(H10N4) belonged to the DE/113/95 lineage, while A/duck/Hokkaido/84/2002(H5N3) had a close relationship with WI/1/66-like viruses (Figure S1G–H). These findings indicate that H9N2 viruses are closely related to other subtype influenza viruses including the highly pathogenic H5 and H7 subtypes.

### Panorama genotypic diversity of H9N2 viruses based on gene constellations

**Figure 1:**
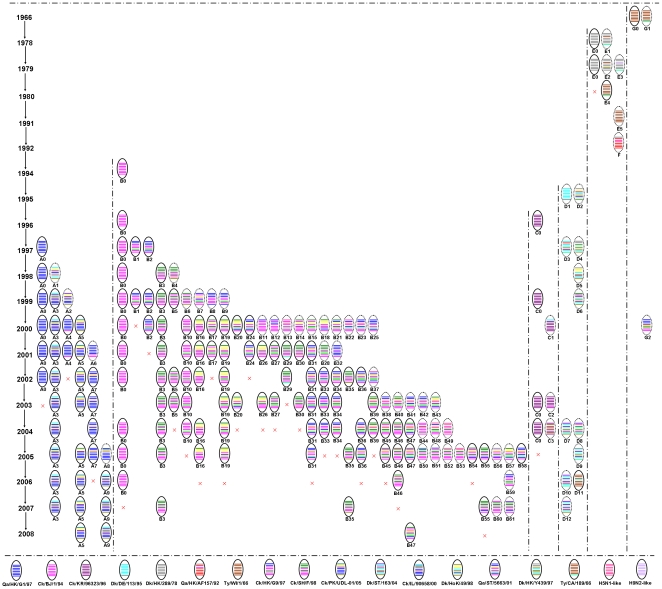
Genotypic distribution of the 571 H9N2 influenza viruses used in this study. H9N2 virus genotypes were classified into A–G series and indicated by capital letters. Eight gene segments in each of the schematic virus particles are arranged from top to bottom to represent PB2, PB1, PA, HA, NP, NA, M, and NS genes, and are indicated in same color with representative viruses for each lineage. Abbreviations: BJ, Beijing; CA, California; Ck, chicken; DE, Germany; Dk, duck; HK, Hong Kong; HoK, Hokkaido; IL, Israel; KR, Korea; PK, Pakistan; Qa, quail; SH, Shanghai; ST, Shantou; Ty, turkey; and WI, Wisconsin.

Phylogenetic analysis of the eight gene segments revealed that H9N2 viruses have undergone multiple reassortments to generate novel genotypes. With the growing accumulation of genetic information from H9N2 viruses, it is necessary to provide a systematic nomenclature for identifying viruses with different sources and gene constellations. The analyzed H9N2 viruses were divided into 98 genotypes based on genomic diversity, and their host and regional distributions are summarized in Tables 3 and 4. These genotypes can be further divided according to their HA lineages into seven series, designated as A–G. Their evolutionary relationships are shown in Figure 1 and Table S1.

**Table 3:** Host distribution of different genotypes of H9N2 influenza A viruses from 1966 to 2009.

Host	Distribution of the genotype series A–G						
	A	B	C	D	E	F	G
Chicken	A0, A1–A3, A5, A9.	B0, B1–B13, B16–B17, B19, B21–B23, B26–B31, B33–B35, B39–B40, B45–B47, B49, B52, B54–B58, B60–B61.	C0, C2.				G2
Duck		B0, B2–B3, B5, B15–B17, B19, B24–B25, B31, B34–B35, B39, B46–B47.	C0	D1, D4–D8.	E0, E1–E3.		
Goose					E4		
Quail	A0, A4–A7.	B0, B2, B14, B16–B19, B24, B31–B32, B34, B36–B38, B41, B43, B45–B47, B58.				F	
Partridge	A0	B19–B20, B24–B25, B31, B39, B45, B47, B58.					
Chukkar	A8	B31, B36, B39, B42, B45.					
Pheasant		B5, B19–B20, B31, B34, B36, B39, B45, B47–B48, B58.		D3			
Silky chicken		B0, B5, B10, B16, B19, B31, B39, B45, B47.	C0				
Guinea fowl	A0	B5, B16, B31, B42, B45.					
Turkey	A3, A9.				E4		G0, G1.
Avian	A3, A9.						
Swine		B0, B3, B10, B30, B44, B46, B50–B51, B53.	C0, C3.				
Human	A0	B2, B31.	C1				
Pigeon	A0	B0, B1.					
Parakeet	A0						
Wild Duck		B0, B31.					
Mallard					E4, E5.		
Laughing gull				D11			
Shorebird				D11			G0
Ostrich	A3			D2			
Eurasian wigeon				D9			
Gadwall				D10			
Bewick swan				D12			
Sanderling				D11			
Bird		B46, B59.					

**Table 4:** Regional distribution of different genotypes of H9N2 influenza viruses from 1966 to 2009.

Region	Distribution of the genotype series A–G						
	A	B	C	D	E	F	G
China	A0, A4, A6–A8.	B0, B1–B28, B30–B61.		D4, D7, D8.	E0, E1–E3.	F	G2
Japan	A0	B0, B3, B10, B26, B27, B29.		D5, D6.			
Korea			C0, C1–C3.				
Pakistan	A0, A5.						
Israel	A3, A9.						
Iran	A2						
Dubai	A5						
Saudi Arabia	A3						
Ireland				D3			
Germany	A1			D1			
The Netherlands				D9, D10, D12.			
South Africa				D2			
Canada					E4, E5.		
The United States				D11	E4		G0, G1.

Panorama genotypic analysis revealed that seven genotypic series of H9N2 viruses differed in their host and regional distributions (Table S1, Tables 3 and 4). The genotype A series including genotypes A0–A9 was distributed in Asia, the Middle East, and Europe. Genotype A0, representing nonreassortant HK/G1/97-like isolates mainly from quails and humans, was detected in China, Japan, and Pakistan from 1997 to 2002. Genotypes A1–A3 were found in chickens during 1998–1999 in Germany, Iran, and Saudi Arabia respectively, and genotype A3 has become the dominant H9N2 virus circulating in Israel since 2000. During 2000–2002, genotypes A4–A7 were detected in quails from China and Dubai, while genotype A5 emerged in chickens and has become the dominant H9N2 virus in Pakistan since 2005. Genotype A9 representing HK/G1/97-like reassortants circulated in chickens in Israel from 2006 onwards.

The genotype B series containing genotypes B0–B61 was only detected in China and Japan (Tables 3 and 4, Table S1). Genotype B0, representing nonreassortant BJ/1/94-like isolates, was detected in chickens mainly from China during 1994 to 2006 and also emerged in pigs from China in 1998. Genotype B3, representing SH/F/98-like viruses, has widely circulated in chickens in China since its first emergence in 1998, suggesting that this genotype has become established in chickens (Figure 1). Genotypes B5–B9 were present in chickens in southern China in 1999, while genotypes B10–B25 circulated in poultry and pigs in the same region in 2000. Notably, genotypes B10, B16, and B19 have become the dominant H9N2 viruses and have been widely prevalent in poultry from Shantou, China, since 2000 (Figure 1, Table S1). Genotypes B26–B32 were identified in China and Japan during 2001. Specially, genotype B29 was only found in Japanese chickens, while genotype B31 viruses were prevalent in other minor poultry in China. From 2002 onwards, genotypes B33 to B61 representing complicated H9N2 reassortants with gene segments from multiple lineages continued to circulate in China. Of these genotypes, B44, B50, B51, and B53 were only detected in pigs, while the remaining genotypes were recognized in diverse poultry species (Table S1).

The genotype C series comprising genotypes C0–C3 was only detected in Korea (Tables 3 and 4, Table S1). Genotype C0, representing nonreassortant KR/96323/96-like viruses, was detected in poultry and pigs from Korea from 1996 to 2004. Genotype C1 was recognized in an H9N2 human isolate A/Korea/KBNP-0028/2000. Genotype C2 emerged in Korean chickens in 2003, while genotype C3 appeared in pigs from Korea during 2004.

The genotype D series, containing genotypes D1–D12, was widely distributed throughout the world except for the Middle East (Table S1, Tables 3 and 4). Genotypes D1 and D4 were only detected in duck H9N2 isolates A/duck/Germany/113/95 and A/duck/Hong Kong/Y439/97 respectively, and each of their gene segments was of pure duck origin. Genotypes D5–D8 were also detected in ducks from China and Japan. Genotype D2 was found in ostrich isolates from South Africa in 1995, genotype D11 was identified in laughing gulls, shorebirds, and sanderlings from Delaware in 2006, and genotypes D9–D10 and D12 were detected separately in Eurasian wigeon, gadwall, and bewick swans in the Netherlands during 2005–2007. These genotypes represented multiple H9N2 reassortants from wild birds, suggesting that H9N2 viruses have become more and more diversified in these regions.

The genotype E series representing genotypes E0–E5 was found in China, Canada, and the United States (Tables 3 and 4, Table S1). Genotype E0 nonreassortant HK/289/78-like viruses, and genotype E1–E3 viruses were detected in ducks from Hong Kong during 1978–1979. Genotype E4 emerged in the United States from geese in 1980 and from turkeys in 1981, while genotype E5 appeared in mallards from Canada in 1991. The genotype F series was represented by only a single China isolate A/quail/Hongkong/AF157/92. The genotype G series, representing nonreassortant WI/1/66-like viruses (genotype G0) and two reassortants (genotypes G1–G2), was detected in turkeys from the United States in 1966 and in chickens from China in 2000 (Table S1, Tables 3 and 4).

Panorama genotypic analysis also revealed that the genotypic complexity of H9N2 viruses resulted from extensive reassortments (Figure 1, Table S1). For example, genotype A3 viruses contained gene segments derived from the HK/G1/97, DE/113/95, IL/90658/00, HK/Y439/97, and KR/96323/96 lineages. Genotype B46 viruses, the first genotype in which H5N1-like PB1 genes were present, are descended from SH/F/98-like viruses, and are likely the result of multiple reassortments between co-circulating H9N2-like and H5N1-like viruses. The genotype C3 virus appears to have evolved from the KR/96323/96-like virus by reassorting WI/1/66-like PB1, PA, NP, and M genes. Genotype D11 viruses consisted of seven WI/1/66-like and one DE/113/95-like gene segments. Genotype E4 reassortant viruses included gene segments from WI/1/66, HK/289/78 and CA/189/66 lineage. The genotype F virus reassorted HK/289/78-like and BJ/1/94-like genes. The genotype G2 virus was a quadruple reassortant containing genes derived from HK/G1/97, HK/G9/97, BJ/1/94, and WI/1/66 lineages. These findings suggest that novel genotypical H9N2 influenza viruses can be generated by multiple reassortments.

## Discussion

We have analyzed all eight gene segments of 571 publicly-available H9N2 influenza A genomes and provide a panoramic framework for better understanding the genesis and evolution of H9N2 influenza viruses. In this set of 571 H9N2 genomes, we observed multiple novel mutational and reassortant events, such as deletions in NA genes and segment exchanges in internal genes, particularly in the ribonucleoprotein complex genes. After careful analysis of these changes, we have presented the first characterization of the phylogeny and genetic diversity of H9N2 viruses worldwide.

Given the importance of classification of lineages for studies on viral epidemiology, evolution, and ecology [19], [30], we have proposed a precise nomenclature system for identifying and unifying all lineages and genotypes of H9N2 viruses. The topologies of phylogenetic trees calculated in this study with MEGA were all consistent with previous reports [19], [23], [24]. All 74 lineages identified within analyzed H9N2 viruses had high bootstrap values at nodes (>70), and were supported by the topologies of phylogenetic trees (Figure S1). These lineages had marked host and geographical differences in phylogeny, implying the existence of phylogenetic diversity in H9N2 influenza viruses.

Our phylogenetic maps provide a framework for describing the history of H9N2 viral circulation in diverse hosts, and also provide new information for further confirming the genetic and evolutionary characteristics of H9N2 viruses. Firstly, panorama phylogenetic analysis reveals that H9N2 viruses are closely related to H3, H4, H5, H7, H10, and H14 subtype influenza viruses in phylogenies of internal genes [22]. Secondly, the H9N2 geographical distribution was enlarged compared to previous studies [31], [32] to include Asia, the Middle East, Europe, Africa, and North America, and some H9N2 viruses from Asia and Europe were found to be located in the North American WI/1/66 or CA/189/66 lineages, suggesting that H9N2 viruses have become more complicated. Thirdly, analysis of virus phylogeny indicates that avian H9N2 viruses are of high diversity [3], [9], [25], and several permanent lineages have become established in land-based poultry in Asia [10], [17], [33]. By comparison, infection of pigs with H9N2 viruses was frequent, particularly in China [14], [34], implying that pigs might play an important role in the ecology and epidemiology of H9N2 influenza viruses [14]. In particular, the HK/G1/97-like H9N2 human isolates are closely related to the 1997 Hong Kong H5N1 influenza viruses which gave rise to six fatalities among 18 infected patients [5], [18], suggesting that these H9N2 viruses still have pandemic potential. Finally, our panorama phylogenetic analysis demonstrates that H9N2 influenza viruses have undergone extensive reassortments since 1997, particularly in China (Figure 1), suggesting that novel H9N2 reassortant viruses can be generated by gene exchanges.

Our panorama genotypical analysis revealed the complexity and diversity of genotypes among the analyzed H9N2 viruses. These H9N2 viruses included 98 genotypes which were classified into seven series (A–G) representing HK/G1/97, BJ/1/94, KR/96323/96, DE/113/95, HK/289/78, HK/AF157/92, and WI/1/66 lineage viruses, respectively (Figure 1). Of these viruses, the genotype A series virus was genetically stable and well adapted to quails. However, some viruses (genotypes A1–A3, A5, and A9) underwent reassortments with local H9N2 viruses and caused outbreaks in chickens in Pakistan, Israel, Dubai, Iran, Saudi Arabia, and Germany [22], [33], [35], [36]. The genotype B series virus became prevalent primarily in chickens and other minor poultry after its primary emergence in China [21], [25], and was transmitted back to ducks from 1997 onwards [23], or co-circulated with genotype A series viruses in quail since 2000 to generate multiple reassortants and genotypes (Table S1) [3]. However, genotype B series viruses were of genetically unstable and transient gene constellations (Figure 1). Genotype D and E series viruses circulated in ducks, in contrast to genotype C and G series viruses which were detected mainly in chickens and turkeys, respectively. These findings indicate that host restriction exists in H9N2 viruses.

Our panorama genotypical analysis also revealed that multiple reassortments of H9N2 viruses were capable of facilitating the development of multifarious genotypes and, ultimately, the emergence of novel genotypes in diverse hosts. For example, genotype B46 reassortant virus was first recognized in chickens in 2004 and became predominant in poultry, pigs, and birds during 2005–2006. These novel genotypical H9N2 reassortants are constantly evolving in diverse hosts, raising the risk of H9N2 influenza viruses to be introduced into humans.

In summary, our large-scale sequence analysis of 571 H9N2 viral genomes has revealed the phylogenetic diversity and genotypic complexity of H9N2 influenza viruses worldwide. Results from our study also indicate that multifarious genotypical H9N2 viruses are continuously circulated in diverse hosts throughout the world, raising concerns over their potential role in causing future influenza outbreaks in poultry and epidemics in humans. We have proposed a precise nomenclature system for identifying and unifying all lineages and genotypes of H9N2 viruses which should facilitate international communication on the evolution, ecology and epidemiology of H9N2 influenza viruses.

## Methods

### H9N2 Influenza virus sequences

All influenza virus sequences used in this study were obtained from GenBank. Viral sequences that were identical, inaccurate, unclear background, or shorter than 500 bp were removed. The 571 complete H9N2 genomic sequences, collected from multiple countries between 1966 and 2009, were selected from the NCBI Influenza Virus Resource Database (http://www.ncbi.nlm.nih.gov/genomes/FLU/FLU.html), together with some H3, H4, H5, H6, H7, H10, and H14 subtype reference sequences. GenBank accession numbers for all analyzed sequences are listed in Table S2.

### Phylogenetic analysis

Multiple nucleotide sequence alignments were performed separately on the eight viral gene segments of representative viruses using Clustal W (BioEdit version 7.0.5) (http://www.mbio.ncsu.edu/BioEdit/bioedit.html). Genetic distances among the representative sequences were calculated using the model of pairwise distance calcualtion (MEGA version 4.0.2) (http://www.megasoftware.net/). 1000 bootstrap replicates were performed and phylogenetic trees were constructed for each genomic segment by the neighbor-joining method using the MEGA 4.0.2 program.

### Classification of lineages

All lineages from the eight gene segments of representative viruses were classified according to genetic distances and topologies of phylogenetic trees. Genetic distances between distinct lineages were calculated by comparison with selected sequences from representative viruses listed in Table 2. Distribution of representative viruses over time, regions and hosts was also considered in the classification of lineages. The nomenclature system used here to represent H9N2 virus lineages includes (in order) their place of isolation, virus number, and isolation time. For example, “WI/1/66” indicates that this lineage was the first virus isolated in 1966 in Wisconsin, USA. Lineages of other subtype reference viruses were designated with the subtype name (e.g. H5N1 lineage).

### Genotypic analysis

Genotypic analysis was performed systematically for each of the eight gene segments based on the distribution of lineages in phylogenetic trees. Genes sharing over 95% homology in the same lineage were considered as one genotypic group. H9N2 viruses with different gene constellations were divided according to their HA lineages into seven series, designated as A–G. In genotype A series, nonreassortant viruses were designated as A0, while reassortant viruses were designated sequentially as A1, A2, and so on, according to when the novel genotype was first identified. By parity of reasoning, other genotype series viruses were also designated with systematic nomenclature. Genotypes of all H9N2 influenza viruses analyzed are summarized in Figure 1 and Table S1.

## Supporting Information

Figure S1 **Phylogenetic relationships for HA (A), NA (B), PB2 (C), PB1 (D), PA (E), NP (F), M (G) and NS (H) genes of the 571 analyzed H9N2 influenza viruses.** Genome sequences of 571 H9N2 viruses from 1966 to 2009 and other subtype reference viruses were selected from the NCBI Influenza Virus Resource Database. The following nucleotide fragments were used in the phylogenetic analysis: HA, 148 to 1230; NA, 20 to 1390; PB2, 1063 to 2246; PB1, 205 to 1428; PA, 796 to 2016; NP, 76 to 996; M, 80 to 947 and NS, 48 to 831. Neighbor-joining trees were constructed using MEGA and bootstrap values are shown for the key nodes. Clades labeled using different colors indicate major H9N2 virus lineages. Human viruses are colored scarlet, swine viruses in deep blue and avian viruses in black. Representative viruses for each lineage are underlined in the same color as clades, and are highlighted in italics together with human and swine viruses.
(TIF)

Figure S2 **Phylogenetic characteristics of HA (A), NA (B), PB2 (C), PB1 (D), PA (E), NP (F), M (G) and NS (H) genes of H9N2 influenza viruses.** 571 H9N2 viral genomic sequences from 1966 to 2009 were characterized. Neighbor-joining phylogenetic trees were generated with MEGA. Clades labeled using different colors indicate major H9N2 virus lineages.Virus abbreviations are listed in the legend of Figure 1.
(TIF)

Table S1 **Gene constellations of different genotypes of the 571 H9N2 influenza A viruses.**
(DOC)

Table S2 **Accession numbers of nucleic acid sequences for the 571 H9N2 influenza A and other subtype reference viruses used in this study.**
(DOC)
