# Supplementary figures and images for: Phylogenetic Diversity and Genotypical Complexity of H9N2 Influenza A Viruses Revealed by Genomic Sequence Analysis
Source: PLoS One. 2011 Feb 28;6(2):e17212. doi: 10.1371/journal.pone.0017212 (PMC3046171; doi:10.1371/journal.pone.0017212)

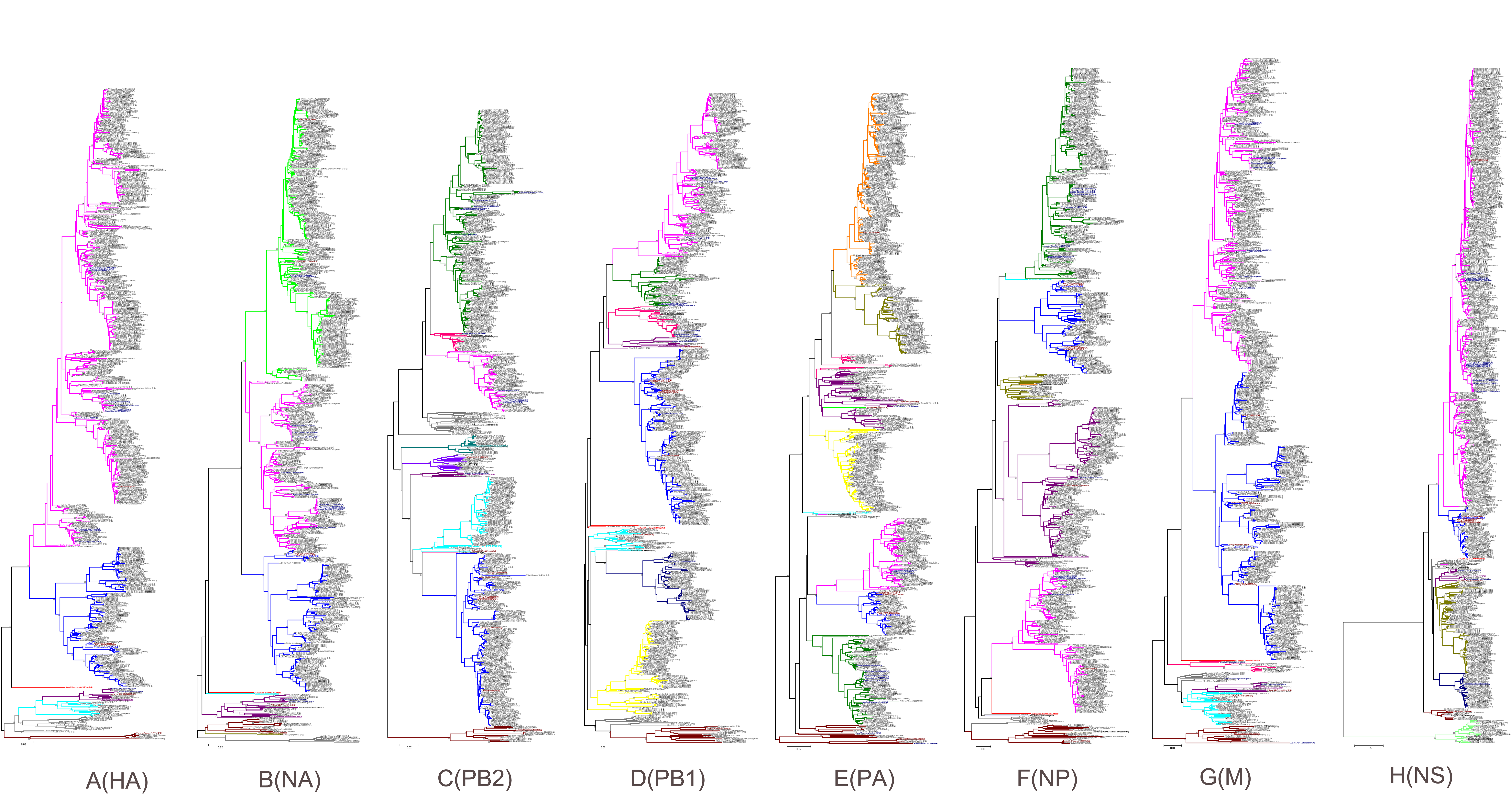

Supplement: Figure S1 — Phylogenetic relationships for HA (A), NA (B), PB2 (C), PB1 (D), PA (E), NP (F), M (G) and NS (H) genes of the 571 analyzed H9N2 influenza viruses. Genome sequences of 571 H9N2 viruses from 1966 to 2009 and other subtype reference viruses were selected from the NCBI Influenza Virus Resource Database. The following nucleotide fragments were used in the phylogenetic analysis: HA, 148 to 1230; NA, 20 to 1390; PB2, 1063 to 2246; PB1, 205 to 1428; PA, 796 to 2016; NP, 76 to 996; M, 80 to 947 and NS, 48 to 831. Neighbor-joining trees were constructed using MEGA and bootstrap values are shown for the key nodes. Clades labeled using different colors indicate major H9N2 virus lineages. Human viruses are colored scarlet, swine viruses in deep blue and avian viruses in black. Representative viruses for each lineage are underlined in the same color as clades, and are highlighted in italics together with human and swine viruses. (TIF) [file pone.0017212.s001.tif]

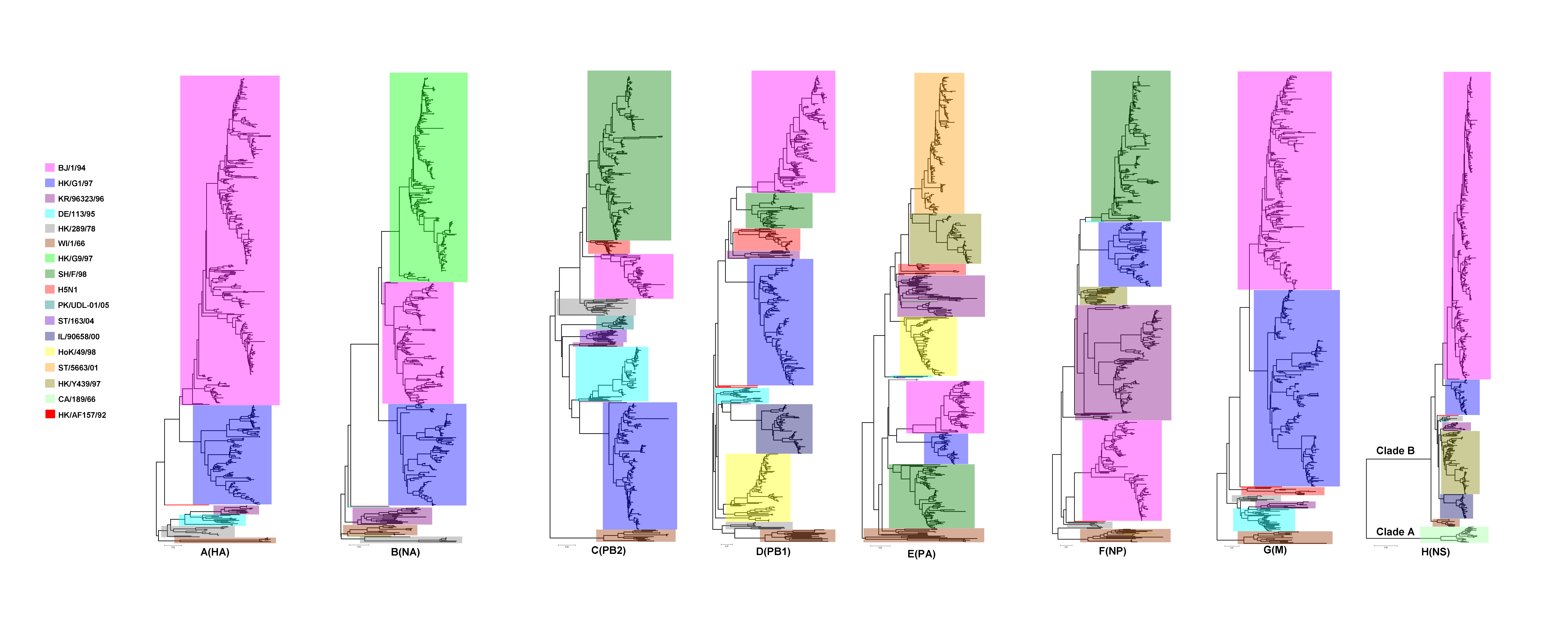

Supplement: Figure S2 — Phylogenetic characteristics of HA (A), NA (B), PB2 (C), PB1 (D), PA (E), NP (F), M (G) and NS (H) genes of H9N2 influenza viruses. 571 H9N2 viral genomic sequences from 1966 to 2009 were characterized. Neighbor-joining phylogenetic trees were generated with MEGA. Clades labeled using different colors indicate major H9N2 virus lineages.Virus abbreviations are listed in the legend of Figure 1. (TIF) [file pone.0017212.s002.tif]
