# Supplementary material for: Phylogenetic Diversity and Genotypical Complexity of H9N2 Influenza A Viruses Revealed by Genomic Sequence Analysis
Source: PLoS One. 2011 Feb 28;6(2):e17212. doi: 10.1371/journal.pone.0017212 (PMC3046171; doi:10.1371/journal.pone.0017212)
Supplement: Table S1 — Gene constellations of different genotypes of the 571 H9N2 influenza A viruses. (DOC) [file pone.0017212.s003.doc]

| **Virus Name** | **Genotype** | **PB2** | **PB1** | **PA** | **HA** | **NP** | **NA** | **MP** | **NS** |
| --- | --- | --- | --- | --- | --- | --- | --- | --- | --- |
| A/quail/Hongkong/G1/97***^a^*** | A0***^b^*** | HK/G1/97 | HK/G1/97 | HK/G1/97 | HK/G1/97 | HK/G1/97 | HK/G1/97 | HK/G1/97 | HK/G1/97 |
| A/Parakeet/Chiba/1/97 | A0 | HK/G1/97 | HK/G1/97 | HK/G1/97 | HK/G1/97 | HK/G1/97 | HK/G1/97 | HK/G1/97 | HK/G1/97 |
| A/Parakeet/Narita/92A/98 | A0 | HK/G1/97 | HK/G1/97 | HK/G1/97 | HK/G1/97 | HK/G1/97 | HK/G1/97 | HK/G1/97 | HK/G1/97 |
| A/Hongkong/1073/99 | A0 | HK/G1/97 | HK/G1/97 | HK/G1/97 | HK/G1/97 | HK/G1/97 | HK/G1/97 | HK/G1/97 | HK/G1/97 |
| A/Hongkong/1074/99 | A0 | HK/G1/97 | HK/G1/97 | HK/G1/97 | HK/G1/97 | HK/G1/97 | HK/G1/97 | HK/G1/97 | HK/G1/97 |
| A/pigeon/Hongkong/FY6/99 | A0 | HK/G1/97 | HK/G1/97 | HK/G1/97 | HK/G1/97 | HK/G1/97 | HK/G1/97 | HK/G1/97 | HK/G1/97 |
| A/chicken/Hongkong/NT16/99 | A0 | HK/G1/97 | HK/G1/97 | HK/G1/97 | HK/G1/97 | HK/G1/97 | HK/G1/97 | HK/G1/97 | HK/G1/97 |
| A/quail/Hongkong/A17/99 | A0 | HK/G1/97 | HK/G1/97 | HK/G1/97 | HK/G1/97 | HK/G1/97 | HK/G1/97 | HK/G1/97 | HK/G1/97 |
| A/chicken/pakistan/2/99 | A0 | HK/G1/97 | HK/G1/97 | HK/G1/97 | HK/G1/97 | HK/G1/97 | HK/G1/97 | HK/G1/97 | HK/G1/97 |
| A/chicken/pakistan/4/99 | A0 | HK/G1/97 | HK/G1/97 | HK/G1/97 | HK/G1/97 | HK/G1/97 | HK/G1/97 | HK/G1/97 | HK/G1/97 |
| A/chicken/pakistan/5/99 | A0 | HK/G1/97 | HK/G1/97 | HK/G1/97 | HK/G1/97 | HK/G1/97 | HK/G1/97 | HK/G1/97 | HK/G1/97 |
| A/quail/Shantou/782/2000 | A0 | HK/G1/97 | HK/G1/97 | HK/G1/97 | HK/G1/97 | HK/G1/97 | HK/G1/97 | HK/G1/97 | HK/G1/97 |
| A/quail/Shantou/1310/2000 | A0 | HK/G1/97 | HK/G1/97 | HK/G1/97 | HK/G1/97 | HK/G1/97 | HK/G1/97 | HK/G1/97 | HK/G1/97 |
| A/quail/Shantou/2061/2000 | A0 | HK/G1/97 | HK/G1/97 | HK/G1/97 | HK/G1/97 | HK/G1/97 | HK/G1/97 | HK/G1/97 | HK/G1/97 |
| A/quail/Shantou/222/2001 | A0 | HK/G1/97 | HK/G1/97 | HK/G1/97 | HK/G1/97 | HK/G1/97 | HK/G1/97 | HK/G1/97 | HK/G1/97 |
| A/quail/Shantou/2111/2001 | A0 | HK/G1/97 | HK/G1/97 | HK/G1/97 | HK/G1/97 | HK/G1/97 | HK/G1/97 | HK/G1/97 | HK/G1/97 |
| A/quail/Shantou/2462/2001 | A0 | HK/G1/97 | HK/G1/97 | HK/G1/97 | HK/G1/97 | HK/G1/97 | HK/G1/97 | HK/G1/97 | HK/G1/97 |
| A/quail/Shantou/4641/2001 | A0 | HK/G1/97 | HK/G1/97 | HK/G1/97 | HK/G1/97 | HK/G1/97 | HK/G1/97 | HK/G1/97 | HK/G1/97 |
| A/Guinea fowl/Shantou/2076/2001 | A0 | HK/G1/97 | HK/G1/97 | HK/G1/97 | HK/G1/97 | HK/G1/97 | HK/G1/97 | HK/G1/97 | HK/G1/97 |
| A/Partridge/Shantou/2875/2001 | A0 | HK/G1/97 | HK/G1/97 | HK/G1/97 | HK/G1/97 | HK/G1/97 | HK/G1/97 | HK/G1/97 | HK/G1/97 |
| A/quail/Shantou/69/2002 | A0 | HK/G1/97 | HK/G1/97 | HK/G1/97 | HK/G1/97 | HK/G1/97 | HK/G1/97 | HK/G1/97 | HK/G1/97 |
| A/quail/Shantou/1551/2002 | A0 | HK/G1/97 | HK/G1/97 | HK/G1/97 | HK/G1/97 | HK/G1/97 | HK/G1/97 | HK/G1/97 | HK/G1/97 |
| A/chicken/Germany/R45/98***^a^*** | A1 | DE/113/95 | Hok/49/98 | KR/96323/96 | HK/G1/97 | HK/Y439/97 | ? | DE/113/95 | HK/Y439/97 |
| A/chicken/Iran/11T/99***^a^*** | A2 | ST/163/04 | Hok/49/98 | KR/96323/96 | HK/G1/97 | HK/Y439/97 | HK/G1/97 | HK/G1/97 | HK/Y439/97 |
| A/chicken/Saudi Arabia/532/99***^a^*** | A3 | DE/113/95 | IL/90658/00 | HK/Y439/97 | HK/G1/97 | KR/96323/96 | HK/G1/97 | HK/G1/97 | IL/90658/00 |
| A/chicken/Israel/90658/2000 | A3 | DE/113/95 | IL/90658/00 | HK/Y439/97 | HK/G1/97 | KR/96323/96 | HK/G1/97 | HK/G1/97 | IL/90658/00 |
| A/turkey/Israel/90710/2000 | A3 | DE/113/95 | IL/90658/00 | HK/Y439/97 | HK/G1/97 | KR/96323/96 | HK/G1/97 | HK/G1/97 | IL/90658/00 |
| A/chicken/Israel/786/2001 | A3 | DE/113/95 | IL/90658/00 | HK/Y439/97 | HK/G1/97 | KR/96323/96 | HK/G1/97 | HK/G1/97 | IL/90658/00 |
| A/turkey/Israel/810/2001 | A3 | DE/113/95 | IL/90658/00 | HK/Y439/97 | HK/G1/97 | KR/96323/96 | HK/G1/97 | HK/G1/97 | IL/90658/00 |
| A/turkey/Israel/619/2002 | A3 | DE/113/95 | IL/90658/00 | HK/Y439/97 | HK/G1/97 | KR/96323/96 | HK/G1/97 | HK/G1/97 | IL/90658/00 |
| A/turkey/Israel/965/2002 | A3 | DE/113/95 | IL/90658/00 | HK/Y439/97 | HK/G1/97 | KR/96323/96 | HK/G1/97 | HK/G1/97 | IL/90658/00 |
| A/turkey/Israel/1013/2002 | A3 | DE/113/95 | IL/90658/00 | HK/Y439/97 | HK/G1/97 | KR/96323/96 | HK/G1/97 | HK/G1/97 | IL/90658/00 |
| A/turkey/Israel/1209/2003 | A3 | DE/113/95 | IL/90658/00 | HK/Y439/97 | HK/G1/97 | KR/96323/96 | HK/G1/97 | HK/G1/97 | IL/90658/00 |
| A/chicken/Israel/1304/2003 | A3 | DE/113/95 | IL/90658/00 | HK/Y439/97 | HK/G1/97 | KR/96323/96 | HK/G1/97 | HK/G1/97 | IL/90658/00 |
| A/chicken/Israel/1376/2003 | A3 | DE/113/95 | IL/90658/00 | HK/Y439/97 | HK/G1/97 | KR/96323/96 | HK/G1/97 | HK/G1/97 | IL/90658/00 |
| A/ostrich/Israel/1436/2003 | A3 | DE/113/95 | IL/90658/00 | HK/Y439/97 | HK/G1/97 | KR/96323/96 | HK/G1/97 | HK/G1/97 | IL/90658/00 |
| A/chicken/Israel/1475/2003 | A3 | DE/113/95 | IL/90658/00 | HK/Y439/97 | HK/G1/97 | KR/96323/96 | HK/G1/97 | HK/G1/97 | IL/90658/00 |
| A/turkey/Israel/1562/2004 | A3 | DE/113/95 | IL/90658/00 | HK/Y439/97 | HK/G1/97 | KR/96323/96 | HK/G1/97 | HK/G1/97 | IL/90658/00 |
| A/turkey/Israel/1567/2004 | A3 | DE/113/95 | IL/90658/00 | HK/Y439/97 | HK/G1/97 | KR/96323/96 | HK/G1/97 | HK/G1/97 | IL/90658/00 |
| A/chicken/Israel/1808/2004 | A3 | DE/113/95 | IL/90658/00 | HK/Y439/97 | HK/G1/97 | KR/96323/96 | HK/G1/97 | HK/G1/97 | IL/90658/00 |
| A/chicken/Israel/1953/2004 | A3 | DE/113/95 | IL/90658/00 | HK/Y439/97 | HK/G1/97 | KR/96323/96 | HK/G1/97 | HK/G1/97 | IL/90658/00 |
| A/chicken/Israel/1966/2004 | A3 | DE/113/95 | IL/90658/00 | HK/Y439/97 | HK/G1/97 | KR/96323/96 | HK/G1/97 | HK/G1/97 | IL/90658/00 |
| A/chicken/Israel/29/2005 | A3 | DE/113/95 | IL/90658/00 | HK/Y439/97 | HK/G1/97 | KR/96323/96 | HK/G1/97 | HK/G1/97 | IL/90658/00 |
| A/turkey/Israel/89/2005 | A3 | DE/113/95 | IL/90658/00 | HK/Y439/97 | HK/G1/97 | KR/96323/96 | HK/G1/97 | HK/G1/97 | IL/90658/00 |
| A/chicken/Israel/282/2005 | A3 | DE/113/95 | IL/90658/00 | HK/Y439/97 | HK/G1/97 | KR/96323/96 | HK/G1/97 | HK/G1/97 | IL/90658/00 |
| A/turkey/Israel/425/2005 | A3 | DE/113/95 | IL/90658/00 | HK/Y439/97 | HK/G1/97 | KR/96323/96 | HK/G1/97 | HK/G1/97 | IL/90658/00 |
| A/chicken/Israel/554/2005 | A3 | DE/113/95 | IL/90658/00 | HK/Y439/97 | HK/G1/97 | KR/96323/96 | HK/G1/97 | HK/G1/97 | IL/90658/00 |
| A/avian/Israel/584/2005 | A3 | DE/113/95 | IL/90658/00 | HK/Y439/97 | HK/G1/97 | KR/96323/96 | HK/G1/97 | HK/G1/97 | IL/90658/00 |
| A/turkey/Israel/747/2005 | A3 | DE/113/95 | IL/90658/00 | HK/Y439/97 | HK/G1/97 | KR/96323/96 | HK/G1/97 | HK/G1/97 | IL/90658/00 |
| A/chicken/Israel/793/2005 | A3 | DE/113/95 | IL/90658/00 | HK/Y439/97 | HK/G1/97 | KR/96323/96 | HK/G1/97 | HK/G1/97 | IL/90658/00 |
| A/chicken/Israel/853/2005 | A3 | DE/113/95 | IL/90658/00 | HK/Y439/97 | HK/G1/97 | KR/96323/96 | HK/G1/97 | HK/G1/97 | IL/90658/00 |
| A/turkey/Israel/884/2005 | A3 | DE/113/95 | IL/90658/00 | HK/Y439/97 | HK/G1/97 | KR/96323/96 | HK/G1/97 | HK/G1/97 | IL/90658/00 |
| A/chicken/Israel/909/2005 | A3 | DE/113/95 | IL/90658/00 | HK/Y439/97 | HK/G1/97 | KR/96323/96 | HK/G1/97 | HK/G1/97 | IL/90658/00 |
| A/chicken/Israel/178/2006 | A3 | DE/113/95 | IL/90658/00 | HK/Y439/97 | HK/G1/97 | KR/96323/96 | HK/G1/97 | HK/G1/97 | IL/90658/00 |
| A/chicken/Israel/375/2007 | A3 | DE/113/95 | IL/90658/00 | HK/Y439/97 | HK/G1/97 | KR/96323/96 | HK/G1/97 | HK/G1/97 | IL/90658/00 |
| A/quail/Shantou/2816/2000***^a^*** | A4 | HK/G1/97 | HK/G1/97 | HK/G1/97 | HK/G1/97 | HK/G1/97 | HK/G1/97 | HK/G1/97 | BJ/1/94 |
| A/quail/Shantou/1235/2001 | A4 | HK/G1/97 | HK/G1/97 | HK/G1/97 | HK/G1/97 | HK/G1/97 | HK/G1/97 | HK/G1/97 | BJ/1/94 |
| A/quail/Shantou/1555/2001 | A4 | HK/G1/97 | HK/G1/97 | HK/G1/97 | HK/G1/97 | HK/G1/97 | HK/G1/97 | HK/G1/97 | BJ/1/94 |
| A/quail/Shantou/1912/2001 | A4 | HK/G1/97 | HK/G1/97 | HK/G1/97 | HK/G1/97 | HK/G1/97 | HK/G1/97 | HK/G1/97 | BJ/1/94 |
| A/quail/Dubai/301/2000***^a^*** | A5 | PK/UDL-01/05 | Hok/49/98 | KR/96323/96 | HK/G1/97 | HK/G1/97 | HK/G1/97 | HK/G1/97 | HK/Y439/97 |
| A/quail/Dubai/302/2000 | A5 | PK/UDL-01/05 | Hok/49/98 | KR/96323/96 | HK/G1/97 | HK/G1/97 | HK/G1/97 | HK/G1/97 | HK/Y439/97 |
| A/quail/Dubai/303/2000 | A5 | PK/UDL-01/05 | Hok/49/98 | KR/96323/96 | HK/G1/97 | HK/G1/97 | HK/G1/97 | HK/G1/97 | HK/Y439/97 |
| A/chicken/Dubai/339/2001 | A5 | PK/UDL-01/05 | Hok/49/98 | KR/96323/96 | HK/G1/97 | HK/G1/97 | HK/G1/97 | HK/G1/97 | HK/Y439/97 |
| A/chicken/Dubai/338/2001 | A5 | PK/UDL-01/05 | Hok/49/98 | KR/96323/96 | HK/G1/97 | HK/G1/97 | HK/G1/97 | HK/G1/97 | HK/Y439/97 |
| A/chicken/Dubai/383/2002 | A5 | PK/UDL-01/05 | Hok/49/98 | KR/96323/96 | HK/G1/97 | HK/G1/97 | HK/G1/97 | HK/G1/97 | HK/Y439/97 |
| A/chicken/Dubai/463/2003 | A5 | PK/UDL-01/05 | Hok/49/98 | KR/96323/96 | HK/G1/97 | HK/G1/97 | HK/G1/97 | HK/G1/97 | HK/Y439/97 |
| A/chicken/Pakistan/UDL-01/2005 | A5 | PK/UDL-01/05 | Hok/49/98 | KR/96323/96 | HK/G1/97 | HK/G1/97 | HK/G1/97 | HK/G1/97 | HK/Y439/97 |
| A/chicken/Pakistan/UDL-02/2005 | A5 | PK/UDL-01/05 | Hok/49/98 | KR/96323/96 | HK/G1/97 | HK/G1/97 | HK/G1/97 | HK/G1/97 | HK/Y439/97 |
| A/chicken/Pakistan/UDL-01/2006 | A5 | PK/UDL-01/05 | Hok/49/98 | KR/96323/96 | HK/G1/97 | HK/G1/97 | HK/G1/97 | HK/G1/97 | HK/Y439/97 |
| A/chicken/Pakistan/UDL-02/2006 | A5 | PK/UDL-01/05 | Hok/49/98 | KR/96323/96 | HK/G1/97 | HK/G1/97 | HK/G1/97 | HK/G1/97 | HK/Y439/97 |
| A/chicken/Pakistan/UDL-04/2006 | A5 | PK/UDL-01/05 | Hok/49/98 | KR/96323/96 | HK/G1/97 | HK/G1/97 | HK/G1/97 | HK/G1/97 | HK/Y439/97 |
| A/chicken/Pakistan/UDL-01/2007 | A5 | PK/UDL-01/05 | Hok/49/98 | KR/96323/96 | HK/G1/97 | HK/G1/97 | HK/G1/97 | HK/G1/97 | HK/Y439/97 |
| A/chicken/Pakistan/UDL-03/2007 | A5 | PK/UDL-01/05 | Hok/49/98 | KR/96323/96 | HK/G1/97 | HK/G1/97 | HK/G1/97 | HK/G1/97 | HK/Y439/97 |
| A/chicken/Pakistan/UDL-04/2007 | A5 | PK/UDL-01/05 | Hok/49/98 | KR/96323/96 | HK/G1/97 | HK/G1/97 | HK/G1/97 | HK/G1/97 | HK/Y439/97 |
| A/chicken/Pakistan/UDL-01/2008 | A5 | PK/UDL-01/05 | Hok/49/98 | KR/96323/96 | HK/G1/97 | HK/G1/97 | HK/G1/97 | HK/G1/97 | HK/Y439/97 |
| A/chicken/Pakistan/UDL-02/2008 | A5 | PK/UDL-01/05 | Hok/49/98 | KR/96323/96 | HK/G1/97 | HK/G1/97 | HK/G1/97 | HK/G1/97 | HK/Y439/97 |
| A/chicken/Pakistan/UDL-03/2008 | A5 | PK/UDL-01/05 | Hok/49/98 | KR/96323/96 | HK/G1/97 | HK/G1/97 | HK/G1/97 | HK/G1/97 | HK/Y439/97 |
| A/quail/Shantou/1242/2001***^a^*** | A6 | HK/G1/97 | HK/G1/97 | HK/G1/97 | HK/G1/97 | HK/G1/97 | HK/G1/97 | BJ/1/94 | BJ/1/94 |
| A/quail/Shantou/3851/2002***^a^*** | A7 | HK/G1/97 | HK/G1/97 | ST/5663/01 | HK/G1/97 | HK/G1/97 | HK/G1/97 | HK/G1/97 | HK/G1/97 |
| A/quail/Shantou/4203/2002 | A7 | HK/G1/97 | HK/G1/97 | ST/5663/01 | HK/G1/97 | HK/G1/97 | HK/G1/97 | HK/G1/97 | HK/G1/97 |
| A/quail/Shantou/4700/2002 | A7 | HK/G1/97 | HK/G1/97 | ST/5663/01 | HK/G1/97 | HK/G1/97 | HK/G1/97 | HK/G1/97 | HK/G1/97 |
| A/quail/Shantou/308/2003 | A7 | HK/G1/97 | HK/G1/97 | ST/5663/01 | HK/G1/97 | HK/G1/97 | HK/G1/97 | HK/G1/97 | HK/G1/97 |
| A/quail/Shantou/335/2003 | A7 | HK/G1/97 | HK/G1/97 | ST/5663/01 | HK/G1/97 | HK/G1/97 | HK/G1/97 | HK/G1/97 | HK/G1/97 |
| A/quail/Shantou/3008/2003 | A7 | HK/G1/97 | HK/G1/97 | ST/5663/01 | HK/G1/97 | HK/G1/97 | HK/G1/97 | HK/G1/97 | HK/G1/97 |
| A/quail/Shantou/3768/2003 | A7 | HK/G1/97 | HK/G1/97 | ST/5663/01 | HK/G1/97 | HK/G1/97 | HK/G1/97 | HK/G1/97 | HK/G1/97 |
| A/quail/Shantou/4044/2003 | A7 | HK/G1/97 | HK/G1/97 | ST/5663/01 | HK/G1/97 | HK/G1/97 | HK/G1/97 | HK/G1/97 | HK/G1/97 |
| A/quail/Shantou/403/2004 | A7 | HK/G1/97 | HK/G1/97 | ST/5663/01 | HK/G1/97 | HK/G1/97 | HK/G1/97 | HK/G1/97 | HK/G1/97 |
| A/quail/Shantou/1475/2004 | A7 | HK/G1/97 | HK/G1/97 | ST/5663/01 | HK/G1/97 | HK/G1/97 | HK/G1/97 | HK/G1/97 | HK/G1/97 |
| A/quail/Shantou/1865/2004 | A7 | HK/G1/97 | HK/G1/97 | ST/5663/01 | HK/G1/97 | HK/G1/97 | HK/G1/97 | HK/G1/97 | HK/G1/97 |
| A/quail/Shantou/1883/2004 | A7 | HK/G1/97 | HK/G1/97 | ST/5663/01 | HK/G1/97 | HK/G1/97 | HK/G1/97 | HK/G1/97 | HK/G1/97 |
| A/quail/Shantou/3060/2004 | A7 | HK/G1/97 | HK/G1/97 | ST/5663/01 | HK/G1/97 | HK/G1/97 | HK/G1/97 | HK/G1/97 | HK/G1/97 |
| A/quail/Shantou/11195/2005 | A7 | HK/G1/97 | HK/G1/97 | ST/5663/01 | HK/G1/97 | HK/G1/97 | HK/G1/97 | HK/G1/97 | HK/G1/97 |
| A/quail/Shantou/13425/2005 | A7 | HK/G1/97 | HK/G1/97 | ST/5663/01 | HK/G1/97 | HK/G1/97 | HK/G1/97 | HK/G1/97 | HK/G1/97 |
| A/quail/Shantou/19506/2005 | A7 | HK/G1/97 | HK/G1/97 | ST/5663/01 | HK/G1/97 | HK/G1/97 | HK/G1/97 | HK/G1/97 | HK/G1/97 |
| A/quail/Shantou/21605/2005 | A7 | HK/G1/97 | HK/G1/97 | ST/5663/01 | HK/G1/97 | HK/G1/97 | HK/G1/97 | HK/G1/97 | HK/G1/97 |
| A/chukkar/Shantou/22116/2005***^a^*** | A8 | HK/G1/97 | SH/F/98 | ST/5663/01 | HK/G1/97 | HK/G1/97 | HK/G1/97 | HK/G1/97 | HK/G1/97 |
| A/chicken/Israel/1525/2006***^a^*** | A9 | DE/113/95 | IL/90658/00 | HK/Y439/97 | HK/G1/97 | KR/96323/96 | HK/G1/97 | HK/G1/97 | HK/Y439/97 |
| A/chicken/Israel/1548/2006 | A9 | DE/113/95 | IL/90658/00 | HK/Y439/97 | HK/G1/97 | KR/96323/96 | HK/G1/97 | HK/G1/97 | HK/Y439/97 |
| A/turkey/Israel/1608/2006 | A9 | DE/113/95 | IL/90658/00 | HK/Y439/97 | HK/G1/97 | KR/96323/96 | HK/G1/97 | HK/G1/97 | HK/Y439/97 |
| A/chicken/Israel/1638/2006 | A9 | DE/113/95 | IL/90658/00 | HK/Y439/97 | HK/G1/97 | KR/96323/96 | HK/G1/97 | HK/G1/97 | HK/Y439/97 |
| A/chicken/Israel/215/2007 | A9 | DE/113/95 | IL/90658/00 | HK/Y439/97 | HK/G1/97 | KR/96323/96 | HK/G1/97 | HK/G1/97 | HK/Y439/97 |
| A/chicken/Israel/386/2007 | A9 | DE/113/95 | IL/90658/00 | HK/Y439/97 | HK/G1/97 | KR/96323/96 | HK/G1/97 | HK/G1/97 | HK/Y439/97 |
| A/chicken/Israel/402/2007 | A9 | DE/113/95 | IL/90658/00 | HK/Y439/97 | HK/G1/97 | KR/96323/96 | HK/G1/97 | HK/G1/97 | HK/Y439/97 |
| A/chicken/Israel/449/2007 | A9 | DE/113/95 | IL/90658/00 | HK/Y439/97 | HK/G1/97 | KR/96323/96 | HK/G1/97 | HK/G1/97 | HK/Y439/97 |
| A/chicken/Israel/728/2007 | A9 | DE/113/95 | IL/90658/00 | HK/Y439/97 | HK/G1/97 | KR/96323/96 | HK/G1/97 | HK/G1/97 | HK/Y439/97 |
| A/chicken/Israel/869/2007 | A9 | DE/113/95 | IL/90658/00 | HK/Y439/97 | HK/G1/97 | KR/96323/96 | HK/G1/97 | HK/G1/97 | HK/Y439/97 |
| A/turkey/Israel/900/2007 | A9 | DE/113/95 | IL/90658/00 | HK/Y439/97 | HK/G1/97 | KR/96323/96 | HK/G1/97 | HK/G1/97 | HK/Y439/97 |
| A/chicken/Israel/933/2007 | A9 | DE/113/95 | IL/90658/00 | HK/Y439/97 | HK/G1/97 | KR/96323/96 | HK/G1/97 | HK/G1/97 | HK/Y439/97 |
| A/chicken/Israel/951/2007 | A9 | DE/113/95 | IL/90658/00 | HK/Y439/97 | HK/G1/97 | KR/96323/96 | HK/G1/97 | HK/G1/97 | HK/Y439/97 |
| A/chicken/Israel/953/2007 | A9 | DE/113/95 | IL/90658/00 | HK/Y439/97 | HK/G1/97 | KR/96323/96 | HK/G1/97 | HK/G1/97 | HK/Y439/97 |
| A/chicken/Israel/1033/2007 | A9 | DE/113/95 | IL/90658/00 | HK/Y439/97 | HK/G1/97 | KR/96323/96 | HK/G1/97 | HK/G1/97 | HK/Y439/97 |
| A/chicken/Israel/1040/2007 | A9 | DE/113/95 | IL/90658/00 | HK/Y439/97 | HK/G1/97 | KR/96323/96 | HK/G1/97 | HK/G1/97 | HK/Y439/97 |
| A/chicken/Israel/54/2008 | A9 | DE/113/95 | IL/90658/00 | HK/Y439/97 | HK/G1/97 | KR/96323/96 | HK/G1/97 | HK/G1/97 | HK/Y439/97 |
| A/chicken/Israel/182/2008 | A9 | DE/113/95 | IL/90658/00 | HK/Y439/97 | HK/G1/97 | KR/96323/96 | HK/G1/97 | HK/G1/97 | HK/Y439/97 |
| A/chicken/Israel/292/2008 | A9 | DE/113/95 | IL/90658/00 | HK/Y439/97 | HK/G1/97 | KR/96323/96 | HK/G1/97 | HK/G1/97 | HK/Y439/97 |
| A/chicken/Israel/310/2008 | A9 | DE/113/95 | IL/90658/00 | HK/Y439/97 | HK/G1/97 | KR/96323/96 | HK/G1/97 | HK/G1/97 | HK/Y439/97 |
| A/avian/Israel/313/2008 | A9 | DE/113/95 | IL/90658/00 | HK/Y439/97 | HK/G1/97 | KR/96323/96 | HK/G1/97 | HK/G1/97 | HK/Y439/97 |
| A/avian/Israel/314/2008 | A9 | DE/113/95 | IL/90658/00 | HK/Y439/97 | HK/G1/97 | KR/96323/96 | HK/G1/97 | HK/G1/97 | HK/Y439/97 |
| A/chicken/Israel/330/2008 | A9 | DE/113/95 | IL/90658/00 | HK/Y439/97 | HK/G1/97 | KR/96323/96 | HK/G1/97 | HK/G1/97 | HK/Y439/97 |
| A/chicken/Israel/524/2008 | A9 | DE/113/95 | IL/90658/00 | HK/Y439/97 | HK/G1/97 | KR/96323/96 | HK/G1/97 | HK/G1/97 | HK/Y439/97 |
| A/turkey/Israel/689/2008 | A9 | DE/113/95 | IL/90658/00 | HK/Y439/97 | HK/G1/97 | KR/96323/96 | HK/G1/97 | HK/G1/97 | HK/Y439/97 |
| A/chicken/Israel/694/2008 | A9 | DE/113/95 | IL/90658/00 | HK/Y439/97 | HK/G1/97 | KR/96323/96 | HK/G1/97 | HK/G1/97 | HK/Y439/97 |
| A/chicken/Israel/702/2008 | A9 | DE/113/95 | IL/90658/00 | HK/Y439/97 | HK/G1/97 | KR/96323/96 | HK/G1/97 | HK/G1/97 | HK/Y439/97 |
| A/chicken/Israel/883/2008 | A9 | DE/113/95 | IL/90658/00 | HK/Y439/97 | HK/G1/97 | KR/96323/96 | HK/G1/97 | HK/G1/97 | HK/Y439/97 |
| A/chicken/Israel/184/2009 | A9 | DE/113/95 | IL/90658/00 | HK/Y439/97 | HK/G1/97 | KR/96323/96 | HK/G1/97 | HK/G1/97 | HK/Y439/97 |
| A/chicken/Beijing/1/94***^a^*** | B0***^b^*** | BJ/1/94 | BJ/1/94 | BJ/1/94 | BJ/1/94 | BJ/1/94 | BJ/1/94 | BJ/1/94 | BJ/1/94 |
| A/chicken/Hong kong/739/94 | B0 | BJ/1/94 | BJ/1/94 | BJ/1/94 | BJ/1/94 | BJ/1/94 | BJ/1/94 | BJ/1/94 | BJ/1/94 |
| A/chicken/Shandong/6/96 | B0 | BJ/1/94 | BJ/1/94 | BJ/1/94 | BJ/1/94 | BJ/1/94 | BJ/1/94 | BJ/1/94 | BJ/1/94 |
| A/chicken/Shandong/7/96 | B0 | BJ/1/94 | BJ/1/94 | BJ/1/94 | BJ/1/94 | BJ/1/94 | BJ/1/94 | BJ/1/94 | BJ/1/94 |
| A/quail/Shanghai/8/96 | B0 | BJ/1/94 | BJ/1/94 | BJ/1/94 | BJ/1/94 | BJ/1/94 | BJ/1/94 | BJ/1/94 | BJ/1/94 |
| A/duck/Nanjing/1/97 | B0 | BJ/1/94 | BJ/1/94 | BJ/1/94 | BJ/1/94 | BJ/1/94 | BJ/1/94 | BJ/1/94 | BJ/1/94 |
| A/duck/Nanjing/2/97 | B0 | BJ/1/94 | BJ/1/94 | BJ/1/94 | BJ/1/94 | BJ/1/94 | BJ/1/94 | BJ/1/94 | BJ/1/94 |
| A/chicken/Guangdong/5/97 | B0 | BJ/1/94 | BJ/1/94 | BJ/1/94 | BJ/1/94 | BJ/1/94 | BJ/1/94 | BJ/1/94 | BJ/1/94 |
| A/chicken/Guangdong/11/97 | B0 | BJ/1/94 | BJ/1/94 | BJ/1/94 | BJ/1/94 | BJ/1/94 | BJ/1/94 | BJ/1/94 | BJ/1/94 |
| A/chicken/Heilongjiang/10/97 | B0 | BJ/1/94 | BJ/1/94 | BJ/1/94 | BJ/1/94 | BJ/1/94 | BJ/1/94 | BJ/1/94 | BJ/1/94 |
| A/chicken/Osaka/aq48/97 | B0 | BJ/1/94 | BJ/1/94 | BJ/1/94 | BJ/1/94 | BJ/1/94 | BJ/1/94 | BJ/1/94 | BJ/1/94 |
| A/duck/Hong kong/FY280/97 | B0 | BJ/1/94 | BJ/1/94 | BJ/1/94 | BJ/1/94 | BJ/1/94 | BJ/1/94 | BJ/1/94 | BJ/1/94 |
| A/chicken/Jiangsu/1/1998 | B0 | BJ/1/94 | BJ/1/94 | BJ/1/94 | BJ/1/94 | BJ/1/94 | BJ/1/94 | BJ/1/94 | BJ/1/94 |
| A/chicken/Shandong/1/1998 | B0 | BJ/1/94 | BJ/1/94 | BJ/1/94 | BJ/1/94 | BJ/1/94 | BJ/1/94 | BJ/1/94 | BJ/1/94 |
| A/chicken/Shijiazhuang/2/98 | B0 | BJ/1/94 | BJ/1/94 | BJ/1/94 | BJ/1/94 | BJ/1/94 | BJ/1/94 | BJ/1/94 | BJ/1/94 |
| A/chicken/Henan/5/98 | B0 | BJ/1/94 | BJ/1/94 | BJ/1/94 | BJ/1/94 | BJ/1/94 | BJ/1/94 | BJ/1/94 | BJ/1/94 |
| A/chicken/Beijing/8/98 | B0 | BJ/1/94 | BJ/1/94 | BJ/1/94 | BJ/1/94 | BJ/1/94 | BJ/1/94 | BJ/1/94 | BJ/1/94 |
| A/swine/Hong kong/9/98 | B0 | BJ/1/94 | BJ/1/94 | BJ/1/94 | BJ/1/94 | BJ/1/94 | BJ/1/94 | BJ/1/94 | BJ/1/94 |
| A/chicken/Shandong/1/1999 | B0 | BJ/1/94 | BJ/1/94 | BJ/1/94 | BJ/1/94 | BJ/1/94 | BJ/1/94 | BJ/1/94 | BJ/1/94 |
| A/chicken/Shijiazhuang/2/99 | B0 | BJ/1/94 | BJ/1/94 | BJ/1/94 | BJ/1/94 | BJ/1/94 | BJ/1/94 | BJ/1/94 | BJ/1/94 |
| A/chicken/Gansu/2/99 | B0 | BJ/1/94 | BJ/1/94 | BJ/1/94 | BJ/1/94 | BJ/1/94 | BJ/1/94 | BJ/1/94 | BJ/1/94 |
| A/chicken/Ningxia/4/99 | B0 | BJ/1/94 | BJ/1/94 | BJ/1/94 | BJ/1/94 | BJ/1/94 | BJ/1/94 | BJ/1/94 | BJ/1/94 |
| A/chicken/Ningxia/5/99 | B0 | BJ/1/94 | BJ/1/94 | BJ/1/94 | BJ/1/94 | BJ/1/94 | BJ/1/94 | BJ/1/94 | BJ/1/94 |
| A/chicken/Hong kong/SF2/99 | B0 | BJ/1/94 | BJ/1/94 | BJ/1/94 | BJ/1/94 | BJ/1/94 | BJ/1/94 | BJ/1/94 | BJ/1/94 |
| A/chicken/Hong kong/KC12/99 | B0 | BJ/1/94 | BJ/1/94 | BJ/1/94 | BJ/1/94 | BJ/1/94 | BJ/1/94 | BJ/1/94 | BJ/1/94 |
| A/chicken/Hong kong/FY20/99 | B0 | BJ/1/94 | BJ/1/94 | BJ/1/94 | BJ/1/94 | BJ/1/94 | BJ/1/94 | BJ/1/94 | BJ/1/94 |
| A/quail/Hong kong/NT28/99 | B0 | BJ/1/94 | BJ/1/94 | BJ/1/94 | BJ/1/94 | BJ/1/94 | BJ/1/94 | BJ/1/94 | BJ/1/94 |
| A/silky chicken/Hong Kong/SF44/99 | B0 | BJ/1/94 | BJ/1/94 | BJ/1/94 | BJ/1/94 | BJ/1/94 | BJ/1/94 | BJ/1/94 | BJ/1/94 |
| A/chicken/Shandong/1/2000 | B0 | BJ/1/94 | BJ/1/94 | BJ/1/94 | BJ/1/94 | BJ/1/94 | BJ/1/94 | BJ/1/94 | BJ/1/94 |
| A/chicken/Guangdong/4/00 | B0 | BJ/1/94 | BJ/1/94 | BJ/1/94 | BJ/1/94 | BJ/1/94 | BJ/1/94 | BJ/1/94 | BJ/1/94 |
| A/chicken/Fujian/25/00 | B0 | BJ/1/94 | BJ/1/94 | BJ/1/94 | BJ/1/94 | BJ/1/94 | BJ/1/94 | BJ/1/94 | BJ/1/94 |
| A/chicken/Hebei/31/00 | B0 | BJ/1/94 | BJ/1/94 | BJ/1/94 | BJ/1/94 | BJ/1/94 | BJ/1/94 | BJ/1/94 | BJ/1/94 |
| A/Pigeon/Nanchang/2-0461/2000 | B0 | BJ/1/94 | BJ/1/94 | BJ/1/94 | BJ/1/94 | BJ/1/94 | BJ/1/94 | BJ/1/94 | BJ/1/94 |
| A/Wild Duck/Nanchang/2-0480/2000 | B0 | BJ/1/94 | BJ/1/94 | BJ/1/94 | BJ/1/94 | BJ/1/94 | BJ/1/94 | BJ/1/94 | BJ/1/94 |
| A/chicken/Hebei/B1/2001 | B0 | BJ/1/94 | BJ/1/94 | BJ/1/94 | BJ/1/94 | BJ/1/94 | BJ/1/94 | BJ/1/94 | BJ/1/94 |
| A/chicken/YoKohama/aq45/2002 | B0 | BJ/1/94 | BJ/1/94 | BJ/1/94 | BJ/1/94 | BJ/1/94 | BJ/1/94 | BJ/1/94 | BJ/1/94 |
| A/chicken/Tianjin/B1/2004 | B0 | BJ/1/94 | BJ/1/94 | BJ/1/94 | BJ/1/94 | BJ/1/94 | BJ/1/94 | BJ/1/94 | BJ/1/94 |
| A/duck/Hubei/WI/2004 | B0 | BJ/1/94 | BJ/1/94 | BJ/1/94 | BJ/1/94 | BJ/1/94 | BJ/1/94 | BJ/1/94 | BJ/1/94 |
| A/chicken/Beijing/L1/2005 | B0 | BJ/1/94 | BJ/1/94 | BJ/1/94 | BJ/1/94 | BJ/1/94 | BJ/1/94 | BJ/1/94 | BJ/1/94 |
| A/chicken/Hebei/L1/2006 | B0 | BJ/1/94 | BJ/1/94 | BJ/1/94 | BJ/1/94 | BJ/1/94 | BJ/1/94 | BJ/1/94 | BJ/1/94 |
| A/chicken/Hongkong/G9/97***^a^*** | B1 | HK/G1/97 | HK/G1/97 | BJ/1/94 | BJ/1/94 | BJ/1/94 | HK/G9/97 | BJ/1/94 | BJ/1/94 |
| A/chicken/Guangdong/6/97 | B1 | HK/G1/97 | HK/G1/97 | BJ/1/94 | BJ/1/94 | BJ/1/94 | HK/G9/97 | BJ/1/94 | BJ/1/94 |
| A/chicken/Shenzhen/9/97 | B1 | HK/G1/97 | HK/G1/97 | BJ/1/94 | BJ/1/94 | BJ/1/94 | HK/G9/97 | BJ/1/94 | BJ/1/94 |
| A/pigeon/Hongkong/Y233/97 | B1 | HK/G1/97 | HK/G1/97 | BJ/1/94 | BJ/1/94 | BJ/1/94 | HK/G9/97 | BJ/1/94 | BJ/1/94 |
| A/chicken/Hongkong/G23/99 | B1 | HK/G1/97 | HK/G1/97 | BJ/1/94 | BJ/1/94 | BJ/1/94 | HK/G9/97 | BJ/1/94 | BJ/1/94 |
| A/chicken/Sichuan/5/97***^a^*** | B2 | HK/G1/97 | HK/G1/97 | HK/G1/97 | BJ/1/94 | BJ/1/94 | HK/G9/97 | HK/G1/97 | BJ/1/94 |
| A/Guangzhou/333/99 | B2 | HK/G1/97 | HK/G1/97 | HK/G1/97 | BJ/1/94 | BJ/1/94 | HK/G9/97 | HK/G1/97 | BJ/1/94 |
| A/quail/Shantou/243/2000 | B2 | HK/G1/97 | HK/G1/97 | HK/G1/97 | BJ/1/94 | BJ/1/94 | HK/G9/97 | HK/G1/97 | BJ/1/94 |
| A/duck/Shantou/1043/2000 | B2 | HK/G1/97 | HK/G1/97 | HK/G1/97 | BJ/1/94 | BJ/1/94 | HK/G9/97 | HK/G1/97 | BJ/1/94 |
| A/chicken/Shanghai/F/98***^a^*** | B3 | SH/F/98 | SH/F/98 | SH/F/98 | BJ/1/94 | SH/F/98 | BJ/1/94 | BJ/1/94 | BJ/1/94 |
| A/chicken/Shanghai/2/1999 | B3 | SH/F/98 | SH/F/98 | SH/F/98 | BJ/1/94 | SH/F/98 | BJ/1/94 | BJ/1/94 | BJ/1/94 |
| A/chicken/Shanghai/3/2000 | B3 | SH/F/98 | SH/F/98 | SH/F/98 | BJ/1/94 | SH/F/98 | BJ/1/94 | BJ/1/94 | BJ/1/94 |
| A/chicken/Shanghai/1/2001 | B3 | SH/F/98 | SH/F/98 | SH/F/98 | BJ/1/94 | SH/F/98 | BJ/1/94 | BJ/1/94 | BJ/1/94 |
| A/chicken/Shanghai/2/2001 | B3 | SH/F/98 | SH/F/98 | SH/F/98 | BJ/1/94 | SH/F/98 | BJ/1/94 | BJ/1/94 | BJ/1/94 |
| A/chicken/Shanghai/7/2001 | B3 | SH/F/98 | SH/F/98 | SH/F/98 | BJ/1/94 | SH/F/98 | BJ/1/94 | BJ/1/94 | BJ/1/94 |
| A/chicken/Shanghai/10/01 | B3 | SH/F/98 | SH/F/98 | SH/F/98 | BJ/1/94 | SH/F/98 | BJ/1/94 | BJ/1/94 | BJ/1/94 |
| A/chicken/Shanghai/14/2001 | B3 | SH/F/98 | SH/F/98 | SH/F/98 | BJ/1/94 | SH/F/98 | BJ/1/94 | BJ/1/94 | BJ/1/94 |
| A/chicken/Shanghai/16/2001 | B3 | SH/F/98 | SH/F/98 | SH/F/98 | BJ/1/94 | SH/F/98 | BJ/1/94 | BJ/1/94 | BJ/1/94 |
| A/chicken/Jiangsu/2/2001 | B3 | SH/F/98 | SH/F/98 | SH/F/98 | BJ/1/94 | SH/F/98 | BJ/1/94 | BJ/1/94 | BJ/1/94 |
| A/chicken/Yunnan/nh/2001 | B3 | SH/F/98 | SH/F/98 | SH/F/98 | BJ/1/94 | SH/F/98 | BJ/1/94 | BJ/1/94 | BJ/1/94 |
| A/chicken/Henan/ni/2001 | B3 | SH/F/98 | SH/F/98 | SH/F/98 | BJ/1/94 | SH/F/98 | BJ/1/94 | BJ/1/94 | BJ/1/94 |
| A/chicken/Helongjiang/48/01 | B3 | SH/F/98 | SH/F/98 | SH/F/98 | BJ/1/94 | SH/F/98 | BJ/1/94 | BJ/1/94 | BJ/1/94 |
| A/chicken/Osaka/aq69/2001 | B3 | SH/F/98 | SH/F/98 | SH/F/98 | BJ/1/94 | SH/F/98 | BJ/1/94 | BJ/1/94 | BJ/1/94 |
| A/chicken/Henan/1/2002 | B3 | SH/F/98 | SH/F/98 | SH/F/98 | BJ/1/94 | SH/F/98 | BJ/1/94 | BJ/1/94 | BJ/1/94 |
| A/chicken/Shanghai/1/2002 | B3 | SH/F/98 | SH/F/98 | SH/F/98 | BJ/1/94 | SH/F/98 | BJ/1/94 | BJ/1/94 | BJ/1/94 |
| A/chicken/Shanghai/2/2002 | B3 | SH/F/98 | SH/F/98 | SH/F/98 | BJ/1/94 | SH/F/98 | BJ/1/94 | BJ/1/94 | BJ/1/94 |
| A/chicken/Jiangsu/1/2002 | B3 | SH/F/98 | SH/F/98 | SH/F/98 | BJ/1/94 | SH/F/98 | BJ/1/94 | BJ/1/94 | BJ/1/94 |
| A/chicken/Anhui/2/2002 | B3 | SH/F/98 | SH/F/98 | SH/F/98 | BJ/1/94 | SH/F/98 | BJ/1/94 | BJ/1/94 | BJ/1/94 |
| A/duck/Zhejiang/3/2002 | B3 | SH/F/98 | SH/F/98 | SH/F/98 | BJ/1/94 | SH/F/98 | BJ/1/94 | BJ/1/94 | BJ/1/94 |
| A/chicken/Beijing/nl/2002 | B3 | SH/F/98 | SH/F/98 | SH/F/98 | BJ/1/94 | SH/F/98 | BJ/1/94 | BJ/1/94 | BJ/1/94 |
| A/chicken/Hebei/nj/2002 | B3 | SH/F/98 | SH/F/98 | SH/F/98 | BJ/1/94 | SH/F/98 | BJ/1/94 | BJ/1/94 | BJ/1/94 |
| A/chicken/Hunan/774/2002 | B3 | SH/F/98 | SH/F/98 | SH/F/98 | BJ/1/94 | SH/F/98 | BJ/1/94 | BJ/1/94 | BJ/1/94 |
| A/chicken/Henan/1/2003 | B3 | SH/F/98 | SH/F/98 | SH/F/98 | BJ/1/94 | SH/F/98 | BJ/1/94 | BJ/1/94 | BJ/1/94 |
| A/swine/Shandong/na/2003 | B3 | SH/F/98 | SH/F/98 | SH/F/98 | BJ/1/94 | SH/F/98 | BJ/1/94 | BJ/1/94 | BJ/1/94 |
| A/swine/Shandong/W4/2003 | B3 | SH/F/98 | SH/F/98 | SH/F/98 | BJ/1/94 | SH/F/98 | BJ/1/94 | BJ/1/94 | BJ/1/94 |
| A/chicken/Jiangsu/1/2004 | B3 | SH/F/98 | SH/F/98 | SH/F/98 | BJ/1/94 | SH/F/98 | BJ/1/94 | BJ/1/94 | BJ/1/94 |
| A/chicken/Henan/1/2004 | B3 | SH/F/98 | SH/F/98 | SH/F/98 | BJ/1/94 | SH/F/98 | BJ/1/94 | BJ/1/94 | BJ/1/94 |
| A/chicken/Henan/2/2004 | B3 | SH/F/98 | SH/F/98 | SH/F/98 | BJ/1/94 | SH/F/98 | BJ/1/94 | BJ/1/94 | BJ/1/94 |
| A/chicken/Shandong/1/2004 | B3 | SH/F/98 | SH/F/98 | SH/F/98 | BJ/1/94 | SH/F/98 | BJ/1/94 | BJ/1/94 | BJ/1/94 |
| A/chicken/Jiangsu/L1/2004 | B3 | SH/F/98 | SH/F/98 | SH/F/98 | BJ/1/94 | SH/F/98 | BJ/1/94 | BJ/1/94 | BJ/1/94 |
| A/chicken/Henan/1/2005 | B3 | SH/F/98 | SH/F/98 | SH/F/98 | BJ/1/94 | SH/F/98 | BJ/1/94 | BJ/1/94 | BJ/1/94 |
| A/swine/Shandong/nc/2005 | B3 | SH/F/98 | SH/F/98 | SH/F/98 | BJ/1/94 | SH/F/98 | BJ/1/94 | BJ/1/94 | BJ/1/94 |
| A/chicken/Guangxi/2389/2005 | B3 | SH/F/98 | SH/F/98 | SH/F/98 | BJ/1/94 | SH/F/98 | BJ/1/94 | BJ/1/94 | BJ/1/94 |
| A/chicken/Shandong/B3/2007 | B3 | SH/F/98 | SH/F/98 | SH/F/98 | BJ/1/94 | SH/F/98 | BJ/1/94 | BJ/1/94 | BJ/1/94 |
| A/chicken/Henan/nd/1998***^a^*** | B4 | SH/F/98 | SH/F/98 | SH/F/98 | BJ/1/94 | SH/F/98 | BJ/1/94 | H5N1 | HK/Y439/97 |
| A/chicken/Beijing/ne/1999***^a^*** | B5 | SH/F/98 | BJ/1/94 | SH/F/98 | BJ/1/94 | SH/F/98 | BJ/1/94 | BJ/1/94 | BJ/1/94 |
| A/duck/jiangsu/nf/2002 | B5 | SH/F/98 | BJ/1/94 | SH/F/98 | BJ/1/94 | SH/F/98 | BJ/1/94 | BJ/1/94 | BJ/1/94 |
| A/Gf/HK/NT101/2003 | B5 | SH/F/98 | BJ/1/94 | SH/F/98 | BJ/1/94 | SH/F/98 | BJ/1/94 | BJ/1/94 | BJ/1/94 |
| A/CK/HK/CSW161/2003 | B5 | SH/F/98 | BJ/1/94 | SH/F/98 | BJ/1/94 | SH/F/98 | BJ/1/94 | BJ/1/94 | BJ/1/94 |
| A/CK/HK/YU577/2003 | B5 | SH/F/98 | BJ/1/94 | SH/F/98 | BJ/1/94 | SH/F/98 | BJ/1/94 | BJ/1/94 | BJ/1/94 |
| A/SCK/HK/YU663/2003 | B5 | SH/F/98 | BJ/1/94 | SH/F/98 | BJ/1/94 | SH/F/98 | BJ/1/94 | BJ/1/94 | BJ/1/94 |
| A/chicken/HongKong/AP45/03 | B5 | SH/F/98 | BJ/1/94 | SH/F/98 | BJ/1/94 | SH/F/98 | BJ/1/94 | BJ/1/94 | BJ/1/94 |
| A/pheasant/HongKong/WF54/03 | B5 | SH/F/98 | BJ/1/94 | SH/F/98 | BJ/1/94 | SH/F/98 | BJ/1/94 | BJ/1/94 | BJ/1/94 |
| A/chicken/HongKong/BD90/03 | B5 | SH/F/98 | BJ/1/94 | SH/F/98 | BJ/1/94 | SH/F/98 | BJ/1/94 | BJ/1/94 | BJ/1/94 |
| A/chicken/HongKong/CSW153/03 | B5 | SH/F/98 | BJ/1/94 | SH/F/98 | BJ/1/94 | SH/F/98 | BJ/1/94 | BJ/1/94 | BJ/1/94 |
| A/chicken/Jiangsu/1/1999***^a^*** | B6 | SH/F/98 | BJ/1/94 | SH/F/98 | BJ/1/94 | SH/F/98 | BJ/1/94 | BJ/1/94 | HK/Y439/97 |
| A/chicken/Guangxi/4/1999***^a^*** | B7 | HK/G1/97 | BJ/1/94 | Hok/49/98 | BJ/1/94 | HK/G1/97 | HK/G9/97 | BJ/1/94 | BJ/1/94 |
| A/chicken/Guangxi/9/99***^a^*** | B8 | HK/G1/97 | HK/G1/97 | HK/G1/97 | BJ/1/94 | BJ/1/94 | BJ/1/94 | BJ/1/94 | HK/G1/97 |
| A/chicken/Guangxi/10/99***^a^*** | B9 | HK/G1/97 | HK/G1/97 | HK/G1/97 | BJ/1/94 | BJ/1/94 | HK/G9/97 | BJ/1/94 | HK/G1/97 |
| A/chicken/Guangdong/10/00***^a^*** | B10 | BJ/1/94 | BJ/1/94 | BJ/1/94 | BJ/1/94 | BJ/1/94 | HK/G9/97 | BJ/1/94 | BJ/1/94 |
| A/chicken/Jiangsu/1/00 | B10 | BJ/1/94 | BJ/1/94 | BJ/1/94 | BJ/1/94 | BJ/1/94 | HK/G9/97 | BJ/1/94 | BJ/1/94 |
| A/chicken/Guangdong/47/01 | B10 | BJ/1/94 | BJ/1/94 | BJ/1/94 | BJ/1/94 | BJ/1/94 | HK/G9/97 | BJ/1/94 | BJ/1/94 |
| A/chicken/Jilin/53/01 | B10 | BJ/1/94 | BJ/1/94 | BJ/1/94 | BJ/1/94 | BJ/1/94 | HK/G9/97 | BJ/1/94 | BJ/1/94 |
| A/chicken/Shantou/1126/2001 | B10 | BJ/1/94 | BJ/1/94 | BJ/1/94 | BJ/1/94 | BJ/1/94 | HK/G9/97 | BJ/1/94 | BJ/1/94 |
| A/chicken/Shantou/1205/2001 | B10 | BJ/1/94 | BJ/1/94 | BJ/1/94 | BJ/1/94 | BJ/1/94 | HK/G9/97 | BJ/1/94 | BJ/1/94 |
| A/chicken/Shantou/3173/2001 | B10 | BJ/1/94 | BJ/1/94 | BJ/1/94 | BJ/1/94 | BJ/1/94 | HK/G9/97 | BJ/1/94 | BJ/1/94 |
| A/chicken/YoKohama/aq120/2001 | B10 | BJ/1/94 | BJ/1/94 | BJ/1/94 | BJ/1/94 | BJ/1/94 | HK/G9/97 | BJ/1/94 | BJ/1/94 |
| A/chicken/YoKohama/aq144/2001 | B10 | BJ/1/94 | BJ/1/94 | BJ/1/94 | BJ/1/94 | BJ/1/94 | HK/G9/97 | BJ/1/94 | BJ/1/94 |
| A/chicken/Guangdong/21/02 | B10 | BJ/1/94 | BJ/1/94 | BJ/1/94 | BJ/1/94 | BJ/1/94 | HK/G9/97 | BJ/1/94 | BJ/1/94 |
| A/chicken/Henan/43/02 | B10 | BJ/1/94 | BJ/1/94 | BJ/1/94 | BJ/1/94 | BJ/1/94 | HK/G9/97 | BJ/1/94 | BJ/1/94 |
| A/chicken/HongKong/SF1/03 | B10 | BJ/1/94 | BJ/1/94 | BJ/1/94 | BJ/1/94 | BJ/1/94 | HK/G9/97 | BJ/1/94 | BJ/1/94 |
| A/chicken/HongKong/FY23/03 | B10 | BJ/1/94 | BJ/1/94 | BJ/1/94 | BJ/1/94 | BJ/1/94 | HK/G9/97 | BJ/1/94 | BJ/1/94 |
| A/chicken/HongKong/WF120/03 | B10 | BJ/1/94 | BJ/1/94 | BJ/1/94 | BJ/1/94 | BJ/1/94 | HK/G9/97 | BJ/1/94 | BJ/1/94 |
| A/CK/HK/WF126/2003 | B10 | BJ/1/94 | BJ/1/94 | BJ/1/94 | BJ/1/94 | BJ/1/94 | HK/G9/97 | BJ/1/94 | BJ/1/94 |
| A/SCK/HK/WF285/2003 | B10 | BJ/1/94 | BJ/1/94 | BJ/1/94 | BJ/1/94 | BJ/1/94 | HK/G9/97 | BJ/1/94 | BJ/1/94 |
| A/swine/Jiangxi/1/2004 | B10 | BJ/1/94 | BJ/1/94 | BJ/1/94 | BJ/1/94 | BJ/1/94 | HK/G9/97 | BJ/1/94 | BJ/1/94 |
| A/swine/Jiangxi/wx2/2004 | B10 | BJ/1/94 | BJ/1/94 | BJ/1/94 | BJ/1/94 | BJ/1/94 | HK/G9/97 | BJ/1/94 | BJ/1/94 |
| A/chicken/Henan/26/00***^a^*** | B11 | HK/G1/97 | BJ/1/94 | BJ/1/94 | BJ/1/94 | BJ/1/94 | BJ/1/94 | BJ/1/94 | BJ/1/94 |
| A/chicken/Henan/62/00***^a^*** | B12 | HK/G1/97 | BJ/1/94 | HK/G1/97 | BJ/1/94 | BJ/1/94 | BJ/1/94 | BJ/1/94 | BJ/1/94 |
| A/chicken/china/Guangxi1/2000***^a^*** | B13 | H5N1 | BJ/1/94 | H5N1 | BJ/1/94 | BJ/1/94 | HK/G9/97 | BJ/1/94 | BJ/1/94 |
| A/quail/Nanchang/2-0460/2000***^a^*** | B14 | SH/F/98 | BJ/1/94 | Hok/49/98 | BJ/1/94 | KR/96323/96 | BJ/1/94 | BJ/1/94 | BJ/1/94 |
| A/duck/Shantou/2102/00***^a^*** | B15 | SH/F/98 | BJ/1/94 | Hok/49/98 | BJ/1/94 | SH/F/98 | HK/G9/97 | BJ/1/94 | BJ/1/94 |
| A/chicken/Shantou/94/2000***^a^*** | B16 | SH/F/98 | Hok/49/98 | Hok/49/98 | BJ/1/94 | KR/96323/96 | BJ/1/94 | BJ/1/94 | BJ/1/94 |
| A/chicken/Shantou/212/2000 | B16 | SH/F/98 | Hok/49/98 | Hok/49/98 | BJ/1/94 | KR/96323/96 | BJ/1/94 | BJ/1/94 | BJ/1/94 |
| A/chicken/Shantou/859/2000 | B16 | SH/F/98 | Hok/49/98 | Hok/49/98 | BJ/1/94 | KR/96323/96 | BJ/1/94 | BJ/1/94 | BJ/1/94 |
| A/chicken/Shantou/1322/2000 | B16 | SH/F/98 | Hok/49/98 | Hok/49/98 | BJ/1/94 | KR/96323/96 | BJ/1/94 | BJ/1/94 | BJ/1/94 |
| A/Guinea fowl/Shantou/1677/2000 | B16 | SH/F/98 | Hok/49/98 | Hok/49/98 | BJ/1/94 | KR/96323/96 | BJ/1/94 | BJ/1/94 | BJ/1/94 |
| A/chicken/Shantou/1690/2000 | B16 | SH/F/98 | Hok/49/98 | Hok/49/98 | BJ/1/94 | KR/96323/96 | BJ/1/94 | BJ/1/94 | BJ/1/94 |
| A/quail/Shantou/1820/2000 | B16 | SH/F/98 | Hok/49/98 | Hok/49/98 | BJ/1/94 | KR/96323/96 | BJ/1/94 | BJ/1/94 | BJ/1/94 |
| A/quail/Shantou/850/2001 | B16 | SH/F/98 | Hok/49/98 | Hok/49/98 | BJ/1/94 | KR/96323/96 | BJ/1/94 | BJ/1/94 | BJ/1/94 |
| A/chicken/Nanchang/4-301/2001 | B16 | SH/F/98 | Hok/49/98 | Hok/49/98 | BJ/1/94 | KR/96323/96 | BJ/1/94 | BJ/1/94 | BJ/1/94 |
| A/duck/Nanchang/4-361/2001 | B16 | SH/F/98 | Hok/49/98 | Hok/49/98 | BJ/1/94 | KR/96323/96 | BJ/1/94 | BJ/1/94 | BJ/1/94 |
| A/chicken/Shantou/4342/2002 | B16 | SH/F/98 | Hok/49/98 | Hok/49/98 | BJ/1/94 | KR/96323/96 | BJ/1/94 | BJ/1/94 | BJ/1/94 |
| A/silky chicken/Shantou/1826/2004 | B16 | SH/F/98 | Hok/49/98 | Hok/49/98 | BJ/1/94 | KR/96323/96 | BJ/1/94 | BJ/1/94 | BJ/1/94 |
| A/chicken/Shantou/1926/2004 | B16 | SH/F/98 | Hok/49/98 | Hok/49/98 | BJ/1/94 | KR/96323/96 | BJ/1/94 | BJ/1/94 | BJ/1/94 |
| A/chicken/Shantou/2692/2004 | B16 | SH/F/98 | Hok/49/98 | Hok/49/98 | BJ/1/94 | KR/96323/96 | BJ/1/94 | BJ/1/94 | BJ/1/94 |
| A/chicken/Shantou/2994/2004 | B16 | SH/F/98 | Hok/49/98 | Hok/49/98 | BJ/1/94 | KR/96323/96 | BJ/1/94 | BJ/1/94 | BJ/1/94 |
| A/silky chicken/Shantou/3581/2005 | B16 | SH/F/98 | Hok/49/98 | Hok/49/98 | BJ/1/94 | KR/96323/96 | BJ/1/94 | BJ/1/94 | BJ/1/94 |
| A/duck/Shantou/830/00***^a^*** | B17 | SH/F/98 | BJ/1/94 | Hok/49/98 | BJ/1/94 | KR/96323/96 | HK/G9/97 | BJ/1/94 | BJ/1/94 |
| A/chicken/Shantou/1610/2001 | B17 | SH/F/98 | BJ/1/94 | Hok/49/98 | BJ/1/94 | KR/96323/96 | HK/G9/97 | BJ/1/94 | BJ/1/94 |
| A/quail/Shantou/1158/2001 | B17 | SH/F/98 | BJ/1/94 | Hok/49/98 | BJ/1/94 | KR/96323/96 | HK/G9/97 | BJ/1/94 | BJ/1/94 |
| A/chicken/Shantou/1608/2001 | B17 | SH/F/98 | BJ/1/94 | Hok/49/98 | BJ/1/94 | KR/96323/96 | HK/G9/97 | BJ/1/94 | BJ/1/94 |
| A/quail/Shantou/1318/2000***^a^*** | B18 | SH/F/98 | Hok/49/98 | Hok/49/98 | BJ/1/94 | KR/96323/96 | BJ/1/94 | HK/G1/97 | BJ/1/94 |
| A/silky chicken/Shantou/1818/2000***^a^*** | B19 | SH/F/98 | Hok/49/98 | Hok/49/98 | BJ/1/94 | KR/96323/96 | HK/G9/97 | BJ/1/94 | BJ/1/94 |
| A/duck/Shantou/1881/2000 | B19 | SH/F/98 | Hok/49/98 | Hok/49/98 | BJ/1/94 | KR/96323/96 | HK/G9/97 | BJ/1/94 | BJ/1/94 |
| A/chicken/Shantou/2098/2000 | B19 | SH/F/98 | Hok/49/98 | Hok/49/98 | BJ/1/94 | KR/96323/96 | HK/G9/97 | BJ/1/94 | BJ/1/94 |
| A/duck/Shantou/2143/2000 | B19 | SH/F/98 | Hok/49/98 | Hok/49/98 | BJ/1/94 | KR/96323/96 | HK/G9/97 | BJ/1/94 | BJ/1/94 |
| A/duck/Shantou/2144/2000 | B19 | SH/F/98 | Hok/49/98 | Hok/49/98 | BJ/1/94 | KR/96323/96 | HK/G9/97 | BJ/1/94 | BJ/1/94 |
| A/chicken/Shantou/1890/2001 | B19 | SH/F/98 | Hok/49/98 | Hok/49/98 | BJ/1/94 | KR/96323/96 | HK/G9/97 | BJ/1/94 | BJ/1/94 |
| A/duck/Shantou/2088/01 | B19 | SH/F/98 | Hok/49/98 | Hok/49/98 | BJ/1/94 | KR/96323/96 | HK/G9/97 | BJ/1/94 | BJ/1/94 |
| A/silky chicken/Shantou/2619/2001 | B19 | SH/F/98 | Hok/49/98 | Hok/49/98 | BJ/1/94 | KR/96323/96 | HK/G9/97 | BJ/1/94 | BJ/1/94 |
| A/chicken/Shantou/2712/2001 | B19 | SH/F/98 | Hok/49/98 | Hok/49/98 | BJ/1/94 | KR/96323/96 | HK/G9/97 | BJ/1/94 | BJ/1/94 |
| A/duck/Shantou/3549/2001 | B19 | SH/F/98 | Hok/49/98 | Hok/49/98 | BJ/1/94 | KR/96323/96 | HK/G9/97 | BJ/1/94 | BJ/1/94 |
| A/Partridge/Shantou/3720/2001 | B19 | SH/F/98 | Hok/49/98 | Hok/49/98 | BJ/1/94 | KR/96323/96 | HK/G9/97 | BJ/1/94 | BJ/1/94 |
| A/chicken/Shantou/3778/2001 | B19 | SH/F/98 | Hok/49/98 | Hok/49/98 | BJ/1/94 | KR/96323/96 | HK/G9/97 | BJ/1/94 | BJ/1/94 |
| A/chicken/Shantou/4208/2001 | B19 | SH/F/98 | Hok/49/98 | Hok/49/98 | BJ/1/94 | KR/96323/96 | HK/G9/97 | BJ/1/94 | BJ/1/94 |
| A/chicken/Shantou/5028/2001 | B19 | SH/F/98 | Hok/49/98 | Hok/49/98 | BJ/1/94 | KR/96323/96 | HK/G9/97 | BJ/1/94 | BJ/1/94 |
| A/chicken/Shantou/5319/2001 | B19 | SH/F/98 | Hok/49/98 | Hok/49/98 | BJ/1/94 | KR/96323/96 | HK/G9/97 | BJ/1/94 | BJ/1/94 |
| A/duck/Shantou/5401/2001 | B19 | SH/F/98 | Hok/49/98 | Hok/49/98 | BJ/1/94 | KR/96323/96 | HK/G9/97 | BJ/1/94 | BJ/1/94 |
| A/duck/Shantou/5459/2001 | B19 | SH/F/98 | Hok/49/98 | Hok/49/98 | BJ/1/94 | KR/96323/96 | HK/G9/97 | BJ/1/94 | BJ/1/94 |
| A/chicken/Shantou/5714/2001 | B19 | SH/F/98 | Hok/49/98 | Hok/49/98 | BJ/1/94 | KR/96323/96 | HK/G9/97 | BJ/1/94 | BJ/1/94 |
| A/duck/Shantou/5753/2001 | B19 | SH/F/98 | Hok/49/98 | Hok/49/98 | BJ/1/94 | KR/96323/96 | HK/G9/97 | BJ/1/94 | BJ/1/94 |
| A/chicken/Shantou/4/2002 | B19 | SH/F/98 | Hok/49/98 | Hok/49/98 | BJ/1/94 | KR/96323/96 | HK/G9/97 | BJ/1/94 | BJ/1/94 |
| A/duck/Shantou/32/2002 | B19 | SH/F/98 | Hok/49/98 | Hok/49/98 | BJ/1/94 | KR/96323/96 | HK/G9/97 | BJ/1/94 | BJ/1/94 |
| A/Partridge/Shantou/49/2002 | B19 | SH/F/98 | Hok/49/98 | Hok/49/98 | BJ/1/94 | KR/96323/96 | HK/G9/97 | BJ/1/94 | BJ/1/94 |
| A/chicken/Shantou/439/2002 | B19 | SH/F/98 | Hok/49/98 | Hok/49/98 | BJ/1/94 | KR/96323/96 | HK/G9/97 | BJ/1/94 | BJ/1/94 |
| A/quail/Shantou/786/2002 | B19 | SH/F/98 | Hok/49/98 | Hok/49/98 | BJ/1/94 | KR/96323/96 | HK/G9/97 | BJ/1/94 | BJ/1/94 |
| A/chicken/Shantou/2204/2002 | B19 | SH/F/98 | Hok/49/98 | Hok/49/98 | BJ/1/94 | KR/96323/96 | HK/G9/97 | BJ/1/94 | BJ/1/94 |
| A/duck/Shantou/4103/2002 | B19 | SH/F/98 | Hok/49/98 | Hok/49/98 | BJ/1/94 | KR/96323/96 | HK/G9/97 | BJ/1/94 | BJ/1/94 |
| A/chicken/Shantou/4144/2002 | B19 | SH/F/98 | Hok/49/98 | Hok/49/98 | BJ/1/94 | KR/96323/96 | HK/G9/97 | BJ/1/94 | BJ/1/94 |
| A/Partridge/Shantou/4525/2002 | B19 | SH/F/98 | Hok/49/98 | Hok/49/98 | BJ/1/94 | KR/96323/96 | HK/G9/97 | BJ/1/94 | BJ/1/94 |
| A/chicken/Shantou/4608/2002 | B19 | SH/F/98 | Hok/49/98 | Hok/49/98 | BJ/1/94 | KR/96323/96 | HK/G9/97 | BJ/1/94 | BJ/1/94 |
| A/chicken/Shantou/69/2003 | B19 | SH/F/98 | Hok/49/98 | Hok/49/98 | BJ/1/94 | KR/96323/96 | HK/G9/97 | BJ/1/94 | BJ/1/94 |
| A/chicken/Shantou/944/2003 | B19 | SH/F/98 | Hok/49/98 | Hok/49/98 | BJ/1/94 | KR/96323/96 | HK/G9/97 | BJ/1/94 | BJ/1/94 |
| A/chicken/Shantou/1689/2003 | B19 | SH/F/98 | Hok/49/98 | Hok/49/98 | BJ/1/94 | KR/96323/96 | HK/G9/97 | BJ/1/94 | BJ/1/94 |
| A/Partridge/Shantou/2803/2003 | B19 | SH/F/98 | Hok/49/98 | Hok/49/98 | BJ/1/94 | KR/96323/96 | HK/G9/97 | BJ/1/94 | BJ/1/94 |
| A/duck/Shantou/3460/2003 | B19 | SH/F/98 | Hok/49/98 | Hok/49/98 | BJ/1/94 | KR/96323/96 | HK/G9/97 | BJ/1/94 | BJ/1/94 |
| A/chicken/Shantou/7920/2004 | B19 | SH/F/98 | Hok/49/98 | Hok/49/98 | BJ/1/94 | KR/96323/96 | HK/G9/97 | BJ/1/94 | BJ/1/94 |
| A/Partridge/Shantou/7936/2004 | B19 | SH/F/98 | Hok/49/98 | Hok/49/98 | BJ/1/94 | KR/96323/96 | HK/G9/97 | BJ/1/94 | BJ/1/94 |
| A/chicken/Shantou/55/2005 | B19 | SH/F/98 | Hok/49/98 | Hok/49/98 | BJ/1/94 | KR/96323/96 | HK/G9/97 | BJ/1/94 | BJ/1/94 |
| A/pheasant/Shantou/111/2005 | B19 | SH/F/98 | Hok/49/98 | Hok/49/98 | BJ/1/94 | KR/96323/96 | HK/G9/97 | BJ/1/94 | BJ/1/94 |
| A/Partridge/Shantou/5692/2000***^a^*** | B20 | SH/F/98 | Hok/49/98 | Hok/49/98 | BJ/1/94 | KR/96323/96 | HK/G9/97 | HK/G1/97 | BJ/1/94 |
| A/pheasant/Shantou/511/2003 | B20 | SH/F/98 | Hok/49/98 | Hok/49/98 | BJ/1/94 | KR/96323/96 | HK/G9/97 | HK/G1/97 | BJ/1/94 |
| A/Partridge/Shantou/3987/2003 | B20 | SH/F/98 | Hok/49/98 | Hok/49/98 | BJ/1/94 | KR/96323/96 | HK/G9/97 | HK/G1/97 | BJ/1/94 |
| A/chicken/Guangxi/6/2000***^a^*** | B21 | SH/F/98 | HK/G1/97 | Hok/49/98 | BJ/1/94 | KR/96323/96 | HK/G9/97 | BJ/1/94 | BJ/1/94 |
| A/chicken/china/Guangxi14/2000***^a^*** | B22 | H5N1 | HK/G1/97 | H5N1 | BJ/1/94 | HK/G1/97 | HK/G9/97 | BJ/1/94 | BJ/1/94 |
| A/chicken/china/Guangxi17/2000***^a^*** | B23 | HK/G1/97 | HK/G1/97 | H5N1 | BJ/1/94 | HK/G1/97 | HK/G9/97 | BJ/1/94 | BJ/1/94 |
| A/Partridge/Shantou/24/2000***^a^*** | B24 | HK/G1/97 | HK/G1/97 | Hok/49/98 | BJ/1/94 | BJ/1/94 | HK/G9/97 | HK/G1/97 | BJ/1/94 |
| A/duck/Shantou/2134/2000 | B24 | HK/G1/97 | HK/G1/97 | Hok/49/98 | BJ/1/94 | BJ/1/94 | HK/G9/97 | HK/G1/97 | BJ/1/94 |
| A/Partridge/Shantou/2158/2000 | B24 | HK/G1/97 | HK/G1/97 | Hok/49/98 | BJ/1/94 | BJ/1/94 | HK/G9/97 | HK/G1/97 | BJ/1/94 |
| A/quail/Shantou/1425/2001 | B24 | HK/G1/97 | HK/G1/97 | Hok/49/98 | BJ/1/94 | BJ/1/94 | HK/G9/97 | HK/G1/97 | BJ/1/94 |
| A/quail/Shantou/1461/2001 | B24 | HK/G1/97 | HK/G1/97 | Hok/49/98 | BJ/1/94 | BJ/1/94 | HK/G9/97 | HK/G1/97 | BJ/1/94 |
| A/quail/Shantou/4179/2001 | B24 | HK/G1/97 | HK/G1/97 | Hok/49/98 | BJ/1/94 | BJ/1/94 | HK/G9/97 | HK/G1/97 | BJ/1/94 |
| A/Partridge/Shantou/2063/2000***^a^*** | B25 | HK/G1/97 | HK/G1/97 | HK/G1/97 | BJ/1/94 | BJ/1/94 | HK/G9/97 | HK/G1/97 | BJ/1/94 |
| A/Partridge/Shantou/1800/2000 | B25 | HK/G1/97 | HK/G1/97 | HK/G1/97 | BJ/1/94 | BJ/1/94 | HK/G9/97 | HK/G1/97 | BJ/1/94 |
| A/duck/Shantou/1042/2000 | B25 | HK/G1/97 | HK/G1/97 | HK/G1/97 | BJ/1/94 | BJ/1/94 | HK/G9/97 | HK/G1/97 | BJ/1/94 |
| A/chicken/Osaka/aq58/2001***^a^*** | B26 | SH/F/98 | Hok/49/98 | Hok/49/98 | BJ/1/94 | SH/F/98 | BJ/1/94 | BJ/1/94 | BJ/1/94 |
| A/chicken/Shantou/2686/2003 | B26 | SH/F/98 | Hok/49/98 | Hok/49/98 | BJ/1/94 | SH/F/98 | BJ/1/94 | BJ/1/94 | BJ/1/94 |
| A/chicken/Osaka/aq19/2001***^a^*** | B27 | SH/F/98 | BJ/1/94 | SH/F/98 | BJ/1/94 | SH/F/98 | HK/G9/97 | BJ/1/94 | BJ/1/94 |
| A/chicken/YoKohama/aq55/2001 | B27 | SH/F/98 | BJ/1/94 | SH/F/98 | BJ/1/94 | SH/F/98 | HK/G9/97 | BJ/1/94 | BJ/1/94 |
| A/chicken/YoKohama/aq135/2001 | B27 | SH/F/98 | BJ/1/94 | SH/F/98 | BJ/1/94 | SH/F/98 | HK/G9/97 | BJ/1/94 | BJ/1/94 |
| A/chicken/HK/TP38/03 | B27 | SH/F/98 | BJ/1/94 | SH/F/98 | BJ/1/94 | SH/F/98 | HK/G9/97 | BJ/1/94 | BJ/1/94 |
| A/CK/HK/YU463/2003 | B27 | SH/F/98 | BJ/1/94 | SH/F/98 | BJ/1/94 | SH/F/98 | HK/G9/97 | BJ/1/94 | BJ/1/94 |
| A/chicken/Shantou/1404/2003 | B27 | SH/F/98 | BJ/1/94 | SH/F/98 | BJ/1/94 | SH/F/98 | HK/G9/97 | BJ/1/94 | BJ/1/94 |
| A/chicken/Guangdong/56/01***^a^*** | B28 | SH/F/98 | SH/F/98 | SH/F/98 | BJ/1/94 | SH/F/98 | HK/G9/97 | BJ/1/94 | BJ/1/94 |
| A/chicken/Kobe/aq26/2001***^a^*** | B29 | SH/F/98 | SH/F/98 | SH/F/98 | BJ/1/94 | SH/F/98 | BJ/1/94 | BJ/1/94 | CA/189/66 |
| A/chicken/YoKohama/aq134/2002 | B29 | SH/F/98 | SH/F/98 | SH/F/98 | BJ/1/94 | SH/F/98 | BJ/1/94 | BJ/1/94 | CA/189/66 |
| A/chicken/Jiangsu/ng/2001***^a^*** | B30 | SH/F/98 | SH/F/98 | SH/F/98 | BJ/1/94 | SH/F/98 | BJ/1/94 | H5N1 | BJ/1/94 |
| A/swine/Shandong/nb/2003 | B30 | SH/F/98 | SH/F/98 | SH/F/98 | BJ/1/94 | SH/F/98 | BJ/1/94 | H5N1 | BJ/1/94 |
| A/quail/Shantou/5663/2001***^a^*** | B31 | HK/G1/97 | HK/G1/97 | ST/5663/01 | BJ/1/94 | BJ/1/94 | HK/G9/97 | HK/G1/97 | BJ/1/94 |
| A/Wild Duck/Shantou/4808/01 | B31 | HK/G1/97 | HK/G1/97 | ST/5663/01 | BJ/1/94 | BJ/1/94 | HK/G9/97 | HK/G1/97 | BJ/1/94 |
| A/Partridge/Shantou/4829/2001 | B31 | HK/G1/97 | HK/G1/97 | ST/5663/01 | BJ/1/94 | BJ/1/94 | HK/G9/97 | HK/G1/97 | BJ/1/94 |
| A/Partridge/Shantou/4541/2001 | B31 | HK/G1/97 | HK/G1/97 | ST/5663/01 | BJ/1/94 | BJ/1/94 | HK/G9/97 | HK/G1/97 | BJ/1/94 |
| A/quail/Shantou/5675/2001 | B31 | HK/G1/97 | HK/G1/97 | ST/5663/01 | BJ/1/94 | BJ/1/94 | HK/G9/97 | HK/G1/97 | BJ/1/94 |
| A/chukkar/Shantou/465/2002 | B31 | HK/G1/97 | HK/G1/97 | ST/5663/01 | BJ/1/94 | BJ/1/94 | HK/G9/97 | HK/G1/97 | BJ/1/94 |
| A/Partridge/Shantou/545/2002 | B31 | HK/G1/97 | HK/G1/97 | ST/5663/01 | BJ/1/94 | BJ/1/94 | HK/G9/97 | HK/G1/97 | BJ/1/94 |
| A/chukkar/Shantou/1059/2002 | B31 | HK/G1/97 | HK/G1/97 | ST/5663/01 | BJ/1/94 | BJ/1/94 | HK/G9/97 | HK/G1/97 | BJ/1/94 |
| A/Partridge/Shantou/3811/2002 | B31 | HK/G1/97 | HK/G1/97 | ST/5663/01 | BJ/1/94 | BJ/1/94 | HK/G9/97 | HK/G1/97 | BJ/1/94 |
| A/quail/Shantou/149/2003 | B31 | HK/G1/97 | HK/G1/97 | ST/5663/01 | BJ/1/94 | BJ/1/94 | HK/G9/97 | HK/G1/97 | BJ/1/94 |
| A/pheasant/Shantou/443/2003 | B31 | HK/G1/97 | HK/G1/97 | ST/5663/01 | BJ/1/94 | BJ/1/94 | HK/G9/97 | HK/G1/97 | BJ/1/94 |
| A/Partridge/Shantou/600/2003 | B31 | HK/G1/97 | HK/G1/97 | ST/5663/01 | BJ/1/94 | BJ/1/94 | HK/G9/97 | HK/G1/97 | BJ/1/94 |
| A/Gf/HK/SSP607/2003 | B31 | HK/G1/97 | HK/G1/97 | ST/5663/01 | BJ/1/94 | BJ/1/94 | HK/G9/97 | HK/G1/97 | BJ/1/94 |
| A/chukkar/Shantou/1039/2003 | B31 | HK/G1/97 | HK/G1/97 | ST/5663/01 | BJ/1/94 | BJ/1/94 | HK/G9/97 | HK/G1/97 | BJ/1/94 |
| A/quail/Shantou/1140/2003 | B31 | HK/G1/97 | HK/G1/97 | ST/5663/01 | BJ/1/94 | BJ/1/94 | HK/G9/97 | HK/G1/97 | BJ/1/94 |
| A/Ph/HK/CSW1323/2003 | B31 | HK/G1/97 | HK/G1/97 | ST/5663/01 | BJ/1/94 | BJ/1/94 | HK/G9/97 | HK/G1/97 | BJ/1/94 |
| A/quail/Shantou/1978/2003 | B31 | HK/G1/97 | HK/G1/97 | ST/5663/01 | BJ/1/94 | BJ/1/94 | HK/G9/97 | HK/G1/97 | BJ/1/94 |
| A/HK/2108/2003 | B31 | HK/G1/97 | HK/G1/97 | ST/5663/01 | BJ/1/94 | BJ/1/94 | HK/G9/97 | HK/G1/97 | BJ/1/94 |
| A/quail/Shantou/2608/2003 | B31 | HK/G1/97 | HK/G1/97 | ST/5663/01 | BJ/1/94 | BJ/1/94 | HK/G9/97 | HK/G1/97 | BJ/1/94 |
| A/pheasant/Shantou/3530/2003 | B31 | HK/G1/97 | HK/G1/97 | ST/5663/01 | BJ/1/94 | BJ/1/94 | HK/G9/97 | HK/G1/97 | BJ/1/94 |
| A/duck/Shantou/3658/2003 | B31 | HK/G1/97 | HK/G1/97 | ST/5663/01 | BJ/1/94 | BJ/1/94 | HK/G9/97 | HK/G1/97 | BJ/1/94 |
| A/quail/Shantou/3700/2003 | B31 | HK/G1/97 | HK/G1/97 | ST/5663/01 | BJ/1/94 | BJ/1/94 | HK/G9/97 | HK/G1/97 | BJ/1/94 |
| A/quail/Shantou/3856/2003 | B31 | HK/G1/97 | HK/G1/97 | ST/5663/01 | BJ/1/94 | BJ/1/94 | HK/G9/97 | HK/G1/97 | BJ/1/94 |
| A/chukkar/Shantou/3980/2003 | B31 | HK/G1/97 | HK/G1/97 | ST/5663/01 | BJ/1/94 | BJ/1/94 | HK/G9/97 | HK/G1/97 | BJ/1/94 |
| A/Partridge/Shantou/4093/2003 | B31 | HK/G1/97 | HK/G1/97 | ST/5663/01 | BJ/1/94 | BJ/1/94 | HK/G9/97 | HK/G1/97 | BJ/1/94 |
| A/pheasant/Shantou/4116/2003 | B31 | HK/G1/97 | HK/G1/97 | ST/5663/01 | BJ/1/94 | BJ/1/94 | HK/G9/97 | HK/G1/97 | BJ/1/94 |
| A/pheasant/Shantou/4469/2003 | B31 | HK/G1/97 | HK/G1/97 | ST/5663/01 | BJ/1/94 | BJ/1/94 | HK/G9/97 | HK/G1/97 | BJ/1/94 |
| A/pheasant/Shantou/4709/2003 | B31 | HK/G1/97 | HK/G1/97 | ST/5663/01 | BJ/1/94 | BJ/1/94 | HK/G9/97 | HK/G1/97 | BJ/1/94 |
| A/Partridge/Shantou/4889/2003 | B31 | HK/G1/97 | HK/G1/97 | ST/5663/01 | BJ/1/94 | BJ/1/94 | HK/G9/97 | HK/G1/97 | BJ/1/94 |
| A/Partridge/Shantou/25/2004 | B31 | HK/G1/97 | HK/G1/97 | ST/5663/01 | BJ/1/94 | BJ/1/94 | HK/G9/97 | HK/G1/97 | BJ/1/94 |
| A/silky chicken/Shantou/473/2004 | B31 | HK/G1/97 | HK/G1/97 | ST/5663/01 | BJ/1/94 | BJ/1/94 | HK/G9/97 | HK/G1/97 | BJ/1/94 |
| A/Partridge/Shantou/688/2004 | B31 | HK/G1/97 | HK/G1/97 | ST/5663/01 | BJ/1/94 | BJ/1/94 | HK/G9/97 | HK/G1/97 | BJ/1/94 |
| A/pheasant/Shantou/841/2004 | B31 | HK/G1/97 | HK/G1/97 | ST/5663/01 | BJ/1/94 | BJ/1/94 | HK/G9/97 | HK/G1/97 | BJ/1/94 |
| A/silky chicken/Shantou/999/2004 | B31 | HK/G1/97 | HK/G1/97 | ST/5663/01 | BJ/1/94 | BJ/1/94 | HK/G9/97 | HK/G1/97 | BJ/1/94 |
| A/Partridge/Shantou/1405/2004 | B31 | HK/G1/97 | HK/G1/97 | ST/5663/01 | BJ/1/94 | BJ/1/94 | HK/G9/97 | HK/G1/97 | BJ/1/94 |
| A/chukkar/Shantou/1447/2004 | B31 | HK/G1/97 | HK/G1/97 | ST/5663/01 | BJ/1/94 | BJ/1/94 | HK/G9/97 | HK/G1/97 | BJ/1/94 |
| A/Partridge/Shantou/1651/2004 | B31 | HK/G1/97 | HK/G1/97 | ST/5663/01 | BJ/1/94 | BJ/1/94 | HK/G9/97 | HK/G1/97 | BJ/1/94 |
| A/chukkar/Shantou/2226/2004 | B31 | HK/G1/97 | HK/G1/97 | ST/5663/01 | BJ/1/94 | BJ/1/94 | HK/G9/97 | HK/G1/97 | BJ/1/94 |
| A/pheasant/Shantou/2290/2004 | B31 | HK/G1/97 | HK/G1/97 | ST/5663/01 | BJ/1/94 | BJ/1/94 | HK/G9/97 | HK/G1/97 | BJ/1/94 |
| A/chicken/Shantou/2402/2004 | B31 | HK/G1/97 | HK/G1/97 | ST/5663/01 | BJ/1/94 | BJ/1/94 | HK/G9/97 | HK/G1/97 | BJ/1/94 |
| A/Partridge/Shantou/6266/2004 | B31 | HK/G1/97 | HK/G1/97 | ST/5663/01 | BJ/1/94 | BJ/1/94 | HK/G9/97 | HK/G1/97 | BJ/1/94 |
| A/chukkar/Shantou/6288/2004 | B31 | HK/G1/97 | HK/G1/97 | ST/5663/01 | BJ/1/94 | BJ/1/94 | HK/G9/97 | HK/G1/97 | BJ/1/94 |
| A/chukkar/Shantou/6571/2004 | B31 | HK/G1/97 | HK/G1/97 | ST/5663/01 | BJ/1/94 | BJ/1/94 | HK/G9/97 | HK/G1/97 | BJ/1/94 |
| A/chicken/Shantou/6786/2004 | B31 | HK/G1/97 | HK/G1/97 | ST/5663/01 | BJ/1/94 | BJ/1/94 | HK/G9/97 | HK/G1/97 | BJ/1/94 |
| A/pheasant/Shantou/6893/2004 | B31 | HK/G1/97 | HK/G1/97 | ST/5663/01 | BJ/1/94 | BJ/1/94 | HK/G9/97 | HK/G1/97 | BJ/1/94 |
| A/Partridge/Shantou/7343/2004 | B31 | HK/G1/97 | HK/G1/97 | ST/5663/01 | BJ/1/94 | BJ/1/94 | HK/G9/97 | HK/G1/97 | BJ/1/94 |
| A/pheasant/Shantou/7814/2004 | B31 | HK/G1/97 | HK/G1/97 | ST/5663/01 | BJ/1/94 | BJ/1/94 | HK/G9/97 | HK/G1/97 | BJ/1/94 |
| A/silky chicken/Shantou/459/2005 | B31 | HK/G1/97 | HK/G1/97 | ST/5663/01 | BJ/1/94 | BJ/1/94 | HK/G9/97 | HK/G1/97 | BJ/1/94 |
| A/Guinea fowl/Shantou/630/2005 | B31 | HK/G1/97 | HK/G1/97 | ST/5663/01 | BJ/1/94 | BJ/1/94 | HK/G9/97 | HK/G1/97 | BJ/1/94 |
| A/pheasant/Shantou/1578/2005 | B31 | HK/G1/97 | HK/G1/97 | ST/5663/01 | BJ/1/94 | BJ/1/94 | HK/G9/97 | HK/G1/97 | BJ/1/94 |
| A/quail/Shantou/4762/2001***^a^*** | B32 | HK/G1/97 | HK/G1/97 | HK/G1/97 | BJ/1/94 | HK/G1/97 | HK/G1/97 | HK/G1/97 | BJ/1/94 |
| A/chicken/Yunnan/401/2002***^a^*** | B33 | HK/G1/97 | HK/G1/97 | BJ/1/94 | BJ/1/94 | HK/G1/97 | HK/G9/97 | BJ/1/94 | BJ/1/94 |
| A/chicken/Yunnan/522/2002 | B33 | HK/G1/97 | HK/G1/97 | BJ/1/94 | BJ/1/94 | HK/G1/97 | HK/G9/97 | BJ/1/94 | BJ/1/94 |
| A/chicken/Yunnan/955/2002 | B33 | HK/G1/97 | HK/G1/97 | BJ/1/94 | BJ/1/94 | HK/G1/97 | HK/G9/97 | BJ/1/94 | BJ/1/94 |
| A/chicken/Yunnan/1147/2003 | B33 | HK/G1/97 | HK/G1/97 | BJ/1/94 | BJ/1/94 | HK/G1/97 | HK/G9/97 | BJ/1/94 | BJ/1/94 |
| A/chicken/Yunnan/5653/2003 | B33 | HK/G1/97 | HK/G1/97 | BJ/1/94 | BJ/1/94 | HK/G1/97 | HK/G9/97 | BJ/1/94 | BJ/1/94 |
| A/chicken/Yunnan/3511/2004 | B33 | HK/G1/97 | HK/G1/97 | BJ/1/94 | BJ/1/94 | HK/G1/97 | HK/G9/97 | BJ/1/94 | BJ/1/94 |
| A/chicken/Yunnan/3727/2004 | B33 | HK/G1/97 | HK/G1/97 | BJ/1/94 | BJ/1/94 | HK/G1/97 | HK/G9/97 | BJ/1/94 | BJ/1/94 |
| A/duck/Shantou/4359/2002***^a^*** | B34 | HK/G1/97 | HK/G1/97 | ST/5663/01 | BJ/1/94 | SH/F/98 | HK/G9/97 | BJ/1/94 | BJ/1/94 |
| A/CK/HK/NT142/2003 | B34 | HK/G1/97 | HK/G1/97 | ST/5663/01 | BJ/1/94 | SH/F/98 | HK/G9/97 | BJ/1/94 | BJ/1/94 |
| A/quail/Shantou/3502/2003 | B34 | HK/G1/97 | HK/G1/97 | ST/5663/01 | BJ/1/94 | SH/F/98 | HK/G9/97 | BJ/1/94 | BJ/1/94 |
| A/pheasant/Shantou/3716/2003 | B34 | HK/G1/97 | HK/G1/97 | ST/5663/01 | BJ/1/94 | SH/F/98 | HK/G9/97 | BJ/1/94 | BJ/1/94 |
| A/chicken/Shantou/1138/2004 | B34 | HK/G1/97 | HK/G1/97 | ST/5663/01 | BJ/1/94 | SH/F/98 | HK/G9/97 | BJ/1/94 | BJ/1/94 |
| A/chicken/Jiangsu/7/2002***^a^*** | B35 | SH/F/98 | SH/F/98 | SH/F/98 | BJ/1/94 | SH/F/98 | BJ/1/94 | BJ/1/94 | HK/Y439/97 |
| A/chicken/Neimenggu/nK/2002 | B35 | SH/F/98 | SH/F/98 | SH/F/98 | BJ/1/94 | SH/F/98 | BJ/1/94 | BJ/1/94 | HK/Y439/97 |
| A/chicken/Jiangsu/wa/2002 | B35 | SH/F/98 | SH/F/98 | SH/F/98 | BJ/1/94 | SH/F/98 | BJ/1/94 | BJ/1/94 | HK/Y439/97 |
| A/duck/Jiangsu/3/2005 | B35 | SH/F/98 | SH/F/98 | SH/F/98 | BJ/1/94 | SH/F/98 | BJ/1/94 | BJ/1/94 | HK/Y439/97 |
| A/chicken/Hubei/C1/2007 | B35 | SH/F/98 | SH/F/98 | SH/F/98 | BJ/1/94 | SH/F/98 | BJ/1/94 | BJ/1/94 | HK/Y439/97 |
| A/chukkar/Shantou/338/2002***^a^*** | B36 | HK/G1/97 | HK/G1/97 | ST/5663/01 | BJ/1/94 | SH/F/98 | HK/G9/97 | HK/G1/97 | BJ/1/94 |
| A/quail/Shantou/365/2002 | B36 | HK/G1/97 | HK/G1/97 | ST/5663/01 | BJ/1/94 | SH/F/98 | HK/G9/97 | HK/G1/97 | BJ/1/94 |
| A/quail/Shantou/384/2002 | B36 | HK/G1/97 | HK/G1/97 | ST/5663/01 | BJ/1/94 | SH/F/98 | HK/G9/97 | HK/G1/97 | BJ/1/94 |
| A/quail/Shantou/396/2002 | B36 | HK/G1/97 | HK/G1/97 | ST/5663/01 | BJ/1/94 | SH/F/98 | HK/G9/97 | HK/G1/97 | BJ/1/94 |
| A/pheasant/Shantou/7501/2004 | B36 | HK/G1/97 | HK/G1/97 | ST/5663/01 | BJ/1/94 | SH/F/98 | HK/G9/97 | HK/G1/97 | BJ/1/94 |
| A/quail/Shantou/3143/2005 | B36 | HK/G1/97 | HK/G1/97 | ST/5663/01 | BJ/1/94 | SH/F/98 | HK/G9/97 | HK/G1/97 | BJ/1/94 |
| A/quail/Shantou/5011/2005 | B36 | HK/G1/97 | HK/G1/97 | ST/5663/01 | BJ/1/94 | SH/F/98 | HK/G9/97 | HK/G1/97 | BJ/1/94 |
| A/quail/Shantou/8993/2005 | B36 | HK/G1/97 | HK/G1/97 | ST/5663/01 | BJ/1/94 | SH/F/98 | HK/G9/97 | HK/G1/97 | BJ/1/94 |
| A/quail/Shantou/1038/2002***^a^*** | B37 | H5N1 | HK/G1/97 | ST/5663/01 | BJ/1/94 | SH/F/98 | HK/G9/97 | HK/G1/97 | BJ/1/94 |
| A/quail/Shantou/2615/2003***^a^*** | B38 | HK/G1/97 | HK/G1/97 | ST/5663/01 | BJ/1/94 | BJ/1/94 | HK/G9/97 | BJ/1/94 | BJ/1/94 |
| A/chicken/Shantou/3040/2003***^a^*** | B39 | SH/F/98 | SH/F/98 | ST/5663/01 | BJ/1/94 | SH/F/98 | HK/G9/97 | BJ/1/94 | BJ/1/94 |
| A/chicken/Shantou/3341/2003 | B39 | SH/F/98 | SH/F/98 | ST/5663/01 | BJ/1/94 | SH/F/98 | HK/G9/97 | BJ/1/94 | BJ/1/94 |
| A/Partridge/Shantou/3487/2003 | B39 | SH/F/98 | SH/F/98 | ST/5663/01 | BJ/1/94 | SH/F/98 | HK/G9/97 | BJ/1/94 | BJ/1/94 |
| A/duck/Shantou/3577/2003 | B39 | SH/F/98 | SH/F/98 | ST/5663/01 | BJ/1/94 | SH/F/98 | HK/G9/97 | BJ/1/94 | BJ/1/94 |
| A/duck/Shantou/3728/2003 | B39 | SH/F/98 | SH/F/98 | ST/5663/01 | BJ/1/94 | SH/F/98 | HK/G9/97 | BJ/1/94 | BJ/1/94 |
| A/partridge/Shantou/4321/2003 | B39 | SH/F/98 | SH/F/98 | ST/5663/01 | BJ/1/94 | SH/F/98 | HK/G9/97 | BJ/1/94 | BJ/1/94 |
| A/pheasant/Shantou/4340/2003 | B39 | SH/F/98 | SH/F/98 | ST/5663/01 | BJ/1/94 | SH/F/98 | HK/G9/97 | BJ/1/94 | BJ/1/94 |
| A/chicken/Shantou/4435/2003 | B39 | SH/F/98 | SH/F/98 | ST/5663/01 | BJ/1/94 | SH/F/98 | HK/G9/97 | BJ/1/94 | BJ/1/94 |
| A/silky chicken/4600/2003 | B39 | SH/F/98 | SH/F/98 | ST/5663/01 | BJ/1/94 | SH/F/98 | HK/G9/97 | BJ/1/94 | BJ/1/94 |
| A/chicken/Shantou/4617/2003 | B39 | SH/F/98 | SH/F/98 | ST/5663/01 | BJ/1/94 | SH/F/98 | HK/G9/97 | BJ/1/94 | BJ/1/94 |
| A/chukkar/Shantou/4635/2003 | B39 | SH/F/98 | SH/F/98 | ST/5663/01 | BJ/1/94 | SH/F/98 | HK/G9/97 | BJ/1/94 | BJ/1/94 |
| A/chicken/Shantou/99/2004 | B39 | SH/F/98 | SH/F/98 | ST/5663/01 | BJ/1/94 | SH/F/98 | HK/G9/97 | BJ/1/94 | BJ/1/94 |
| A/duck/Shantou/515/2004 | B39 | SH/F/98 | SH/F/98 | ST/5663/01 | BJ/1/94 | SH/F/98 | HK/G9/97 | BJ/1/94 | BJ/1/94 |
| A/chicken/Shantou/4101/2003***^a^*** | B40 | SH/F/98 | SH/F/98 | ST/5663/01 | BJ/1/94 | KR/96323/96 | HK/G9/97 | BJ/1/94 | BJ/1/94 |
| A/quail/Shantou/4038/2003***^a^*** | B41 | HK/G1/97 | HK/G1/97 | ST/5663/01 | BJ/1/94 | HK/G1/97 | HK/G9/97 | BJ/1/94 | BJ/1/94 |
| A/guineafowl/HongKong/NT184/03***^a^*** | B42 | HK/G1/97 | HK/G1/97 | HK/Y439/97 | BJ/1/94 | BJ/1/94 | HK/G9/97 | HK/G1/97 | BJ/1/94 |
| A/chukkar/Shantou/4350/2003 | B42 | HK/G1/97 | HK/G1/97 | HK/Y439/97 | BJ/1/94 | BJ/1/94 | HK/G9/97 | HK/G1/97 | BJ/1/94 |
| A/quail/Shantou/1780/2003***^a^*** | B43 | SH/F/98 | Hok/49/98 | ST/5663/01 | BJ94 | KR/96323/96 | HK/G9/97 | HK/G1/97 | BJ/1/94 |
| A/swine/Guangdong/WXI/2004***^a^*** | B44 | BJ/1/94 | BJ/1/94 | WI/1/66 | BJ/1/94 | BJ/1/94 | BJ/1/94 | HK/289/78 | BJ/1/94 |
| A/chicken/Shantou/4726/2004***^a^*** | B45 | SH/F/98 | BJ/1/94 | ST/5663/01 | BJ/1/94 | SH/F/98 | HK/G9/97 | BJ/1/94 | BJ/1/94 |
| A/pheasant/Shantou/5588/2004 | B45 | SH/F/98 | BJ/1/94 | ST/5663/01 | BJ/1/94 | SH/F/98 | HK/G9/97 | BJ/1/94 | BJ/1/94 |
| A/chicken/Shantou/5630/2004 | B45 | SH/F/98 | BJ/1/94 | ST/5663/01 | BJ/1/94 | SH/F/98 | HK/G9/97 | BJ/1/94 | BJ/1/94 |
| A/Guinea fowl/Shantou/5852/2004 | B45 | SH/F/98 | BJ/1/94 | ST/5663/01 | BJ/1/94 | SH/F/98 | HK/G9/97 | BJ/1/94 | BJ/1/94 |
| A/chukkar/Shantou/5866/2004 | B45 | SH/F/98 | BJ/1/94 | ST/5663/01 | BJ/1/94 | SH/F/98 | HK/G9/97 | BJ/1/94 | BJ/1/94 |
| A/silky chicken/Shantou/6020/2004 | B45 | SH/F/98 | BJ/1/94 | ST/5663/01 | BJ/1/94 | SH/F/98 | HK/G9/97 | BJ/1/94 | BJ/1/94 |
| A/quail/Shantou/6046/2004 | B45 | SH/F/98 | BJ/1/94 | ST/5663/01 | BJ/1/94 | SH/F/98 | HK/G9/97 | BJ/1/94 | BJ/1/94 |
| A/chicken/Shantou/6319/2004 | B45 | SH/F/98 | BJ/1/94 | ST/5663/01 | BJ/1/94 | SH/F/98 | HK/G9/97 | BJ/1/94 | BJ/1/94 |
| A/Partridge/Shantou/6415/2004 | B45 | SH/F/98 | BJ/1/94 | ST/5663/01 | BJ/1/94 | SH/F/98 | HK/G9/97 | BJ/1/94 | BJ/1/94 |
| A/pheasant/Shantou/6607/2004 | B45 | SH/F/98 | BJ/1/94 | ST/5663/01 | BJ/1/94 | SH/F/98 | HK/G9/97 | BJ/1/94 | BJ/1/94 |
| A/quail/Shantou/6648/2004 | B45 | SH/F/98 | BJ/1/94 | ST/5663/01 | BJ/1/94 | SH/F/98 | HK/G9/97 | BJ/1/94 | BJ/1/94 |
| A/chicken/Shantou/6911/2004 | B45 | SH/F/98 | BJ/1/94 | ST/5663/01 | BJ/1/94 | SH/F/98 | HK/G9/97 | BJ/1/94 | BJ/1/94 |
| A/Partridge/Shantou/7075/2004 | B45 | SH/F/98 | BJ/1/94 | ST/5663/01 | BJ/1/94 | SH/F/98 | HK/G9/97 | BJ/1/94 | BJ/1/94 |
| A/pheasant/Shantou/7315/2004 | B45 | SH/F/98 | BJ/1/94 | ST/5663/01 | BJ/1/94 | SH/F/98 | HK/G9/97 | BJ/1/94 | BJ/1/94 |
| A/quail/Shantou/299/2005 | B45 | SH/F/98 | BJ/1/94 | ST/5663/01 | BJ/1/94 | SH/F/98 | HK/G9/97 | BJ/1/94 | BJ/1/94 |
| A/silky chicken/Shantou/1169/2005 | B45 | SH/F/98 | BJ/1/94 | ST/5663/01 | BJ/1/94 | SH/F/98 | HK/G9/97 | BJ/1/94 | BJ/1/94 |
| A/silky chicken/Shantou/2131/2005 | B45 | SH/F/98 | BJ/1/94 | ST/5663/01 | BJ/1/94 | SH/F/98 | HK/G9/97 | BJ/1/94 | BJ/1/94 |
| A/chicken/Fujian/3080/2005 | B45 | SH/F/98 | BJ/1/94 | ST/5663/01 | BJ/1/94 | SH/F/98 | HK/G9/97 | BJ/1/94 | BJ/1/94 |
| A/chicken/Fujian/4332/2005 | B45 | SH/F/98 | BJ/1/94 | ST/5663/01 | BJ/1/94 | SH/F/98 | HK/G9/97 | BJ/1/94 | BJ/1/94 |
| A/chicken/Fujian/4820/2005 | B45 | SH/F/98 | BJ/1/94 | ST/5663/01 | BJ/1/94 | SH/F/98 | HK/G9/97 | BJ/1/94 | BJ/1/94 |
| A/chicken/Fujian/5214/2005 | B45 | SH/F/98 | BJ/1/94 | ST/5663/01 | BJ/1/94 | SH/F/98 | HK/G9/97 | BJ/1/94 | BJ/1/94 |
| A/chicken/Shantou/5269/2005 | B45 | SH/F/98 | BJ/1/94 | ST/5663/01 | BJ/1/94 | SH/F/98 | HK/G9/97 | BJ/1/94 | BJ/1/94 |
| A/chicken/Fujian/5683/2005 | B45 | SH/F/98 | BJ/1/94 | ST/5663/01 | BJ/1/94 | SH/F/98 | HK/G9/97 | BJ/1/94 | BJ/1/94 |
| A/chicken/Fujian/7386/2005 | B45 | SH/F/98 | BJ/1/94 | ST/5663/01 | BJ/1/94 | SH/F/98 | HK/G9/97 | BJ/1/94 | BJ/1/94 |
| A/chicken/Fujian/7884/2005 | B45 | SH/F/98 | BJ/1/94 | ST/5663/01 | BJ/1/94 | SH/F/98 | HK/G9/97 | BJ/1/94 | BJ/1/94 |
| A/chicken/Fujian/6188/2005 | B45 | SH/F/98 | BJ/1/94 | ST/5663/01 | BJ/1/94 | SH/F/98 | HK/G9/97 | BJ/1/94 | BJ/1/94 |
| A/chicken/Shantou/6781/2005 | B45 | SH/F/98 | BJ/1/94 | ST/5663/01 | BJ/1/94 | SH/F/98 | HK/G9/97 | BJ/1/94 | BJ/1/94 |
| A/chicken/Fujian/6960/2005 | B45 | SH/F/98 | BJ/1/94 | ST/5663/01 | BJ/1/94 | SH/F/98 | HK/G9/97 | BJ/1/94 | BJ/1/94 |
| A/Guinea fowl/Shantou/8955/2005 | B45 | SH/F/98 | BJ/1/94 | ST/5663/01 | BJ/1/94 | SH/F/98 | HK/G9/97 | BJ/1/94 | BJ/1/94 |
| A/chicken/Shantou/22504/2005 | B45 | SH/F/98 | BJ/1/94 | ST/5663/01 | BJ/1/94 | SH/F/98 | HK/G9/97 | BJ/1/94 | BJ/1/94 |
| A/chicken/Guangxi/1857/2004***^a^*** | B46 | SH/F/98 | H5N1 | SH/F/98 | BJ/1/94 | SH/F/98 | BJ/1/94 | BJ/1/94 | BJ/1/94 |
| A/chicken/Guangxi/2441/2004 | B46 | SH/F/98 | H5N1 | SH/F/98 | BJ/1/94 | SH/F/98 | BJ/1/94 | BJ/1/94 | BJ/1/94 |
| A/chicken/Guangxi/37/2005 | B46 | SH/F/98 | H5N1 | SH/F/98 | BJ/1/94 | SH/F/98 | BJ/1/94 | BJ/1/94 | BJ/1/94 |
| A/duck/Guangxi/51/2005 | B46 | SH/F/98 | H5N1 | SH/F/98 | BJ/1/94 | SH/F/98 | BJ/1/94 | BJ/1/94 | BJ/1/94 |
| A/bird/Guangxi/62/2005 | B46 | SH/F/98 | H5N1 | SH/F/98 | BJ/1/94 | SH/F/98 | BJ/1/94 | BJ/1/94 | BJ/1/94 |
| A/bird/Guangxi/82/2005 | B46 | SH/F/98 | H5N1 | SH/F/98 | BJ/1/94 | SH/F/98 | BJ/1/94 | BJ/1/94 | BJ/1/94 |
| A/bird/Guangxi/83/2005 | B46 | SH/F/98 | H5N1 | SH/F/98 | BJ/1/94 | SH/F/98 | BJ/1/94 | BJ/1/94 | BJ/1/94 |
| A/chicken/Guangxi/187/2005 | B46 | SH/F/98 | H5N1 | SH/F/98 | BJ/1/94 | SH/F/98 | BJ/1/94 | BJ/1/94 | BJ/1/94 |
| A/swine/Guangxi/S11/2005 | B46 | SH/F/98 | H5N1 | SH/F/98 | BJ/1/94 | SH/F/98 | BJ/1/94 | BJ/1/94 | BJ/1/94 |
| A/quail/Guangxi/B1/2006 | B46 | SH/F/98 | H5N1 | SH/F/98 | BJ/1/94 | SH/F/98 | BJ/1/94 | BJ/1/94 | BJ/1/94 |
| A/bird/Guangxi/H1/2006 | B46 | SH/F/98 | H5N1 | SH/F/98 | BJ/1/94 | SH/F/98 | BJ/1/94 | BJ/1/94 | BJ/1/94 |
| A/Partridge/Shantou/4648/2004***^a^*** | B47 | SH/F/98 | BJ/1/94 | ST/5663/01 | BJ/1/94 | SH/F/98 | HK/G9/97 | HK/G1/97 | BJ/1/94 |
| A/Partridge/Shantou/6004/2004 | B47 | SH/F/98 | BJ/1/94 | ST/5663/01 | BJ/1/94 | SH/F/98 | HK/G9/97 | HK/G1/97 | BJ/1/94 |
| A/quail/Shantou/6794/2004 | B47 | SH/F/98 | BJ/1/94 | ST/5663/01 | BJ/1/94 | SH/F/98 | HK/G9/97 | HK/G1/97 | BJ/1/94 |
| A/quail/Shantou/7397/2004 | B47 | SH/F/98 | BJ/1/94 | ST/5663/01 | BJ/1/94 | SH/F/98 | HK/G9/97 | HK/G1/97 | BJ/1/94 |
| A/quail/Shantou/7731/2004 | B47 | SH/F/98 | BJ/1/94 | ST/5663/01 | BJ/1/94 | SH/F/98 | HK/G9/97 | HK/G1/97 | BJ/1/94 |
| A/silky chicken/Shantou/7790/2004 | B47 | SH/F/98 | BJ/1/94 | ST/5663/01 | BJ/1/94 | SH/F/98 | HK/G9/97 | HK/G1/97 | BJ/1/94 |
| A/quail/Shantou/493/2005 | B47 | SH/F/98 | BJ/1/94 | ST/5663/01 | BJ/1/94 | SH/F/98 | HK/G9/97 | HK/G1/97 | BJ/1/94 |
| A/quail/Shantou/2200/2005 | B47 | SH/F/98 | BJ/1/94 | ST/5663/01 | BJ/1/94 | SH/F/98 | HK/G9/97 | HK/G1/97 | BJ/1/94 |
| A/chicken/Fujian/8341/2005 | B47 | SH/F/98 | BJ/1/94 | ST/5663/01 | BJ/1/94 | SH/F/98 | HK/G9/97 | HK/G1/97 | BJ/1/94 |
| A/chicken/Fujian/9104/2005 | B47 | SH/F/98 | BJ/1/94 | ST/5663/01 | BJ/1/94 | SH/F/98 | HK/G9/97 | HK/G1/97 | BJ/1/94 |
| A/chicken/Fujian/9290/2005 | B47 | SH/F/98 | BJ/1/94 | ST/5663/01 | BJ/1/94 | SH/F/98 | HK/G9/97 | HK/G1/97 | BJ/1/94 |
| A/chicken/Fujian/9752/2005 | B47 | SH/F/98 | BJ/1/94 | ST/5663/01 | BJ/1/94 | SH/F/98 | HK/G9/97 | HK/G1/97 | BJ/1/94 |
| A/chicken/Shantou/9909/2005 | B47 | SH/F/98 | BJ/1/94 | ST/5663/01 | BJ/1/94 | SH/F/98 | HK/G9/97 | HK/G1/97 | BJ/1/94 |
| A/chicken/Fujian/10308/2005 | B47 | SH/F/98 | BJ/1/94 | ST/5663/01 | BJ/1/94 | SH/F/98 | HK/G9/97 | HK/G1/97 | BJ/1/94 |
| A/chicken/Fujian/10954/2005 | B47 | SH/F/98 | BJ/1/94 | ST/5663/01 | BJ/1/94 | SH/F/98 | HK/G9/97 | HK/G1/97 | BJ/1/94 |
| A/chicken/Fujian/11302/2005 | B47 | SH/F/98 | BJ/1/94 | ST/5663/01 | BJ/1/94 | SH/F/98 | HK/G9/97 | HK/G1/97 | BJ/1/94 |
| A/chicken/Fujian/11488/2005 | B47 | SH/F/98 | BJ/1/94 | ST/5663/01 | BJ/1/94 | SH/F/98 | HK/G9/97 | HK/G1/97 | BJ/1/94 |
| A/pheasant/Shantou/11551/2005 | B47 | SH/F/98 | BJ/1/94 | ST/5663/01 | BJ/1/94 | SH/F/98 | HK/G9/97 | HK/G1/97 | BJ/1/94 |
| A/chicken/Fujian/12252/2005 | B47 | SH/F/98 | BJ/1/94 | ST/5663/01 | BJ/1/94 | SH/F/98 | HK/G9/97 | HK/G1/97 | BJ/1/94 |
| A/duck/Shantou/12560/2005 | B47 | SH/F/98 | BJ/1/94 | ST/5663/01 | BJ/1/94 | SH/F/98 | HK/G9/97 | HK/G1/97 | BJ/1/94 |
| A/chicken/Shantou/14907/2005 | B47 | SH/F/98 | BJ/1/94 | ST/5663/01 | BJ/1/94 | SH/F/98 | HK/G9/97 | HK/G1/97 | BJ/1/94 |
| A/chicken/Shantou/17139/2005 | B47 | SH/F/98 | BJ/1/94 | ST/5663/01 | BJ/1/94 | SH/F/98 | HK/G9/97 | HK/G1/97 | BJ/1/94 |
| A/chicken/Shantou/20817/2005 | B47 | SH/F/98 | BJ/1/94 | ST/5663/01 | BJ/1/94 | SH/F/98 | HK/G9/97 | HK/G1/97 | BJ/1/94 |
| A/chicken/Shantou/22054/2005 | B47 | SH/F/98 | BJ/1/94 | ST/5663/01 | BJ/1/94 | SH/F/98 | HK/G9/97 | HK/G1/97 | BJ/1/94 |
| A/chicken/Henan/L3/2008 | B47 | SH/F/98 | BJ/1/94 | ST/5663/01 | BJ/1/94 | SH/F/98 | HK/G9/97 | HK/G1/97 | BJ/1/94 |
| A/pheasant/Shantou/45/2004***^a^*** | B48 | H5N1 | HK/G1/97 | ST/5663/01 | BJ/1/94 | SH/F/98 | HK/G9/97 | BJ/1/94 | BJ/1/94 |
| A/chicken/Henan/01/2004***^a^*** | B49 | H5N1 | H5N1 | H5N1 | BJ/1/94 | SH/F/98 | BJ/1/94 | H5N1 | BJ/1/94 |
| A/swine/Guangxi/FS2/2005***^a^*** | B50 | SH/F/98 | HK/G1/97 | SH/F/98 | BJ/1/94 | SH/F/98 | BJ/1/94 | BJ/1/94 | BJ/1/94 |
| A/swine/Guangxi/S15/2005***^a^*** | B51 | HK/G1/97 | H5N1 | SH/F/98 | BJ/1/94 | SH/F/98 | BJ/1/94 | BJ/1/94 | BJ/1/94 |
| A/chicken/Guangxi/521/2005***^a^*** | B52 | HK/289/78 | H5N1 | SH/F/98 | BJ/1/94 | SH/F/98 | BJ/1/94 | BJ/1/94 | BJ/1/94 |
| A/swine/Guangxi/58/2005***^a^*** | B53 | H5N1 | H5N1 | SH/F/98 | BJ/1/94 | SH/F/98 | BJ/1/94 | BJ/1/94 | BJ/1/94 |
| A/chicken/Guangxi/1428/2005***^a^*** | B54 | SH/F/98 | BJ/1/94 | ST/5663/01 | BJ/1/94 | SH/F/98 | BJ/1/94 | HK/G1/97 | BJ/1/94 |
| A/chicken/Guangxi/1032/2005 | B54 | SH/F/98 | BJ/1/94 | ST/5663/01 | BJ/1/94 | SH/F/98 | BJ/1/94 | HK/G1/97 | BJ/1/94 |
| A/chicken/Hunan/2536/2005 | B54 | SH/F/98 | BJ/1/94 | ST/5663/01 | BJ/1/94 | SH/F/98 | BJ/1/94 | HK/G1/97 | BJ/1/94 |
| A/chicken/Hunan/2903/2005 | B54 | SH/F/98 | BJ/1/94 | ST/5663/01 | BJ/1/94 | SH/F/98 | BJ/1/94 | HK/G1/97 | BJ/1/94 |
| A/chicken/Hunan/3369/2005 | B54 | SH/F/98 | BJ/1/94 | ST/5663/01 | BJ/1/94 | SH/F/98 | BJ/1/94 | HK/G1/97 | BJ/1/94 |
| A/chicken/Guangxi/4745/2005 | B54 | SH/F/98 | BJ/1/94 | ST/5663/01 | BJ/1/94 | SH/F/98 | BJ/1/94 | HK/G1/97 | BJ/1/94 |
| A/chicken/Hunan/5700/2005 | B54 | SH/F/98 | BJ/1/94 | ST/5663/01 | BJ/1/94 | SH/F/98 | BJ/1/94 | HK/G1/97 | BJ/1/94 |
| A/chicken/Hunan/6108/2005***^a^*** | B55 | SH/F/98 | SH/F/98 | SH/F/98 | BJ/1/94 | SH/F/98 | BJ/1/94 | HK/G1/97 | BJ/1/94 |
| A/chicken/Hunan/4444/2005 | B55 | SH/F/98 | SH/F/98 | SH/F/98 | BJ/1/94 | SH/F/98 | BJ/1/94 | HK/G1/97 | BJ/1/94 |
| A/chicken/Hunan/5260/2005 | B55 | SH/F/98 | SH/F/98 | SH/F/98 | BJ/1/94 | SH/F/98 | BJ/1/94 | HK/G1/97 | BJ/1/94 |
| A/chicken/Shandong/B4/2007 | B55 | SH/F/98 | SH/F/98 | SH/F/98 | BJ/1/94 | SH/F/98 | BJ/1/94 | HK/G1/97 | BJ/1/94 |
| A/chicken/Shanghai/Y2/2007 | B55 | SH/F/98 | SH/F/98 | SH/F/98 | BJ/1/94 | SH/F/98 | BJ/1/94 | HK/G1/97 | BJ/1/94 |
| A/chicken/Guangxi/55/2005***^a^*** | B56 | HK/289/78 | ？ | ST/5663/01 | BJ/1/94 | SH/F/98 | BJ/1/94 | HK/G1/97 | BJ/1/94 |
| A/chicken/Shantou/19465/2005***^a^*** | B57 | SH/F/98 | Hok/49/98 | ST/5663/01 | BJ/1/94 | SH/F/98 | HK/G9/97 | HK/G1/97 | BJ/1/94 |
| A/chicken/Hunan/4246/2005***^a^*** | B58 | HK/G1/97 | HK/G1/97 | ST/5663/01 | BJ/1/94 | HK/G1/97 | HK/G9/97 | HK/G1/97 | BJ/1/94 |
| A/quail/Shantou/15892/2005 | B58 | HK/G1/97 | HK/G1/97 | ST/5663/01 | BJ/1/94 | HK/G1/97 | HK/G9/97 | HK/G1/97 | BJ/1/94 |
| A/pheasant/Shantou/17033/2005 | B58 | HK/G1/97 | HK/G1/97 | ST/5663/01 | BJ/1/94 | HK/G1/97 | HK/G9/97 | HK/G1/97 | BJ/1/94 |
| A/quail/Shantou/20787/2005 | B58 | HK/G1/97 | HK/G1/97 | ST/5663/01 | BJ/1/94 | HK/G1/97 | HK/G9/97 | HK/G1/97 | BJ/1/94 |
| A/pheasant/Shantou/21583/2005 | B58 | HK/G1/97 | HK/G1/97 | ST/5663/01 | BJ/1/94 | HK/G1/97 | HK/G9/97 | HK/G1/97 | BJ/1/94 |
| A/Partridge/Shantou/22102/2005 | B58 | HK/G1/97 | HK/G1/97 | ST/5663/01 | BJ/1/94 | HK/G1/97 | HK/G9/97 | HK/G1/97 | BJ/1/94 |
| A/bird/Guangxi/A1/2006***^a^*** | B59 | HK/G1/97 | HK/G1/97 | BJ/1/94 | BJ/1/94 | SH/F/98 | HK/G9/97 | BJ/1/94 | BJ/1/94 |
| A/chicken/Shandong/B2/2007***^a^*** | B60 | BJ/1/94 | SH/F/98 | SH/F/98 | BJ/1/94 | SH/F/98 | BJ/1/94 | BJ/1/94 | BJ/1/94 |
| A/chicken/Zhejiang/Hj/2007***^a^*** | B61 | ST/163/04 | SH/F/98 | SH/F/98 | BJ/1/94 | SH/F/98 | BJ/1/94 | HK/G1/97 | BJ/1/94 |
| A/chicken/Korea/38349-p96323/96***^a^*** | C0***^b^*** | KR/96323/96 | KR/96323/96 | KR/96323/96 | KR/96323/96 | KR/96323/96 | KR/96323/96 | KR/96323/96 | KR/96323/96 |
| A/chicken/Korea/99029/99 | C0 | KR/96323/96 | KR/96323/96 | KR/96323/96 | KR/96323/96 | KR/96323/96 | KR/96323/96 | KR/96323/96 | KR/96323/96 |
| A/chicken/Korea/S1/2003 | C0 | KR/96323/96 | KR/96323/96 | KR/96323/96 | KR/96323/96 | KR/96323/96 | KR/96323/96 | KR/96323/96 | KR/96323/96 |
| A/silky chicken/Korea/S3/03 | C0 | KR/96323/96 | KR/96323/96 | KR/96323/96 | KR/96323/96 | KR/96323/96 | KR/96323/96 | KR/96323/96 | KR/96323/96 |
| A/chicken/Korea/S5/2003 | C0 | KR/96323/96 | KR/96323/96 | KR/96323/96 | KR/96323/96 | KR/96323/96 | KR/96323/96 | KR/96323/96 | KR/96323/96 |
| A/duck/Korea/S13/03 | C0 | KR/96323/96 | KR/96323/96 | KR/96323/96 | KR/96323/96 | KR/96323/96 | KR/96323/96 | KR/96323/96 | KR/96323/96 |
| A/chicken/Korea/S21/2004 | C0 | KR/96323/96 | KR/96323/96 | KR/96323/96 | KR/96323/96 | KR/96323/96 | KR/96323/96 | KR/96323/96 | KR/96323/96 |
| A/swine/Korea/S452/2004 | C0 | KR/96323/96 | KR/96323/96 | KR/96323/96 | KR/96323/96 | KR/96323/96 | KR/96323/96 | KR/96323/96 | KR/96323/96 |
| A/Korea/KBNP-0028/2000***^a^*** | C1 | DE/113/95 | KR/96323/96 | KR/96323/96 | KR/96323/96 | KR/96323/96 | KR/96323/96 | KR/96323/96 | KR/96323/96 |
| A/chicken/Korea/S16/03***^a^*** | C2 | KR/96323/96 | KR/96323/96 | KR/96323/96 | KR/96323/96 | KR/96323/96 | KR/96323/96 | BJ/1/94 | KR/96323/96 |
| A/swine/Korea/S190/2004***^a^*** | C3 | KR/96323/96 | WI/1/66 | WI/1/66 | KR/96323/96 | WI/1/66 | KR/96323/96 | WI/1/66 | KR/96323/96 |
| A/Duck/Germany/113/95***^a^*** | D1 | DE/113/95 | DE/113/95 | DE/113/95 | DE/113/95 | DE/113/95 | DE/113/95 | DE/113/95 | HK/289/78 |
| A/ostrich/South Africa/9508103/95***^a^*** | D2 | HK/289/78 | Hok/49/98 | KR/96323/96 | DE/113/95 | HK/Y439/97 | KR/96323/96 | DE/113/95 | HK/Y439/97 |
| A/pheasant/Ireland/PV18/97***^a^*** | D3 | DE/113/95 | HK/AF157/92 | DE/113/95 | DE/113/95 | HK/Y439/97 | KR/96323/96 | DE/113/95 | CA/189/66 |
| A/duck/Hongkong/Y439/97***^a^*** | D4 | HK/289/78 | HK/289/78 | HK/Y439/97 | DE/113/95 | HK/Y439/97 | HK/Y439/97 | DE/113/95 | HK/Y439/97 |
| A/duck/Hokkaido/49/98***^a^*** | D5 | HK/289/78 | Hok/49/98 | Hok/49/98 | DE/113/95 | HK/Y439/97 | KR/96323/96 | DE/113/95 | HK/Y439/97 |
| A/duck/Hokkaido/9/99***^a^*** | D6 | SH/F/98 | KR/96323/96 | KR/96323/96 | DE/113/95 | HK/Y439/97 | KR/96323/96 | DE/113/95 | HK/Y439/97 |
| A/duck/Shantou/163/2004***^a^*** | D7 | ST/163/04 | DE/113/95 | KR/96323/96 | DE/113/95 | HK/Y439/97 | KR/96323/96 | DE/113/95 | HK/Y439/97 |
| A/duck/Shantou/7488/2004***^a^*** | D8 | ST/163/04 | H5N1 | SH/F/98 | DE/113/95 | HK/Y439/97 | KR/96323/96 | DE/113/95 | HK/Y439/97 |
| A/Eurasian wigeon/Netherlands/3/2005***^a^*** | D9 | HK/289/78 | DE/113/95 | KR/96323/96 | DE/113/95 | HK/Y439/97 | KR/96323/96 | DE/113/95 | CA/189/66 |
| A/Gadwall/Netherlands/1/2006***^a^*** | D10 | ST/163/04 | DE/113/95 | KR/96323/96 | DE/113/95 | KR/96323/96 | KR/96323/96 | DE/113/95 | CA/189/66 |
| A/laughing gull/Delaware/12/2006***^a^*** | D11 | WI/1/66 | WI/1/66 | WI/1/66 | DE/113/95 | WI/1/66 | WI/1/66 | WI/1/66 | WI/1/66 |
| A/shorebird/Delaware/249/2006 | D11 | WI/1/66 | WI/1/66 | WI/1/66 | DE/113/95 | WI/1/66 | WI/1/66 | WI/1/66 | WI/1/66 |
| A/sanderling/Delaware/482/2006 | D11 | WI/1/66 | WI/1/66 | WI/1/66 | DE/113/95 | WI/1/66 | WI/1/66 | WI/1/66 | WI/1/66 |
| A/Bewick swan/Netherlands/5/2007***^a^*** | D12 | ST/163/04 | DE/113/95 | KR/96323/96 | DE/113/95 | KR/96323/96 | KR/96323/96 | DE/113/95 | HK/Y439/97 |
| A/duck/Hongkong/289/78***^a^*** | E0***^b^*** | HK/289/78 | HK/289/78 | HK/289/78 | HK/289/78 | HK/289/78 | HK/289/78 | HK/289/78 | HK/289/78 |
| A/duck/Hongkong/702/1979***^a^*** | E0 | HK/289/78 | HK/289/78 | HK/289/78 | HK/289/78 | HK/289/78 | HK/289/78 | HK/289/78 | HK/289/78 |
| A/duck/Hongkong/702/1979-quail adapted | E0 | HK/289/78 | HK/289/78 | HK/289/78 | HK/289/78 | HK/289/78 | HK/289/78 | HK/289/78 | HK/289/78 |
| A/duck/Hongkong/702/1979-chicken adapted | E0 | HK/289/78 | HK/289/78 | HK/289/78 | HK/289/78 | HK/289/78 | HK/289/78 | HK/289/78 | HK/289/78 |
| A/duck/Hongkong/366/78***^a^*** | E1 | HK/289/78 | HK/289/78 | WI/1/66 | HK/289/78 | HK/289/78 | HK/289/78 | HK/289/78 | CA/189/66 |
| A/duck/Hk/784/1979***^a^*** | E2 | HK/289/78 | HK/289/78 | WI/1/66 | HK/289/78 | HK/289/78 | HK/Y439/97 | HK/289/78 | HK/289/78 |
| A/duck/Hongkong/552/79***^a^*** | E3 | HK/289/78 | HK/289/78 | ? | HK/289/78 | HK/289/78 | HK/289/78 | HK/289/78 | CA/189/66 |
| A/goose/MN/5733-1/1980***^a^*** | E4 | WI/1/66 | WI/1/66 | WI/1/66 | HK/289/78 | WI/1/66 | WI/1/66 | WI/1/66 | CA/189/66 |
| A/turkey/TX/4-1-81/1981 | E4 | WI/1/66 | WI/1/66 | WI/1/66 | HK/289/78 | WI/1/66 | WI/1/66 | WI/1/66 | CA/189/66 |
| A/mallard duck/AlB/321/1988 | E4 | WI/1/66 | WI/1/66 | WI/1/66 | HK/289/78 | WI/1/66 | WI/1/66 | WI/1/66 | CA/189/66 |
| A/turkey/TX/10-49-89/1989 | E4 | WI/1/66 | WI/1/66 | WI/1/66 | HK/289/78 | WI/1/66 | WI/1/66 | WI/1/66 | CA/189/66 |
| A/mallard/Alberta/11/1991***^a^*** | E5 | WI/1/66 | WI/1/66 | WI/1/66 | HK/289/78 | WI/1/66 | WI/1/66 | WI/1/66 | WI/1/66 |
| A/mallard/ALB/17/1991 | E5 | WI/1/66 | WI/1/66 | WI/1/66 | HK/289/78 | WI/1/66 | WI/1/66 | WI/1/66 | WI/1/66 |
| A/quail/Hongkong/AF157/92***^a^*** | F***^b^*** | HK/289/78 | HK/AF157/92 | BJ/1/94 | HK/AF157/92 | HK/AF157/92 | HK/AF157/92 | HK/AF157/92 | HK/AF157/92 |
| A/turkey/Wisconsin/1/1966***^a^*** | G0***^b^*** | WI/1/66 | WI/1/66 | WI/1/66 | WI/1/66 | WI/1/66 | WI/1/66 | WI/1/66 | WI/1/66 |
| A/turkey/WI/1966 | G0 | WI/1/66 | WI/1/66 | WI/1/66 | WI/1/66 | WI/1/66 | WI/1/66 | WI/1/66 | WI/1/66 |
| A/turkey/Wisconsin/66 | G0 | WI/1/66 | WI/1/66 | WI/1/66 | WI/1/66 | WI/1/66 | WI/1/66 | WI/1/66 | WI/1/66 |
| A/Shorebird/Delaware/9/96 | G0 | WI/1/66 | WI/1/66 | WI/1/66 | WI/1/66 | WI/1/66 | WI/1/66 | WI/1/66 | WI/1/66 |
| A/turkey/California/189/66***^a^*** | G1 | WI/1/66 | WI/1/66 | WI/1/66 | WI/1/66 | WI/1/66 | WI/1/66 | WI/1/66 | CA/189/66 |
| A/chicken/Heilongjiang/35/00***^a^*** | G2 | HK/G1/97 | HK/G1/97 | HK/G1/97 | WI/1/66 | WI/1/66 | HK/G9/97 | BJ/1/94 | BJ/1/94 |

***^a^*** H9N2 representative viruses from different genotypes, ***^b^*** various series genotypical prototype.
