# Supplementary material for: Phylogenetic Diversity and Genotypical Complexity of H9N2 Influenza A Viruses Revealed by Genomic Sequence Analysis
Source: PLoS One. 2011 Feb 28;6(2):e17212. doi: 10.1371/journal.pone.0017212 (PMC3046171; doi:10.1371/journal.pone.0017212)
Supplement: Table S2 — Accession numbers of nucleic acid sequences for the 571 H9N2 influenza A and other subtype reference viruses used in this study. (DOC) [file pone.0017212.s004.doc]

| **Virus Name** | **PB2** | **PB1** | **PA** | **HA** | **NP** | **NA** | **MP** | **NS** |
| --- | --- | --- | --- | --- | --- | --- | --- | --- |
| A/avian/Israel/584/2005(H9N2) | EF492407 | EF492378 | EF492349 | EF492228 | EF492309 | EF492280 | EF492251 | EF492337 |
| A/avian/Israel/313/2008(H9N2) | FJ464701 | FJ464684 | FJ464667 | FJ464718 | FJ464634 | FJ464617 | FJ464600 | FJ464651 |
| A/avian/Israel/314/2008(H9N2) | FJ464702 | FJ464685 | FJ464668 | FJ464719 | FJ464635 | FJ464618 | FJ464601 | FJ464652 |
| A/barnacle goose/Netherlands/1/2005(H6N2) | CY041393 | CY041392 | CY041391 | CY041386 | CY041389 | CY041388 | CY041387 | CY041390 |
| A/Bewick swan/Netherlands/1/2005(H6N2) | DQ822187 | [DQ822188](http://www.ncbi.nlm.nih.gov/entrez/viewer.fcgi?val=DQ822188) | DQ822189 | DQ822190 | DQ822191 | DQ822192 | DQ822193 | DQ822194 |
| A/Bewick swan/Netherlands/5/2007(H9N2) | CY041281 | CY041280 | CY041279 | CY041274 | CY041277 | CY041276 | CY041275 | CY041278 |
| A/bird/Guangxi/62/2005(H9N2) | EU086281 | EU086279 | EU086257 | EU086246 | EU086252 | EU086249 | EU086247 | [EU086255](http://www.ncbi.nlm.nih.gov/entrez/viewer.fcgi?val=EU086255) |
| A/bird/Guangxi/82/2005(H9N2) | EU086282 | EU086280 | EU086278 | EU086265 | EU086273 | EU086271 | EU086268 | [EU086275](http://www.ncbi.nlm.nih.gov/entrez/viewer.fcgi?val=EU086275) |
| A/bird/Guangxi/83/2005(H9N2) | EU086300 | EU086296 | EU086293 | EU086266 | EU086274 | EU086272 | EU086269 | [EU086277](http://www.ncbi.nlm.nih.gov/entrez/viewer.fcgi?val=EU086277) |
| A/bird/Guangxi/A1/2006(H9N2) | EU086301 | EU086298 | EU086295 | EU086284 | EU086290 | EU086288 | EU086286 | EU086291 |
| A/bird/Guangxi/H1/2006(H9N2) | EU086317 | EU086315 | EU086312 | EU086303 | EU086308 | EU086307 | EU086305 | EU086311 |
| A/chicken/Beijing/1/1994(H9N2) | AF156438 | AF156423 | AF156452 | AF156380 | AF156409 | AF156398 | AF156466 | AF156480 |
| A/chicken/Hong kong/739/1994(H9N2) | AF156436 | AF156422 | AF156450 | AF156379 | AF156408 | AF156397 | AF156464 | AF156478 |
| A/chicken/Korea/38349-p96323/1996(H9N2) | AF156439 | AF156425 | AF156453 | AF156384 | AF156411 | AF156400 | AF156467 | AF156481 |
| A/chicken/Shandong/6/1996(H9N2) | DQ064565 | DQ064538 | DQ064511 | DQ064376 | DQ064457 | DQ064430 | DQ064403 | [DQ064484](http://www.ncbi.nlm.nih.gov/entrez/viewer.fcgi?val=DQ064484) |
| A/chicken/Shandong/7/1996(H9N2) | DQ064566 | DQ064539 | DQ064512 | DQ064377 | DQ064458 | DQ064431 | DQ064404 | [DQ064485](http://www.ncbi.nlm.nih.gov/entrez/viewer.fcgi?val=DQ064485) |
| A/chicken/Guangdong/11/1997(H9N2) | AF508651 | AF508629 | AF508673 | AF508564 | AF508607 | AF508585 | AF508695 | AF508716 |
| A/chicken/Guangdong/5/1997(H9N2) | DQ064549 | DQ064522 | DQ064495 | DQ064360 | DQ064441 | DQ064414 | DQ064387 | DQ064468 |
| A/chicken/Guangdong/6/1997(H9N2) | DQ064551 | DQ064524 | DQ064497 | DQ064362 | DQ064443 | DQ064416 | DQ064389 | DQ064470 |
| A/chicken/Heilongjiang/10/1997(H9N2) | AF508653 | AF508631 | AF508675 | AF508566 | AF508609 | AF508587 | AF508696 | AF508718 |
| A/chicken/Hong kong/G9/1997(H9N2) | AF156430 | AF156416 | AF156444 | AF156373 | AF156402 | AF156391 | AF156458 | AF156472 |
| A/chicken/Osaka/aq48/1997(H9N2) | AB256663 | AB256664 | AB256665 | AB256666 | AB256667 | AB256668 | AB256669 | AB256670 |
| A/chicken/Shenzhen/9/1997(H9N2) | DQ064569 | DQ064516 | DQ064489 | DQ064380 | DQ064435 | DQ064408 | DQ064381 | DQ064462 |
| A/chicken/Sichuan/5/1997(H9N2) | AF508656 | AF508634 | AF508678 | AF508569 | AF508612 | AF508590 | AF508699 | AF508721 |
| A/chicken/Beijing/8/1998(H9N2) | DQ064543 | AF508627 | AF508671 | DQ064354 | DQ064461 | AF508583 | DQ064407 | DQ064488 |
| A/chicken/Germany/R45/1998(H9N2) | AF508643 | AF508621 | AF508665 | AJ781822 | AF508599 | AF508599 | AF508687 | AF508708 |
| A/chicken/Henan/5/1998(H9N2) | DQ064559 | DQ064532 | DQ064505 | DQ064370 | DQ064451 | DQ064424 | DQ064397 | [DQ064478](http://www.ncbi.nlm.nih.gov/entrez/viewer.fcgi?val=DQ064478) |
| A/chicken/Henan/nd/1998(H9N2) | DQ997447 | DQ997446 | DQ997444 | DQ997448 | DQ997442 | DQ997441 | DQ997445 | DQ997443 |
| A/chicken/Jiangsu/1/1998(H9N2) | FJ793377 | FJ793378 | FJ793379 | FJ793380 | FJ793381 | FJ793382 | FJ793383 | FJ793384 |
| A/chicken/Shandong/1/1998(H9N2) | FJ793425 | FJ793426 | FJ793427 | FJ793428 | FJ793429 | FJ793430 | FJ793431 | FJ793432 |
| A/chicken/Shanghai/F/98(H9N2) | AY253750 | AY253751 | AY253752 | AY743216 | AY253753 | AY253754 | AY253755 | AY253756 |
| A/chicken/Shijiazhuang/2/1998(H9N2) | DQ064568 | DQ064541 | DQ064514 | DQ064379 | DQ064460 | DQ064433 | DQ064406 | DQ064487 |
| A/chicken/Beijing/ne/1999(H9N2) | DQ997511 | DQ997510 | DQ997509 | DQ997505 | DQ997507 | DQ997506 | DQ997512 | DQ997508 |
| A/chicken/Gansu/2/1999(H9N2) | EF070740 | EF070739 | EF070738 | EF070733 | EF070735 | EF070734 | EF070737 | EF070736 |
| A/chicken/Guangxi/10/1999(H9N2) | DQ064552 | DQ064525 | DQ064498 | DQ064363 | DQ064444 | DQ064417 | DQ064390 | DQ064471 |
| A/chicken/Guangxi/4/1999(H9N2) | EU081871 | EU081870 | EU081869 | EU081864 | EU081867 | EU081866 | EU081865 | EU081868 |
| A/chicken/Guangxi/9/1999(H9N2) | DQ064553 | DQ064526 | DQ064499 | DQ064364 | DQ064445 | DQ064418 | DQ064391 | [DQ064472](http://www.ncbi.nlm.nih.gov/entrez/viewer.fcgi?val=DQ064472) |
| A/chicken/Hong kong/FY20/1999(H9N2) | AF222627 | AF222637 | AF222647 | AF222611 | AF222619 | AF222657 | AF222667 | AF222677 |
| A/chicken/Hong kong/KC12/1999(H9N2) | AF222628 | AF222638 | AF222648 | AF222612 | AF222620 | AF222658 | AF222668 | AF222678 |
| A/chicken/Hong kong/SF2/1999(H9N2) | AF222630 | AF222640 | AF222650 | AF186269 | AF186272 | AF222660 | AF222670 | AF222680 |
| A/chicken/Hong kong/G23/1999(H9N2) | AF156431 | AF156417 | AF156445 | AF156374 | AF156403 | AF156392 | AF156459 | AF222674 |
| A/chicken/Hongkong/NT16/1999(H9N2) | AF222624 | AF222634 | AF222644 | AF222608 | AF222616 | AF222654 | AF222664 | [AF222674](http://www.ncbi.nlm.nih.gov/entrez/viewer.fcgi?val=AF222674) |
| A/chicken/Iran/11T/1999(H9N2) | AF508645 | AF508623 | AF508667 | AF508558 | AF508601 | AF508579 | AF508689 | AF508710 |
| A/chicken/Jiangsu/1/1999(H9N2) | FJ793385 | FJ793386 | FJ793387 | AF461509 | FJ793389 | FJ793390 | FJ793391 | FJ793392 |
| A/chicken/Korea/99029/1999(H9N2) | AF508648 | AF508626 | AF508670 | AF508561 | AF508604 | AF508582 | AF508692 | AF508713 |
| A/chicken/Ningxia/4/1999(H9N2) | DQ064564 | DQ064537 | DQ064510 | DQ064375 | DQ064456 | DQ064429 | DQ064402 | DQ064483 |
| A/chicken/Ningxia/5/1999(H9N2) | AF508655 | AF508633 | AF508677 | AF508568 | AF508611 | AF508589 | AF508698 | AF508720 |
| A/chicken/Pakisatn/2/1999(H9N2) | AJ291395 | AJ291396 | AJ291397 | AJ291392 | AJ291394 | AJ291393 | AJ291398 | AJ291399 |
| A/chicken/Pakisatn/4/1999(H9N2) | AF508641 | AF508619 | AF508663 | AF508555 | AF508597 | AF508576 | AF508685 | AF508706 |
| A/chicken/Pakisatn/5/1999(H9N2) | AF508642 | AF508620 | AF508664 | AF508556 | AF508598 | AF508577 | AF508686 | AF508707 |
| A/chicken/Saudi Arabia/532/1999(H9N2) | AF508646 | AF508624 | AF508668 | AF508559 | AF508602 | AF508580 | AF508690 | AF508711 |
| A/chicken/Shandong/1/1999(H9N2) | FJ793433 | FJ793434 | FJ793435 | FJ793436 | FJ793437 | FJ793438 | FJ793439 | FJ793440 |
| A/chicken/Shanghai/2/1999(H9N2) | EU753271 | EU753272 | EU753273 | EU753274 | EU753275 | EU753276 | EU753277 | EU753278 |
| A/chicken/Shijiazhuang/2/1999(H9N2) | AF508658 | AF508636 | AF508680 | AF508571 | AF508614 | AF508592 | AF508701 | AF508723 |
| A/chicken/china/Guangxi1/2000(H9N2) | DQ485205 | DQ485206 | DQ485207 | DQ485208 | DQ485209 | DQ485210 | DQ485211 | DQ485212 |
| A/chicken/china/Guangxi14/2000(H9N2) | DQ485213 | DQ485214 | DQ485215 | DQ485216 | DQ485217 | DQ485218 | DQ485219 | DQ485220 |
| A/chicken/china/Guangxi17/2000(H9N2) | DQ485221 | DQ485222 | DQ485223 | DQ485224 | DQ485225 | DQ485226 | DQ485227 | DQ485228 |
| A/chicken/Fujian/25/2000(H9N2) | DQ064544 | DQ064517 | DQ064490 | DQ064355 | DQ064436 | DQ064409 | DQ064382 | DQ064463 |
| A/chicken/Guangdong/10/2000(H9N2) | DQ064545 | DQ064518 | DQ064491 | DQ064356 | DQ064437 | DQ064410 | DQ064383 | DQ064464 |
| A/chicken/Guangdong/4/2000(H9N2) | DQ064548 | DQ064520 | DQ064493 | DQ064358 | DQ064439 | DQ064412 | DQ064385 | DQ064466 |
| A/chicken/Guangxi/6/2000(H9N2) | EU086244 | EU086243 | EU086242 | EU086237 | EU086240 | EU086239 | EU086238 | EU086241 |
| A/chicken/Hebei/31/2000(H9N2) | DQ064554 | DQ064527 | DQ064500 | DQ064365 | DQ064446 | DQ064419 | DQ064392 | DQ064473 |
| A/chicken/Heilongjiang/35/2000(H9N2) | DQ064555 | DQ064528 | DQ064501 | DQ064366 | DQ064447 | DQ064420 | DQ064393 | DQ064474 |
| A/chicken/Henan/26/2000(H9N2) | DQ064557 | DQ064530 | DQ064503 | DQ064368 | DQ064449 | DQ064422 | DQ064395 | DQ064476 |
| A/chicken/Henan/62/2000(H9N2) | AF508654 | AF508632 | AF508676 | AF508567 | AF508610 | AF508588 | AF508697 | AF508719 |
| A/chicken/Israel/90658/2000(H9N2) | EF492428 | EF492399 | EF492370 | EF492221 | EF492330 | EF492301 | EF492272 | DQ683047 |
| A/chicken/Jiangsu/1/2000(H9N2) | DQ064561 | DQ064534 | DQ064507 | DQ064372 | DQ064453 | DQ064426 | DQ064399 | DQ064480 |
| A/chicken/Shandong/1/2000(H9N2) | FJ793401 | FJ793402 | FJ793403 | FJ793404 | FJ793405 | FJ793406 | FJ793407 | FJ793408 |
| A/chicken/Shanghai/3/2000(H9N2) | EU753279 | EU753280 | EU753281 | AF461523 | EU753283 | EU753284 | EU753285 | EU753286 |
| A/chicken/Shantou/1322/2000(H9N2) | CY024589 | CY024590 | CY024591 | CY024592 | CY024593 | CY024594 | CY024595 | CY024596 |
| A/chicken/Shantou/1690/2000(H9N2) | CY024605 | CY024606 | CY024607 | CY024608 | CY024609 | CY024610 | CY024611 | [CY024612](http://www.ncbi.nlm.nih.gov/entrez/viewer.fcgi?val=CY024612) |
| A/chicken/Shantou/2098/2000(H9N2) | CY024621 | CY024622 | CY024623 | CY024624 | CY024625 | CY024626 | CY024627 | CY024628 |
| A/chicken/Shantou/212/2000(H9N2) | CY024573 | CY024574 | CY024575 | CY024576 | CY024577 | CY024578 | CY024579 | CY024580 |
| A/chicken/Shantou/859/2000(H9N2) | CY024581 | CY024582 | CY024583 | CY024584 | CY024585 | CY024586 | CY024587 | CY024588 |
| A/chicken/Shantou/94/2000(H9N2) | CY024565 | CY024566 | CY024567 | CY024568 | CY024569 | CY024570 | CY024571 | CY024572 |
| A/chicken/Dubai/338/2001(H9N2) | EF063555 | EF063534 | EF063548 | EF063513 | EF063527 | EF063520 | EF063506 | EF063541 |
| A/chicken/Dubai/339/2001(H9N2) | EF063556 | EF063535 | EF063549 | EF063514 | EF063528 | EF063521 | EF063507 | EF063542 |
| A/chicken/Guangdong/47/2001(H9N2) | DQ064547 | DQ064521 | DQ064494 | DQ064359 | DQ064440 | DQ064413 | DQ064386 | DQ064467 |
| A/chicken/Guangdong/56/2001(H9N2) | DQ064550 | DQ064523 | DQ064496 | DQ064361 | DQ064442 | DQ064415 | DQ064388 | DQ064469 |
| A/chicken/Hebei/B1/2001(H9N2) | EU914195 | EU939158 | EU532063 | EU573938 | EU532037 | EU346934 | EU532029 | EU532047 |
| A/chicken/Helongjiang/48/01(H9N2) | DQ064556 | DQ064529 | DQ064502 | DQ064367 | DQ064448 | DQ064421 | DQ064394 | DQ064475 |
| A/chicken/Henan/ni/2001(H9N2) | DQ997476 | DQ997475 | DQ997480 | DQ997474 | DQ997478 | DQ997477 | DQ997473 | DQ997479 |
| A/Chicken/Hong Kong/715.5/01 (H5N1) | AF509152 | [AF509049](http://www.ncbi.nlm.nih.gov/entrez/viewer.fcgi?val=AF509049) | [AF509075](http://www.ncbi.nlm.nih.gov/entrez/viewer.fcgi?val=AF509075) | AF509025 | AF509126 | AF509100 | AF509049 | AF509075 |
| A/Chicken/Hong Kong/YU562/01 (H5N1) | AY221592 | [AF509170](http://www.ncbi.nlm.nih.gov/entrez/viewer.fcgi?val=AF509170) | AF509196 | AF509017 | AF509118 | AF509093 | AF509041 | AF509067 |
| A/chicken/Israel/786/2001(H9N2) | EF492410 | EF492381 | EF492352 | EF492231 | EF492312 | EF492283 | [EF492269](http://www.ncbi.nlm.nih.gov/entrez/viewer.fcgi?val=EF492269) | DQ683032 |
| A/chicken/Jiangsu/2/2001(H9N2) | FJ793393 | FJ793394 | FJ793395 | FJ793396 | FJ793397 | FJ793398 | FJ793399 | FJ793400 |
| A/chicken/Jiangsu/ng/2001(H9N2) | DQ997459 | DQ997458 | DQ997464 | DQ997460 | DQ997462 | DQ997461 | DQ997457 | DQ997463 |
| A/chicken/Jilin/53/2001(H9N2) | DQ064560 | DQ064533 | DQ064506 | DQ064371 | DQ064452 | DQ064425 | DQ064398 | DQ064479 |
| A/chicken/Kobe/aq26/2001(H9N2) | AB256679 | AB256680 | AB256681 | AB256682 | AB256683 | AB256684 | AB256685 | AB256686 |
| A/Chicken/Nanchang/3-120/2001 (H3N2) | AY180761 | AY180871 | AY180677 | AY180412 | AY180555 | AY180814 | CY005445 | AY180611 |
| A/chicken/Nanchang/4-301/2001(H9N2) | CY005524 | CY005523 | [CY005522](http://www.ncbi.nlm.nih.gov/entrez/viewer.fcgi?val=CY005522) | CY006023 | CY005521 | CY005520 | CY005519 | CY006024 |
| A/chicken/Osaka/aq19/2001(H9N2) | AB256735 | AB256736 | [AB256737](http://www.ncbi.nlm.nih.gov/entrez/viewer.fcgi?val=AB256737) | AB256738 | AB256739 | AB256740 | AB256741 | AB256742 |
| A/chicken/Osaka/aq58/2001(H9N2) | AB256687 | AB256688 | AB256689 | AB256690 | AB256691 | AB256692 | AB256693 | AB256694 |
| A/chicken/Osaka/aq69/2001(H9N2) | AB256695 | AB256696 | AB256697 | AB256698 | AB256699 | AB256700 | AB256701 | AB256702 |
| A/chicken/Shanghai/1/2001(H9N2) | EU753287 | EU753288 | [EU753289](http://www.ncbi.nlm.nih.gov/entrez/viewer.fcgi?val=EU753289) | EU753290 | EU753291 | EU753292 | EU753293 | EU753294 |
| A/chicken/Shanghai/10/01(H9N2) | DQ064567 | DQ064540 | DQ064513 | DQ064378 | DQ064459 | DQ064432 | DQ064405 | DQ064486 |
| A/chicken/Shanghai/14/2001(H9N2) | EU753319 | EU753320 | EU753321 | EU753322 | EU753323 | EU753324 | EU753325 | EU753326 |
| A/chicken/Shanghai/16/2001(H9N2) | EU753327 | EU753328 | EU753329 | EU753330 | EU753331 | EU753332 | EU753333 | EU753334 |
| A/chicken/Shanghai/2/2001(H9N2) | EU753295 | EU753296 | EU753297 | EU753298 | EU753299 | EU753300 | EU753301 | EU753302 |
| A/chicken/Shanghai/7/2001(H9N2) | EU753303 | EU753304 | EU753305 | EU753306 | EU753307 | EU753308 | EU753309 | EU753310 |
| A/chicken/Shantou/1126/2001(H9N2) | CY024629 | CY024630 | CY024631 | CY024632 | CY024633 | CY024634 | CY024635 | CY024636 |
| A/chicken/Shantou/1205/2001(H9N2) | CY024637 | CY024638 | CY024639 | CY024640 | CY024641 | CY024642 | CY024643 | CY024644 |
| A/chicken/Shantou/1608/2001(H9N2) | CY024653 | CY024654 | CY024655 | CY024656 | CY024657 | CY024658 | CY024659 | CY024660 |
| A/chicken/Shantou/1610/2001(H9N2) | CY024661 | CY024662 | [CY024663](http://www.ncbi.nlm.nih.gov/entrez/viewer.fcgi?val=CY024663) | CY024664 | CY024665 | CY024666 | CY024667 | CY024668 |
| A/chicken/Shantou/1890/2001(H9N2) | CY024669 | CY024670 | CY024671 | CY024672 | CY024673 | CY024674 | CY024675 | CY024676 |
| A/chicken/Shantou/2712/2001(H9N2) | CY024685 | CY024686 | CY024687 | CY024688 | [CY024689](http://www.ncbi.nlm.nih.gov/entrez/viewer.fcgi?val=CY024689) | CY024690 | CY024691 | CY024692 |
| A/chicken/Shantou/3173/2001(H9N2) | CY024693 | CY024694 | CY024695 | CY024696 | CY024697 | CY024698 | CY024699 | CY024700 |
| A/chicken/Shantou/3778/2001(H9N2) | CY024701 | CY024702 | CY024703 | CY024704 | CY024705 | CY024706 | [CY024707](http://www.ncbi.nlm.nih.gov/entrez/viewer.fcgi?val=CY024707) | CY024708 |
| A/chicken/Shantou/4208/2001(H9N2) | CY024709 | CY024710 | CY024711 | CY024712 | [CY024713](http://www.ncbi.nlm.nih.gov/entrez/viewer.fcgi?val=CY024713) | CY024714 | CY024715 | [CY024716](http://www.ncbi.nlm.nih.gov/entrez/viewer.fcgi?val=CY024716) |
| A/chicken/Shantou/5028/2001(H9N2) | CY024717 | CY024718 | CY024719 | CY024720 | CY024721 | CY024722 | CY024723 | CY024724 |
| A/chicken/Shantou/5319/2001(H9N2) | CY024725 | CY024726 | [CY024727](http://www.ncbi.nlm.nih.gov/entrez/viewer.fcgi?val=CY024727) | CY024728 | CY024729 | CY024730 | CY024731 | CY024732 |
| A/chicken/Shantou/5714/2001(H9N2) | CY023101 | CY023102 | CY023103 | CY023104 | CY023105 | CY023106 | CY023107 | CY023108 |
| A/chicken/YoKohama/aq120/2001(H9N2) | AB256727 | AB256728 | AB256729 | AB256730 | AB256731 | AB256732 | AB256733 | AB256734 |
| A/chicken/YoKohama/aq135/2001(H9N2) | AB256719 | AB256720 | AB256721 | AB256722 | AB256723 | AB256724 | AB256725 | AB256726 |
| A/chicken/YoKohama/aq144/2001(H9N2) | AB256743 | AB256744 | AB256745 | AB256746 | AB256747 | AB256748 | AB256749 | AB256750 |
| A/chicken/YoKohama/aq55/2001(H9N2) | AB256671 | AB256672 | AB256673 | AB256674 | AB256675 | AB256676 | AB256677 | AB256678 |
| A/chicken/Yunnan/nh/2001(H9N2) | DQ997467 | DQ997466 | DQ997471 | DQ997465 | DQ997469 | DQ997468 | DQ997472 | DQ997470 |
| A/chicken/Anhui/2/2002(H9N2) | FJ793313 | FJ793314 | FJ793315 | FJ793316 | FJ793317 | FJ793318 | FJ793319 | FJ793320 |
| A/chicken/Beijing/nl/2002(H9N2) | DQ997504 | DQ997503 | DQ997502 | DQ997497 | DQ997500 | DQ997499 | DQ997498 | DQ997501 |
| A/chicken/Dubai/383/2002(H9N2) | EF063557 | EF063536 | EF063550 | EF063515 | EF063529 | EF063522 | EF063508 | EF063543 |
| A/chicken/Guangdong/21/2002(H9N2) | DQ064546 | DQ064519 | DQ064492 | DQ064357 | DQ064438 | DQ064411 | DQ064384 | DQ064465 |
| A/chicken/Hebei/nj/2002(H9N2) | DQ997486 | DQ997485 | DQ997487 | DQ997481 | DQ997483 | DQ997482 | DQ997488 | DQ997484 |
| A/chicken/Henan/1/2002(H9N2) | FJ793321 | FJ793322 | FJ793323 | FJ793324 | FJ793325 | FJ793326 | FJ793327 | FJ793328 |
| A/chicken/Henan/43/2002(H9N2) | DQ064558 | DQ064531 | DQ064504 | DQ064369 | DQ064450 | DQ064423 | DQ064396 | DQ064477 |
| A/chicken/Hunan/774/2002(H9N2) | CY023765 | CY023766 | CY023767 | CY023768 | CY023769 | CY023770 | CY023771 | CY023772 |
| A/chicken/Jiangsu/1/2002(H9N2) | FJ793361 | FJ793362 | FJ793363 | FJ793364 | FJ793365 | FJ793366 | FJ793367 | FJ793368 |
| A/chicken/Jiangsu/7/2002(H9N2) | FJ384748 | FJ384749 | FJ384750 | FJ384751 | FJ384744 | FJ384753 | FJ384754 | FJ384755 |
| A/chicken/Jiangsu/cz1/2002(H5N1) | DQ997181 | DQ997180 | [DQ997186](http://www.ncbi.nlm.nih.gov/entrez/viewer.fcgi?val=DQ997186) | [DQ997182](http://www.ncbi.nlm.nih.gov/entrez/viewer.fcgi?val=DQ997182) | DQ997184 | DQ997183 | DQ997179 | DQ997185 |
| A/chicken/Jiangsu/wa/2002(H9N2) | DQ997193 | DQ997192 | DQ997191 | DQ997187 | DQ997189 | DQ997188 | DQ997194 | DQ997190 |
| A/chicken/Neimenggu/nK/2002(H9N2) | DQ997496 | DQ997495 | DQ997494 | DQ997490 | DQ997492 | DQ997491 | DQ997489 | DQ997493 |
| A/chicken/Shanghai/1/2002(H9N2) | EU753335 | [EU753336](http://www.ncbi.nlm.nih.gov/entrez/viewer.fcgi?val=EU753336) | [EU753337](http://www.ncbi.nlm.nih.gov/entrez/viewer.fcgi?val=EU753337) | AY281745 | EU753339 | EU753340 | EU753341 | EU753342 |
| A/chicken/Shanghai/2/2002(H9N2) | EU753343 | EU753344 | EU753345 | EU753346 | EU753347 | EU753348 | EU753349 | EU753350 |
| A/chicken/Shantou/2204/2002(H9N2) | CY023133 | CY023134 | CY023135 | CY023136 | CY023137 | CY023138 | CY023139 | CY023140 |
| A/chicken/Shantou/4/2002(H9N2) | CY023109 | CY023110 | CY023111 | CY023112 | CY023113 | CY023114 | CY023115 | CY023116 |
| A/chicken/Shantou/4144/2002(H9N2) | CY023141 | CY023142 | CY023143 | CY023144 | CY023145 | CY023146 | CY023147 | CY023148 |
| A/chicken/Shantou/4342/2002(H9N2) | CY023149 | CY023150 | CY023151 | CY023152 | [CY023121](http://www.ncbi.nlm.nih.gov/entrez/viewer.fcgi?val=CY023121) | CY023154 | CY023155 | CY023156 |
| A/chicken/Shantou/439/2002(H9N2) | CY023117 | CY023118 | CY023119 | CY023120 | CY023121 | CY023122 | CY023123 | CY023124 |
| A/chicken/Shantou/4608/2002(H9N2) | CY023157 | CY023158 | CY023159 | CY023160 | CY023161 | CY023162 | CY023163 | CY023164 |
| A/chicken/YoKohama/aq134/2002(H9N2) | AB256711 | AB256712 | AB256713 | AB256714 | AB256715 | AB256716 | AB256717 | AB256718 |
| A/chicken/YoKohama/aq45/2002(H9N2) | AB256703 | AB256704 | AB256705 | AB256706 | AB256707 | AB256708 | AB256709 | AB256710 |
| A/chicken/Yunnan/401/2002(H9N2) | CY023845 | CY023846 | CY023847 | CY023848 | CY023849 | CY023850 | CY023851 | CY023852 |
| A/chicken/Yunnan/522/2002(H9N2) | CY023853 | CY023854 | CY023855 | CY023856 | CY023857 | CY023858 | CY023859 | CY023860 |
| A/chicken/Yunnan/955/2002(H9N2) | CY023861 | CY023862 | [CY023863](http://www.ncbi.nlm.nih.gov/entrez/viewer.fcgi?val=CY023863) | CY023864 | CY023865 | CY023866 | CY023867 | [CY023868](http://www.ncbi.nlm.nih.gov/entrez/viewer.fcgi?val=CY023868) |
| A/chicken/Dubai/463/2003(H9N2) | EF063558 | EF063537 | EF063551 | EF063516 | EF063530 | EF063523 | EF063509 | EF063544 |
| A/chicken/Henan/1/2003(H9N2) | FJ793329 | FJ793330 | FJ793331 | FJ793332 | FJ793333 | FJ793334 | FJ793335 | FJ793336 |
| A/chicken/HK/TP38/2003(H9N2) | AY664802 | AY664784 | AY664765 | AY664670 | AY664727 | AY664708 | AY664689 | AY664746 |
| A/chicken/HongKong/AP45/2003(H9N2) | AY664793 | AY664775 | AY664756 | AY664661 | AY664718 | AY664699 | AY664680 | AY664737 |
| A/chicken/HongKong/BD90/2003(H9N2) | AY664794 | AY664776 | AY664757 | AY664662 | AY664719 | AY664700 | AY664681 | AY664738 |
| A/chicken/HongKong/CSW153/2003(H9N2) | AY664792 | AY664774 | AY664755 | AY664660 | AY664717 | AY664698 | AY664679 | AY664736 |
| A/chicken/Hong Kong/FY23/2003(H9N2) | AY664797 | AY664779 | AY664760 | AY664665 | AY664722 | AY664703 | AY664684 | AY664741 |
| A/chicken/HongKong/SF1/2003(H9N2) | AY664800 | AY664782 | AY664763 | AY664668 | AY664725 | AY664706 | AY664687 | AY664744 |
| A/chicken/HongKong/WF120/2003(H9N2) | AY664807 | AY664789 | AY664770 | AY664675 | AY664732 | AY664713 | AY664694 | AY664751 |
| A/chicken/Israel/1304/2003(H9N2) | EF492419 | EF492390 | EF492361 | / | EF492321 | EF492292 | EF492259 | DQ683038 |
| A/chicken/Israel/1376/2003(H9N2) | EF492420 | EF492391 | EF492362 | EF492239 | EF492322 | EF492293 | EF492260 | DQ683039 |
| A/chicken/Israel/1475/2003(H9N2) | EF492422 | EF492393 | EF492364 | EF492240 | EF492324 | EF492295 | EF492262 | DQ683041 |
| A/chicken/Korea/S1/2003(H9N2) | AY800240 | AY800239 | AY800238 | AY790313 | AY800236 | AY800235 | AY800234 | AY800237 |
| A/chicken/Korea/S16/2003(H9N2) | AY862717 | AY862701 | AY862685 | AY862605 | AY862653 | AY862637 | AY862621 | AY862669 |
| A/chicken/Korea/S5/2003(H9N2) | AY862712 | AY862696 | AY862680 | AY862600 | AY862648 | AY862632 | AY862616 | AY862664 |
| A/chicken/Shantou/1404/2003(H9N2) | CY023189 | CY023190 | CY023191 | CY023192 | CY023193 | CY023194 | CY023195 | CY023196 |
| A/chicken/Shantou/1689/2003(H9N2) | CY023197 | CY023198 | CY023199 | CY023200 | CY023201 | CY023202 | CY023203 | CY023204 |
| A/chicken/Shantou/2686/2003(H9N2) | CY023205 | CY023206 | CY023207 | CY023208 | CY023209 | CY023210 | CY023211 | CY023212 |
| A/chicken/Shantou/3040/2003(H9N2) | CY023213 | CY023214 | [CY023215](http://www.ncbi.nlm.nih.gov/entrez/viewer.fcgi?val=CY023215) | CY023216 | CY023217 | CY023218 | CY023219 | CY023220 |
| A/chicken/Shantou/3341/2003(H9N2) | CY023221 | CY023222 | CY023223 | CY023224 | CY023225 | CY023226 | CY023227 | CY023228 |
| A/chicken/Shantou/4101/2003(H9N2) | CY023229 | CY023230 | CY023231 | CY023232 | CY023233 | CY023234 | CY023235 | CY023236 |
| A/chicken/Shantou/4435/2003(H9N2) | CY023237 | CY023238 | CY023239 | CY023240 | CY023241 | CY023242 | [CY023243](http://www.ncbi.nlm.nih.gov/entrez/viewer.fcgi?val=CY023243) | CY023244 |
| A/chicken/Shantou/4617/2003(H9N2) | CY023253 | CY023254 | CY023255 | CY023256 | [CY023257](http://www.ncbi.nlm.nih.gov/entrez/viewer.fcgi?val=CY023257) | CY023258 | CY023259 | CY023260 |
| A/chicken/Shantou/69/2003(H9N2) | CY023165 | CY023166 | CY023167 | CY023168 | CY023169 | CY023170 | CY023171 | CY023172 |
| A/chicken/Shantou/944/2003(H9N2) | CY023173 | CY023174 | CY023175 | CY023176 | CY023177 | CY023178 | CY023179 | CY023180 |
| A/chicken/Yunnan/1147/2003(H9N2) | CY023869 | CY023870 | [CY023871](http://www.ncbi.nlm.nih.gov/entrez/viewer.fcgi?val=CY023871) | CY023872 | CY023873 | CY023874 | CY023875 | CY023876 |
| A/chicken/Yunnan/1252/2003(H5N1) | CY029389 | CY029390 | CY029391 | CY028980 | CY029392 | CY029393 | CY029394 | CY029395 |
| A/chicken/Yunnan/5653/2003(H9N2) | CY023877 | CY023878 | CY023879 | CY023880 | CY023881 | CY023882 | CY023883 | CY023884 |
| A/chicken/Guangxi/1857/2004(H9N2) | CY023693 | CY023694 | CY023695 | CY023696 | CY023697 | CY023698 | CY023699 | CY023700 |
| A/chicken/Guangxi/2441/2004(H9N2) | CY023701 | CY023702 | CY023703 | CY023704 | CY023705 | CY023706 | CY023707 | CY023708 |
| A/chicken/Henan/01/2004(H5N1) | AY950279 | [AY950272](http://www.ncbi.nlm.nih.gov/entrez/viewer.fcgi?val=AY950272) | [AY950265](http://www.ncbi.nlm.nih.gov/entrez/viewer.fcgi?val=AY950265) | AY950230 | AY950251 | AY950244 | AY950237 | AY950258 |
| A/chicken/Henan/1/2004(H9N2) | FJ793337 | [FJ793338](http://www.ncbi.nlm.nih.gov/entrez/viewer.fcgi?val=FJ793338) | [FJ793339](http://www.ncbi.nlm.nih.gov/entrez/viewer.fcgi?val=FJ793339) | FJ793340 | FJ793341 | FJ793342 | FJ793343 | FJ793344 |
| A/chicken/Henan/2/2004(H9N2) | FJ793353 | FJ793354 | FJ793355 | FJ793356 | FJ793357 | FJ793358 | FJ793359 | FJ793360 |
| A/chicken/Israel/1808/2004(H9N2) | EF492425 | EF492396 | EF492367 | EF492242 | EF492327 | EF492298 | EF492265 | DQ683044 |
| A/chicken/Israel/1953/2004(H9N2) | EF492426 | EF492397 | EF492368 | EF501983 | EF492328 | EF492299 | EF492266 | DQ683045 |
| A/chicken/Israel/1966/2004(H9N2) | EF492427 | EF492398 | EF492369 | EF492243 | EF492329 | EF492300 | EF492267 | DQ683046 |
| A/chicken/Jiangsu/1/2004(H9N2) | FJ793369 | FJ793370 | FJ793371 | FJ793372 | FJ793373 | FJ793374 | FJ793375 | FJ793376 |
| A/chicken/Jiangsu/L1/2004(H9N2) | EU914199 | EU939156 | EU532064 | EU939150 | EU532041 | EU346937 | EU532032 | EU532051 |
| A/chicken/Korea/S21/2004(H9N2) | EU662960 | [EU662958](http://www.ncbi.nlm.nih.gov/entrez/viewer.fcgi?val=EU662958) | EU662956 | EU662946 | EU662952 | EU662950 | EU662948 | DQ299833 |
| A/chicken/Shandong/1/2004(H9N2) | FJ793417 | FJ793418 | FJ793419 | FJ793420 | FJ793421 | FJ793422 | FJ793423 | FJ793424 |
| A/chicken/Shantou/1138/2004(H9N2) | CY023293 | CY023294 | CY023295 | CY023296 | CY023297 | CY023298 | CY023299 | CY023300 |
| A/chicken/Shantou/1926/2004(H9N2) | CY023309 | CY023310 | [CY023311](http://www.ncbi.nlm.nih.gov/entrez/viewer.fcgi?val=CY023311) | CY023312 | [CY023313](http://www.ncbi.nlm.nih.gov/entrez/viewer.fcgi?val=CY023313) | CY023314 | CY023315 | CY023316 |
| A/chicken/Shantou/2402/2004(H9N2) | CY023317 | CY023318 | CY023319 | CY023320 | CY023321 | CY023322 | CY023323 | CY023324 |
| A/chicken/Shantou/2692/2004(H9N2) | CY023325 | [CY023326](http://www.ncbi.nlm.nih.gov/entrez/viewer.fcgi?val=CY023326) | CY023327 | CY023328 | CY023329 | CY023330 | CY023331 | CY023332 |
| A/chicken/Shantou/2994/2004(H9N2) | CY023333 | CY023334 | CY023335 | CY023336 | CY023337 | CY023338 | CY023339 | CY023340 |
| A/chicken/Shantou/4726/2004(H9N2) | CY023341 | CY023342 | CY023343 | CY023344 | CY023345 | CY023346 | CY023347 | CY023348 |
| A/chicken/Shantou/5630/2004(H9N2) | CY023349 | CY023350 | CY023351 | CY023352 | CY023353 | CY023354 | CY023355 | CY023356 |
| A/chicken/Shantou/6319/2004(H9N2) | CY023373 | CY023374 | CY023375 | CY023376 | [CY023377](http://www.ncbi.nlm.nih.gov/entrez/viewer.fcgi?val=CY023377) | CY023378 | [CY023379](http://www.ncbi.nlm.nih.gov/entrez/viewer.fcgi?val=CY023379) | CY023380 |
| A/chicken/Shantou/6786/2004(H9N2) | CY023381 | CY023382 | CY023383 | CY023384 | CY023385 | CY023386 | CY023387 | CY023388 |
| A/chicken/Shantou/6911/2004(H9N2) | CY023389 | CY023390 | CY023391 | CY023392 | CY023393 | CY023394 | [CY023395](http://www.ncbi.nlm.nih.gov/entrez/viewer.fcgi?val=CY023395) | CY023396 |
| A/chicken/Shantou/7920/2004(H9N2) | CY023405 | CY023406 | CY023407 | CY023408 | CY023409 | CY023410 | CY023411 | CY023412 |
| A/chicken/Shantou/99/2004(H9N2) | CY023261 | CY023262 | CY023263 | CY023264 | CY023265 | CY023266 | CY023267 | CY023268 |
| A/chicken/Tianjin/B1/2004(H9N2) | EU914200 | EU882862 | EU532060 | EU939151 | EU532043 | EU346939 | EU532034 | EU532053 |
| A/chicken/Yunnan/3511/2004(H9N2) | CY023893 | CY023894 | CY023895 | CY023896 | CY023897 | CY023898 | CY023899 | CY023900 |
| A/chicken/Yunnan/3727/2004(H9N2) | CY023901 | CY023902 | CY023903 | CY023904 | CY023905 | CY023906 | CY023907 | [CY023908](http://www.ncbi.nlm.nih.gov/entrez/viewer.fcgi?val=CY023908) |
| A/chicken/Beijing/L1/2005(H9N2) | EU914197 | EU939160 | EU532058 | EU573940 | EU532039 | EU346935 | EU532030 | EU532049 |
| A/chicken/Fujian/10308/2005(H9N2) | CY023653 | CY023654 | [CY023655](http://www.ncbi.nlm.nih.gov/entrez/viewer.fcgi?val=CY023655) | CY023656 | [CY023657](http://www.ncbi.nlm.nih.gov/entrez/viewer.fcgi?val=CY023657) | CY023658 | CY023659 | [CY023660](http://www.ncbi.nlm.nih.gov/entrez/viewer.fcgi?val=CY023660) |
| A/chicken/Fujian/10954/2005(H9N2) | CY023661 | CY023662 | [CY023663](http://www.ncbi.nlm.nih.gov/entrez/viewer.fcgi?val=CY023663) | CY023664 | CY023665 | CY023666 | CY023667 | CY023668 |
| A/chicken/Fujian/11302/2005(H9N2) | CY023669 | CY023670 | [CY023671](http://www.ncbi.nlm.nih.gov/entrez/viewer.fcgi?val=CY023671) | CY023672 | [CY023673](http://www.ncbi.nlm.nih.gov/entrez/viewer.fcgi?val=CY023673) | CY023674 | CY023675 | CY023676 |
| A/chicken/Fujian/11488/2005(H9N2) | CY023677 | CY023678 | CY023679 | CY023680 | [CY023681](http://www.ncbi.nlm.nih.gov/entrez/viewer.fcgi?val=CY023681) | CY023682 | CY023683 | CY023684 |
| A/chicken/Fujian/12252/2005(H9N2) | CY023685 | CY023686 | CY023687 | CY023688 | [CY023689](http://www.ncbi.nlm.nih.gov/entrez/viewer.fcgi?val=CY023689) | CY023690 | CY023691 | CY023692 |
| A/chicken/Fujian/3080/2005(H9N2) | CY023541 | CY023542 | CY023543 | CY023544 | [CY023545](http://www.ncbi.nlm.nih.gov/entrez/viewer.fcgi?val=CY023545) | CY023546 | CY023547 | CY023548 |
| A/chicken/Fujian/4332/2005(H9N2) | CY023549 | CY023550 | CY023551 | CY023552 | CY023553 | CY023554 | CY023555 | [CY023556](http://www.ncbi.nlm.nih.gov/entrez/viewer.fcgi?val=CY023556) |
| A/chicken/Fujian/4820/2005(H9N2) | CY023557 | CY023558 | CY023559 | CY023560 | CY023561 | CY023562 | CY023563 | [CY023564](http://www.ncbi.nlm.nih.gov/entrez/viewer.fcgi?val=CY023564) |
| A/chicken/Fujian/5214/2005(H9N2) | CY023565 | CY023566 | CY023567 | CY023568 | [CY023569](http://www.ncbi.nlm.nih.gov/entrez/viewer.fcgi?val=CY023569) | CY023570 | CY023571 | CY023572 |
| A/chicken/Fujian/5683/2005(H9N2) | CY023573 | CY023574 | CY023575 | CY023576 | CY023577 | CY023578 | CY023579 | [CY023580](http://www.ncbi.nlm.nih.gov/entrez/viewer.fcgi?val=CY023580) |
| A/chicken/Fujian/6188/2005(H9N2) | CY023581 | CY023582 | CY023583 | CY023584 | [CY023585](http://www.ncbi.nlm.nih.gov/entrez/viewer.fcgi?val=CY023585) | CY023586 | CY023587 | CY023588 |
| A/chicken/Fujian/6960/2005(H9N2) | CY023589 | CY023590 | CY023591 | CY023592 | [CY023593](http://www.ncbi.nlm.nih.gov/entrez/viewer.fcgi?val=CY023593) | CY023594 | CY023595 | [CY023596](http://www.ncbi.nlm.nih.gov/entrez/viewer.fcgi?val=CY023596) |
| A/chicken/Fujian/7386/2005(H9N2) | CY023597 | CY023598 | CY023599 | CY023600 | [CY023601](http://www.ncbi.nlm.nih.gov/entrez/viewer.fcgi?val=CY023601) | CY023602 | CY023603 | CY023604 |
| A/chicken/Fujian/7884/2005(H9N2) | CY023605 | CY023606 | [CY023607](http://www.ncbi.nlm.nih.gov/entrez/viewer.fcgi?val=CY023607) | CY023608 | [CY023609](http://www.ncbi.nlm.nih.gov/entrez/viewer.fcgi?val=CY023609) | CY023610 | CY023611 | [CY023612](http://www.ncbi.nlm.nih.gov/entrez/viewer.fcgi?val=CY023612) |
| A/chicken/Fujian/8341/2005(H9N2) | CY023613 | CY023614 | CY023615 | CY023616 | CY023617 | CY023618 | CY023619 | CY023620 |
| A/chicken/Fujian/9104/2005(H9N2) | CY023629 | CY023630 | [CY023631](http://www.ncbi.nlm.nih.gov/entrez/viewer.fcgi?val=CY023631) | CY023632 | CY023633 | CY023634 | CY023635 | [CY023636](http://www.ncbi.nlm.nih.gov/entrez/viewer.fcgi?val=CY023636) |
| A/chicken/Fujian/9290/2005(H9N2) | CY023637 | CY023638 | CY023639 | CY023640 | [CY023641](http://www.ncbi.nlm.nih.gov/entrez/viewer.fcgi?val=CY023641) | CY023642 | CY023643 | CY023644 |
| A/chicken/Fujian/9752/2005(H9N2) | CY023645 | CY023646 | CY023647 | CY023648 | [CY023649](http://www.ncbi.nlm.nih.gov/entrez/viewer.fcgi?val=CY023649) | CY023650 | CY023651 | [CY023652](http://www.ncbi.nlm.nih.gov/entrez/viewer.fcgi?val=CY023652) |
| A/chicken/Guangxi/1032/2005(H9N2) | CY023733 | CY023734 | CY023735 | CY023736 | CY023737 | CY023738 | CY023739 | CY023740 |
| A/chicken/Guangxi/1428/2005(H9N2) | CY023741 | CY023742 | CY023743 | CY023744 | CY023745 | CY023746 | CY023747 | CY023748 |
| A/chicken/Guangxi/187/2005(H9N2) | CY023717 | CY023718 | CY023719 | CY023720 | CY023721 | CY023722 | CY023723 | [CY023724](http://www.ncbi.nlm.nih.gov/entrez/viewer.fcgi?val=CY023724) |
| A/chicken/Guangxi/2389/2005(H9N2) | CY023749 | CY023750 | CY023751 | CY023752 | CY023753 | CY023754 | CY023755 | CY023756 |
| A/chicken/Guangxi/37/2005(H9N2) | EU086233 | EU086232 | EU086231 | EU086226 | EU086229 | EU086228 | EU086227 | EU086230 |
| A/chicken/Guangxi/4745/2005(H9N2) | CY023757 | CY023758 | CY023759 | CY023760 | CY023761 | CY023762 | CY023763 | CY023764 |
| A/chicken/Guangxi/521/2005(H9N2) | CY023725 | CY023726 | CY023727 | CY023728 | CY023729 | CY023730 | CY023731 | CY023732 |
| A/chicken/Guangxi/55/2005(H9N2) | EU086263 | EU086261 | EU086258 | EU086245 | EU086253 | EU086250 | EU086248 | EU086256 |
| A/chicken/Henan/1/2005(H9N2) | FJ793345 | FJ793346 | FJ793347 | FJ793348 | FJ793349 | FJ793350 | FJ793351 | FJ793352 |
| A/chicken/Hunan/2536/2005(H9N2) | CY023773 | CY023774 | CY023775 | CY023776 | CY023777 | CY023778 | CY023779 | [CY023780](http://www.ncbi.nlm.nih.gov/entrez/viewer.fcgi?val=CY023780) |
| A/chicken/Hunan/2903/2005(H9N2) | CY023781 | CY023782 | CY023783 | CY023784 | [CY023785](http://www.ncbi.nlm.nih.gov/entrez/viewer.fcgi?val=CY023785) | CY023786 | CY023787 | [CY023788](http://www.ncbi.nlm.nih.gov/entrez/viewer.fcgi?val=CY023788) |
| A/chicken/Hunan/3369/2005(H9N2) | CY023789 | CY023790 | [CY023791](http://www.ncbi.nlm.nih.gov/entrez/viewer.fcgi?val=CY023791) | CY023792 | [CY023793](http://www.ncbi.nlm.nih.gov/entrez/viewer.fcgi?val=CY023793) | CY023793 | CY023795 | [CY023796](http://www.ncbi.nlm.nih.gov/entrez/viewer.fcgi?val=CY023796) |
| A/chicken/Hunan/4246/2005(H9N2) | CY023805 | CY023806 | CY023807 | CY023808 | CY023809 | CY023810 | CY023811 | CY023812 |
| A/chicken/Hunan/4444/2005(H9N2) | CY023813 | CY023814 | CY023815 | CY023816 | CY023817 | CY023818 | CY023819 | CY023820 |
| A/chicken/Hunan/5260/2005(H9N2) | CY023821 | CY023822 | [CY023823](http://www.ncbi.nlm.nih.gov/entrez/viewer.fcgi?val=CY023823) | CY023824 | CY023825 | CY023826 | CY023827 | CY023828 |
| A/chicken/Hunan/5700/2005(H9N2) | CY023829 | CY023830 | CY023831 | CY023832 | CY023833 | CY023834 | CY023835 | CY023836 |
| A/chicken/Hunan/6108/2005(H9N2) | CY023837 | CY023838 | CY023839 | CY023840 | [CY023841](http://www.ncbi.nlm.nih.gov/entrez/viewer.fcgi?val=CY023841) | CY023842 | CY023843 | CY023844 |
| A/chicken/Israel/282/2005(H9N2) | EF492404 | EF492375 | EF492346 | EF492225 | EF492306 | EF492277 | EF492248 | EF492334 |
| A/chicken/Israel/29/2005(H9N2) | EF492401 | EF492372 | EF492343 | EF492222 | EF492303 | EF492274 | EF492245 | EF492332 |
| A/chicken/Israel/554/2005(H9N2) | EF492406 | EF492377 | EF492348 | EF492227 | EF492308 | EF492279 | EF492250 | [EF492336](http://www.ncbi.nlm.nih.gov/entrez/viewer.fcgi?val=EF492336) |
| A/chicken/Israel/793/2005(H9N2) | EF492411 | EF492382 | EF492353 | EF492232 | EF492313 | EF492284 | EF492253 | [EF492339](http://www.ncbi.nlm.nih.gov/entrez/viewer.fcgi?val=EF492339) |
| A/chicken/Israel/853/2005(H9N2) | EF492413 | EF492384 | EF492355 | EF492234 | EF492315 | EF492286 | [EF492254](http://www.ncbi.nlm.nih.gov/entrez/viewer.fcgi?val=EF492254) | EF492340 |
| A/chicken/Israel/909/2005(H9N2) | EF492415 | EF492386 | EF492357 | EF492236 | EF492317 | EF492288 | EF492256 | EF492331 |
| A/chicken/Pakistan/UDL-01/2005(H9N2) | CY038407 | CY038408 | CY038409 | CY038410 | CY038411 | CY038412 | CY038413 | [CY038414](http://www.ncbi.nlm.nih.gov/entrez/viewer.fcgi?val=CY038414) |
| A/chicken/Pakistan/UDL-02/2005(H9N2) | CY038415 | CY038416 | CY038417 | CY038418 | CY038419 | CY038420 | CY038421 | [CY038422](http://www.ncbi.nlm.nih.gov/entrez/viewer.fcgi?val=CY038422) |
| A/chicken/Shantou/14907/2005(H9N2) | CY023485 | CY023486 | CY023487 | CY023488 | CY023489 | CY023490 | CY023491 | CY023492 |
| A/chicken/Shantou/17139/2005(H9N2) | CY023493 | CY023494 | CY023495 | CY023496 | CY023497 | CY023498 | CY023499 | CY023500 |
| A/chicken/Shantou/19465/2005(H9N2) | CY023501 | CY023502 | CY023503 | CY023504 | CY023505 | CY023506 | CY023507 | CY023508 |
| A/chicken/Shantou/20817/2005(H9N2) | CY023509 | CY023510 | CY023511 | CY023512 | CY023513 | CY023514 | CY023515 | CY023516 |
| A/chicken/Shantou/22054/2005(H9N2) | CY023517 | CY023518 | CY023519 | CY023520 | CY023521 | CY023522 | CY023523 | CY023524 |
| A/chicken/Shantou/22504/2005(H9N2) | CY023525 | CY023526 | CY023527 | CY023528 | CY023529 | CY023530 | CY023531 | CY023532 |
| A/chicken/Shantou/5269/2005(H9N2) | CY023461 | CY023462 | CY023463 | CY023464 | CY023465 | CY023466 | CY023467 | CY023468 |
| A/chicken/Shantou/55/2005(H9N2) | CY023413 | CY023414 | CY023415 | CY023416 | CY023417 | CY023418 | [CY023419](http://www.ncbi.nlm.nih.gov/entrez/viewer.fcgi?val=CY023419) | CY023420 |
| A/chicken/Shantou/6781/2005(H9N2) | CY023469 | CY023470 | [CY023471](http://www.ncbi.nlm.nih.gov/entrez/viewer.fcgi?val=CY023471) | CY023472 | CY023473 | CY023474 | [CY023475](http://www.ncbi.nlm.nih.gov/entrez/viewer.fcgi?val=CY023475) | CY023476 |
| A/chicken/Shantou/9909/2005(H9N2) | CY023477 | CY023478 | CY023479 | CY023480 | CY023481 | CY023482 | CY023483 | CY023484 |
| A/chicken/Hebei/L1/2006(H9N2) | EU914201 | EU882861 | EU532061 | EU573941 | EU532044 | EU346941 | EU532036 | EU532055 |
| A/chicken/Israel/1525/2006(H9N2) | FJ464711 | FJ464694 | FJ464677 | FJ464728 | FJ464644 | FJ464627 | [FJ464610](http://www.ncbi.nlm.nih.gov/entrez/viewer.fcgi?val=FJ464610) | FJ464661 |
| A/chicken/Israel/1548/2006(H9N2) | FJ464712 | FJ464695 | FJ464678 | FJ464729 | FJ464645 | FJ464628 | [FJ464611](http://www.ncbi.nlm.nih.gov/entrez/viewer.fcgi?val=FJ464611) | FJ464662 |
| A/chicken/Israel/1638/2006(H9N2) | FJ464697 | FJ464680 | FJ464664 | FJ464714 | FJ464630 | FJ464613 | FJ464596 | FJ464647 |
| A/chicken/Israel/178/2006(H9N2) | EF492403 | EF492374 | EF492345 | EF492224 | EF492305 | EF492276 | EF492247 | EF492333 |
| A/chicken/Pakistan/UDL-01/2006(H9N2) | CY038423 | CY038424 | CY038425 | CY038426 | CY038427 | CY038428 | CY038429 | CY038430 |
| A/chicken/Pakistan/UDL-02/2006(H9N2) | CY038431 | CY038432 | CY038433 | CY038434 | CY038435 | CY038436 | CY038437 | CY038438 |
| A/chicken/Pakistan/UDL-04/2006(H9N2) | CY038399 | CY038400 | CY038401 | CY038402 | CY038403 | CY038404 | CY038405 | CY038406 |
| A/chicken/Hubei/C1/2007(H9N2) | EU365368 | EU365369 | EU365370 | EU365371 | EU365372 | EU365373 | EU365374 | EU365375 |
| A/chicken/Israel/1033/2007(H9N2) | FJ464710 | FJ464693 | FJ464676 | FJ464727 | FJ464643 | FJ464626 | [FJ464609](http://www.ncbi.nlm.nih.gov/entrez/viewer.fcgi?val=FJ464609) | FJ464660 |
| A/chicken/Israel/1040/2007(H9N2) | GQ140263 | GQ148862 | GQ140277 | GQ120561 | GQ148834 | GQ120534 | GQ148820 | GQ148848 |
| A/chicken/Israel/215/2007(H9N2) | FJ464699 | FJ464682 | FJ464665 | FJ464716 | FJ464632 | FJ464615 | FJ464598 | FJ464649 |
| A/chicken/Israel/375/2007(H9N2) | GQ140269 | GQ148868 | GQ140283 | GQ120553 | GQ148840 | GQ120540 | GQ148826 | GQ148854 |
| A/chicken/Israel/386/2007(H9N2) | FJ464703 | FJ464686 | FJ464669 | FJ464720 | FJ464636 | FJ464619 | FJ464602 | FJ464653 |
| A/chicken/Israel/402/2007(H9N2) | FJ464704 | FJ464687 | FJ464670 | FJ464721 | FJ464637 | FJ464620 | FJ464603 | FJ464654 |
| A/chicken/Israel/449/2007(H9N2) | FJ464705 | FJ464688 | FJ464671 | FJ464722 | FJ464638 | FJ464621 | [FJ464604](http://www.ncbi.nlm.nih.gov/entrez/viewer.fcgi?val=FJ464604) | FJ464655 |
| A/chicken/Israel/728/2007(H9N2) | FJ464707 | FJ464690 | FJ464673 | FJ464724 | FJ464640 | FJ464623 | FJ464606 | FJ464657 |
| A/chicken/Israel/869/2007(H9N2) | GQ140273 | GQ148872 | GQ140287 | GQ120557 | GQ148844 | GQ120544 | GQ148830 | GQ148858 |
| A/chicken/Israel/933/2007(H9N2) | FJ464709 | FJ464692 | FJ464675 | FJ464726 | FJ464642 | FJ464625 | FJ464608 | FJ464659 |
| A/chicken/Israel/951/2007(H9N2) | GQ140275 | GQ148874 | GQ140289 | GQ120559 | GQ148846 | GQ120546 | GQ148832 | [GQ148860](http://www.ncbi.nlm.nih.gov/entrez/viewer.fcgi?val=GQ148860) |
| A/chicken/Israel/953/2007(H9N2) | GQ140276 | GQ148875 | GQ140290 | GQ120560 | GQ148847 | GQ120547 | GQ148833 | GQ148861 |
| A/chicken/Pakistan/UDL-01/2007(H9N2) | CY038391 | CY038392 | CY038393 | CY038394 | CY038395 | CY038396 | CY038397 | CY038398 |
| A/chicken/Pakistan/UDL-03/2007(H9N2) | CY038447 | CY038448 | CY038449 | CY038450 | CY038451 | CY038452 | CY038453 | [CY038454](http://www.ncbi.nlm.nih.gov/entrez/viewer.fcgi?val=CY038454) |
| A/chicken/Pakistan/UDL-04/2007(H9N2) | CY038479 | CY038480 | CY038481 | CY038482 | CY038483 | CY038484 | CY038485 | CY038486 |
| A/chicken/Shandong/B2/2007(H9N2) | EU914194 | EU939157 | EU532062 | EU935071 | EU414523 | EU340028 | EU414522 | [EU532045](http://www.ncbi.nlm.nih.gov/entrez/viewer.fcgi?val=EU532045) |
| A/chicken/Shandong/B3/2007(H9N2) | FJ547482 | EU882863 | EU935067 | EU939143 | EU414524 | EU346933 | EU532028 | EU532046 |
| A/chicken/Shandong/B4/2007(H9N2) | FJ547486 | EU939155 | EU532059 | EU939144 | EU532042 | EU346938 | EU532033 | EU532052 |
| A/chicken/Shanghai/Y2/2007(H9N2) | GQ335504 | [GQ335505](http://www.ncbi.nlm.nih.gov/entrez/viewer.fcgi?val=GQ335505) | GQ335506 | GQ335503 | GQ335507 | GQ335508 | GQ335509 | GQ335510 |
| A/chicken/Zhejiang/Hj/2007(H9N2) | FJ581429 | FJ581435 | FJ581430 | FJ581431 | FJ581428 | FJ581434 | FJ581432 | FJ581433 |
| A/chicken/Henan/L3/2008(H9N2) | FJ492970 | FJ492969 | FJ492967 | FJ547479 | FJ492974 | EU935064 | EU835750 | EU835744 |
| A/chicken/Israel/182/2008(H9N2) | GQ140265 | GQ148864 | GQ140279 | GQ120549 | GQ148836 | / | GQ148822 | GQ148850 |
| A/chicken/Israel/292/2008(H9N2) | FJ464700 | FJ464683 | FJ464666 | FJ464717 | FJ464633 | FJ464616 | FJ464599 | [FJ464650](http://www.ncbi.nlm.nih.gov/entrez/viewer.fcgi?val=FJ464650) |
| A/chicken/Israel/310/2008(H9N2) | GQ140267 | GQ148866 | GQ140281 | GQ120551 | GQ148838 | GQ120538 | GQ148824 | [GQ148852](http://www.ncbi.nlm.nih.gov/entrez/viewer.fcgi?val=GQ148852) |
| A/chicken/Israel/330/2008(H9N2) | GQ140268 | GQ148867 | GQ140282 | GQ120552 | GQ148839 | GQ120539 | GQ148825 | GQ148853 |
| A/chicken/Israel/524/2008(H9N2) | FJ464706 | FJ464689 | FJ464672 | FJ464723 | FJ464639 | FJ464622 | FJ464605 | FJ464656 |
| A/chicken/Israel/54/2008(H9N2) | GQ140264 | GQ148863 | GQ140278 | GQ120548 | GQ148835 | GQ120535 | GQ148821 | GQ148849 |
| A/chicken/Israel/694/2008(H9N2) | GQ140271 | GQ148870 | GQ140285 | GQ120555 | GQ148842 | GQ120542 | GQ148828 | GQ148856 |
| A/chicken/Israel/702/2008(H9N2) | GQ140272 | GQ148871 | GQ140286 | GQ120556 | GQ148843 | GQ120543 | GQ148829 | GQ148857 |
| A/chicken/Israel/883/2008(H9N2) | GQ140274 | GQ148873 | GQ140288 | GQ120558 | GQ148845 | GQ120545 | GQ148831 | GQ148859 |
| A/chicken/Pakistan/UDL-01/2008(H9N2) | CY038455 | CY038456 | CY038457 | CY038458 | CY038459 | CY038460 | CY038461 | CY038462 |
| A/chicken/Pakistan/UDL-02/2008(H9N2) | CY038463 | CY038464 | CY038465 | CY038466 | CY038467 | CY038468 | CY038469 | [CY038470](http://www.ncbi.nlm.nih.gov/entrez/viewer.fcgi?val=CY038470) |
| A/chicken/Pakistan/UDL-03/2008(H9N2) | CY038471 | CY038472 | CY038473 | CY038474 | CY038475 | CY038476 | CY038477 | CY038478 |
| A/chicken/Israel/184/2009(H9N2) | GQ140266 | GQ148865 | GQ140280 | GQ120550 | GQ148837 | GQ120537 | GQ148823 | GQ148851 |
| A/chukkar/Shantou/1059/2002(H9N2) | CY023981 | CY023982 | CY023983 | CY023984 | CY023985 | CY023986 | [CY023987](http://www.ncbi.nlm.nih.gov/entrez/viewer.fcgi?val=CY023987) | [CY023988](http://www.ncbi.nlm.nih.gov/entrez/viewer.fcgi?val=CY023988) |
| A/chukkar/Shantou/338/2002(H9N2) | CY023533 | CY023534 | [CY023535](http://www.ncbi.nlm.nih.gov/entrez/viewer.fcgi?val=CY023535) | CY023536 | CY023537 | CY023538 | CY023539 | [CY023540](http://www.ncbi.nlm.nih.gov/entrez/viewer.fcgi?val=CY023540) |
| A/chukkar/Shantou/465/2002(H9N2) | CY023709 | CY023710 | CY023711 | CY023712 | CY023713 | CY023714 | CY023715 | CY023716 |
| A/chukkar/Shantou/1039/2003(H9N2) | CY024085 | CY024086 | [CY024087](http://www.ncbi.nlm.nih.gov/entrez/viewer.fcgi?val=CY024087) | CY024088 | CY024089 | CY024090 | CY024091 | CY024092 |
| A/chukkar/Shantou/3980/2003(H9N2) | CY024133 | CY024134 | CY024135 | CY024136 | CY024137 | CY024138 | CY024139 | CY024140 |
| A/chukkar/Shantou/4350/2003(H9N2) | CY024181 | CY024182 | CY024183 | CY024184 | [CY024185](http://www.ncbi.nlm.nih.gov/entrez/viewer.fcgi?val=CY024185) | CY024186 | [CY024187](http://www.ncbi.nlm.nih.gov/entrez/viewer.fcgi?val=CY024187) | CY024188 |
| A/chukkar/Shantou/4635/2003(H9N2) | CY024197 | [CY024198](http://www.ncbi.nlm.nih.gov/entrez/viewer.fcgi?val=CY024198) | [CY024199](http://www.ncbi.nlm.nih.gov/entrez/viewer.fcgi?val=CY024198) | [CY024200](http://www.ncbi.nlm.nih.gov/entrez/viewer.fcgi?val=CY024198) | [CY024201](http://www.ncbi.nlm.nih.gov/entrez/viewer.fcgi?val=CY024198) | [CY024202](http://www.ncbi.nlm.nih.gov/entrez/viewer.fcgi?val=CY024198) | [CY024203](http://www.ncbi.nlm.nih.gov/entrez/viewer.fcgi?val=CY024198) | [CY024204](http://www.ncbi.nlm.nih.gov/entrez/viewer.fcgi?val=CY024198) |
| A/chukkar/Shantou/1447/2004(H9N2) | CY024285 | CY024286 | CY024287 | CY024288 | [CY024289](http://www.ncbi.nlm.nih.gov/entrez/viewer.fcgi?val=CY024289) | CY024290 | [CY024291](http://www.ncbi.nlm.nih.gov/entrez/viewer.fcgi?val=CY024291) | CY024292 |
| A/chukkar/Shantou/2226/2004(H9N2) | CY024309 | CY024310 | CY024311 | CY024312 | CY024313 | [CY024314](http://www.ncbi.nlm.nih.gov/entrez/viewer.fcgi?val=CY024314) | CY024315 | CY024316 |
| A/chukkar/Shantou/5866/2004(H9N2) | CY024349 | [CY024350](http://www.ncbi.nlm.nih.gov/entrez/viewer.fcgi?val=CY024350) | CY024351 | CY024352 | CY024353 | CY024354 | CY024355 | CY024356 |
| A/chukkar/Shantou/6288/2004(H9N2) | CY024373 | CY024374 | [CY024375](http://www.ncbi.nlm.nih.gov/entrez/viewer.fcgi?val=CY024375) | CY024376 | CY024377 | [CY024378](http://www.ncbi.nlm.nih.gov/entrez/viewer.fcgi?val=CY024378) | [CY024379](http://www.ncbi.nlm.nih.gov/entrez/viewer.fcgi?val=CY024379) | [CY024380](http://www.ncbi.nlm.nih.gov/entrez/viewer.fcgi?val=CY024380) |
| A/chukkar/Shantou/6571/2004(H9N2) | CY024397 | CY024398 | CY024399 | CY024400 | CY024401 | CY024402 | CY024403 | CY024404 |
| A/chukkar/Shantou/22116/2005(H9N2) | CY024549 | CY024550 | CY024551 | CY024552 | CY024553 | CY024554 | CY024555 | CY024556 |
| A/CK/HK/WF126/2003(H9N2) | DQ226176 | [DQ226165](http://www.ncbi.nlm.nih.gov/entrez/viewer.fcgi?val=DQ226165) | DQ226154 | [AY664671](http://www.ncbi.nlm.nih.gov/entrez/viewer.fcgi?val=AY664671) | AY664728 | AY664709 | AY664690 | AY664747 |
| A/CK/HK/NT142/2003(H9N2) | DQ226175 | [DQ226164](http://www.ncbi.nlm.nih.gov/entrez/viewer.fcgi?val=DQ226164) | DQ226153 | AY664667 | AY664724 | AY664705 | AY664686 | AY664743 |
| A/CK/HK/CSW161/2003(H9N2) | DQ226181 | DQ226170 | DQ226159 | DQ226115 | DQ226148 | DQ226137 | DQ226104 | DQ226126 |
| A/CK/HK/YU463/2003(H9N2) | DQ226178 | DQ226167 | DQ226156 | DQ226112 | DQ226145 | DQ226134 | DQ226101 | DQ226123 |
| A/CK/HK/YU577/2003(H9N2) | DQ226179 | DQ226168 | DQ226157 | DQ226113 | DQ226146 | DQ226135 | DQ226102 | DQ226124 |
| A/duck/Hongkong/86/1976(H9N2) | AF523469 | AF523439 | AF523459 | AF523386 | AF523423 | AF523407 | AF523497 | AF523518 |
| A/duck/Hongkong/289/1978(H9N2) | AF523478 | AF523440 | AF523457 | AF523384 | AF523421 | AF523406 | AF523496 | AF523519 |
| A/duck/Hongkong/366/1978(H9N2) | AF523467 | AF523437 | AF523460 | AY206674 | AF523424 | AF523405 | AF523498 | AF523503 |
| A/duck/Hk/784/1979(H9N2) | CY005638 | CY005637 | CY005636 | CY005632 | CY005634 | / | CY005633 | CY005635 |
| A/duck/Hongkong/552/1979(H9N2) | AF523468 | AF523438 | / | AY206679 | AF523422 | AF523404 | AF523495 | AF523505 |
| A/duck/Hongkong/702/1979(H9N2) | CY031256 | CY031257 | CY031258 | CY031259 | CY031260 | CY031261 | CY031262 | CY031263 |
| A/duck/Hongkong/702/1979(H9N2)-chicken adapted | CY031264 | CY031265 | CY031266 | CY031267 | CY031268 | CY031269 | CY031270 | CY031271 |
| A/duck/Hongkong/702/1979(H9N2)-quail adapted | CY031272 | CY031273 | CY031274 | CY031275 | CY031276 | CY031277 | CY031278 | [CY031279](http://www.ncbi.nlm.nih.gov/entrez/viewer.fcgi?val=CY031279) |
| A/duck/Altai/1285/1991(H5N3) | GQ227557 | [GQ227558](http://www.ncbi.nlm.nih.gov/entrez/viewer.fcgi?val=GQ227558) | [GQ227554](http://www.ncbi.nlm.nih.gov/entrez/viewer.fcgi?val=GQ227554) | GQ227551 | GQ227553 | GQ227552 | GQ227555 | GQ227556 |
| A/Duck/Germany/113/1995(H9N2) | AF508644 | AF508622 | AF508666 | AF508557 | AF508600 | AF508578 | AF508688 | AF508709 |
| A/duck/Hong kong/Y280/1997(H9N2) | AF156433 | AF156419 | AF156447 | AF156376 | AF156405 | AF156394 | AF156461 | AF156475 |
| A/duck/Hongkong/Y439/1997(H9N2) | AF156434 | AF156420 | AF156448 | AF156377 | AF156406 | AF156395 | AF156462 | AF156476 |
| A/duck/Nanjing/1/1997(H9N2) | DQ064562 | AF508638 | DQ064508 | DQ064373 | DQ064454 | DQ064427 | DQ064400 | DQ064481 |
| A/duck/Nanjing/2/1997(H9N2) | DQ064563 | DQ064536 | DQ064509 | DQ064374 | DQ064455 | DQ064428 | [DQ064401](http://www.ncbi.nlm.nih.gov/entrez/viewer.fcgi?val=DQ064401) | DQ064482 |
| A/duck/Hokkaido/49/1998(H9N2) | AB473937 | AB473938 | AB473939 | AB125928 | AB473940 | AB251944 | AB473941 | AB473942 |
| A/duck/Hokkaido/9/1999(H9N2) | AB262460 | AB262461 | AB262462 | AB262463 | AB262464 | AB262465 | AB262466 | AB262467 |
| A/duck/Guangdong/40/2000(H5N1) | AY585512 | [AY585491](http://www.ncbi.nlm.nih.gov/entrez/viewer.fcgi?val=AY585491) | [AY585470](http://www.ncbi.nlm.nih.gov/entrez/viewer.fcgi?val=AY585470) | AY585374 | AY585428 | AY585407 | AY585386 | [AY585449](http://www.ncbi.nlm.nih.gov/entrez/viewer.fcgi?val=AY585449) |
| A/duck/Hokkaido/447/2000(H5N3) | AB300437 | [AB300227](http://www.ncbi.nlm.nih.gov/entrez/viewer.fcgi?val=AB300227) | [AB300228](http://www.ncbi.nlm.nih.gov/entrez/viewer.fcgi?val=AB300228) | AB241620 | AB300229 | AB300230 | AB300231 | AB300232 |
| A/Duck/Nanchang/8-174/2000 (H3N6) | AY180739 | AY180853 | AY180659 | AY180422 | AY180547 | AY180799 | AY180473 | AY180590 |
| A/duck/Shantou/1042/2000(H9N2) | AF523466 | AF523443 | AF523447 | AF523383 | AF523413 | AF523391 | AF523485 | AF523516 |
| A/duck/Shantou/1043/2000(H9N2) | AF523465 | AF523429 | AF523446 | AF523382 | AF523410 | AF523392 | AF523482 | AF523513 |
| A/duck/Shantou/1881/2000(H9N2) | AF523479 | AF523436 | AF523455 | AF523373 | AF523420 | AF523398 | AF523488 | AF523507 |
| A/duck/Shantou/2102/2000(H9N2) | AF523472 | AF523428 | AF523456 | AF523378 | AF523415 | AF523396 | AF523490 | AF523511 |
| A/duck/Shantou/2134/2000(H9N2) | AF523463 | AF523430 | AF523452 | AF523380 | AF523411 | AF523393 | AF523483 | AF523515 |
| A/duck/Shantou/2143/2000(H9N2) | AF523474 | AF523433 | AF523451 | AF523374 | AF523419 | AF523397 | AF523486 | AF523508 |
| A/duck/Shantou/2144/2000(H9N2) | AF523473 | AF523432 | AF523453 | AF523372 | AF523417 | AF523399 | [AF523487](http://www.ncbi.nlm.nih.gov/entrez/viewer.fcgi?val=AF523487) | AF523506 |
| A/duck/Shantou/830/2000(H9N2) | AF523476 | AF523427 | AF523449 | AF523377 | AF523416 | AF523400 | AF523491 | AF523512 |
| A/duck/Zhejiang/52/2000(H5N1) | AY585524 | [AY585503](http://www.ncbi.nlm.nih.gov/entrez/viewer.fcgi?val=AY585503) | AY585482 | AY585377 | AY585440 | AY585419 | AY585397 | AY585461 |
| A/duck/Fujian/17/2001(H5N1) | AY585506 | [AY585485](http://www.ncbi.nlm.nih.gov/entrez/viewer.fcgi?val=AY585485) | [AY585464](http://www.ncbi.nlm.nih.gov/entrez/viewer.fcgi?val=AY585464) | AY585372 | AY585422 | AY585401 | AY585380 | AY585443 |
| A/duck/Guangxi/35/2001(H5N1) | AY585515 | [AY585494](http://www.ncbi.nlm.nih.gov/entrez/viewer.fcgi?val=AY585494) | [AY585473](http://www.ncbi.nlm.nih.gov/entrez/viewer.fcgi?val=AY585473) | AY585365 | AY585431 | AY585410 | AY585389 | AY585452 |
| A/duck/Guangxi/50/2001(H5N1) | AY585516 | [AY585495](http://www.ncbi.nlm.nih.gov/entrez/viewer.fcgi?val=AY585495) | [AY585474](http://www.ncbi.nlm.nih.gov/entrez/viewer.fcgi?val=AY585474) | AY585375 | AY585432 | AY585411 | AY585398 | AY585453 |
| A/duck/Guangxi/xa/2001(H5N1) | DQ997519 | [DQ997518](http://www.ncbi.nlm.nih.gov/entrez/viewer.fcgi?val=DQ997518) | [DQ997517](http://www.ncbi.nlm.nih.gov/entrez/viewer.fcgi?val=DQ997517) | DQ997513 | DQ997515 | DQ997514 | DQ997520 | DQ997516 |
| A/duck/Mongolia/54/2001(H5N2) | AB301913 | [AB302086](http://www.ncbi.nlm.nih.gov/entrez/viewer.fcgi?val=AB302086) | [AB301914](http://www.ncbi.nlm.nih.gov/entrez/viewer.fcgi?val=AB301914) | AB241614 | AB301915 | AB298276 | AB301916 | AB301917 |
| A/duck/Nanchang/4-361/2001(H9N2) | CY005530 | CY005529 | CY005528 | CY006025 | CY005527 | CY005526 | CY005525 | / |
| A/duck/Shantou/2088/2001(H9N2) | AF523475 | AF523435 | AF523448 | AF523376 | AF523418 | AF523395 | AF523492 | AF523510 |
| A/duck/Shantou/3549/2001(H9N2) | CY023909 | CY023910 | CY023911 | CY023912 | CY023913 | CY023914 | CY023915 | CY023916 |
| A/duck/Shantou/5401/2001(H9N2) | CY023917 | CY023918 | CY023919 | CY023920 | CY023921 | CY023922 | CY023923 | CY023924 |
| A/duck/Shantou/5459/2001(H9N2) | CY023925 | CY023926 | CY023927 | CY023928 | CY023929 | CY023930 | [CY023931](http://www.ncbi.nlm.nih.gov/entrez/viewer.fcgi?val=CY023931) | CY023932 |
| A/duck/Shantou/5753/2001(H9N2) | CY023933 | CY023934 | CY023935 | CY023936 | [CY023937](http://www.ncbi.nlm.nih.gov/entrez/viewer.fcgi?val=CY023937) | CY023938 | [CY023939](http://www.ncbi.nlm.nih.gov/entrez/viewer.fcgi?val=CY023939) | CY023940 |
| A/duck/Hokkaido/84/2002(H5N3) | AB300223 | [AB300224](http://www.ncbi.nlm.nih.gov/entrez/viewer.fcgi?val=AB300224) | [AB300435](http://www.ncbi.nlm.nih.gov/entrez/viewer.fcgi?val=AB300435) | AB241623 | AB300436 | AB299161 | AB300225 | AB300226 |
| A/duck/jiangsu/nf/2002(H9N2) | DQ997450 | DQ997449 | DQ997455 | DQ997451 | DQ997453 | DQ997452 | DQ997456 | DQ997454 |
| A/duck/Shanghai/35/2002(H5N1) | AY585520 | [AY585499](http://www.ncbi.nlm.nih.gov/entrez/viewer.fcgi?val=AY585499) | [AY585478](http://www.ncbi.nlm.nih.gov/entrez/viewer.fcgi?val=AY585478) | AY585368 | AY585436 | AY585415 | AY585393 | AY585457 |
| A/duck/Shantou/32/2002(H9N2) | CY023941 | CY023942 | CY023943 | CY023944 | [CY023945](http://www.ncbi.nlm.nih.gov/entrez/viewer.fcgi?val=CY023945) | CY023946 | CY023947 | [CY023948](http://www.ncbi.nlm.nih.gov/entrez/viewer.fcgi?val=CY023948) |
| A/duck/Shantou/4103/2002(H9N2) | CY023949 | CY023950 | CY023951 | CY023952 | CY023953 | CY023954 | CY023955 | CY023956 |
| A/duck/Shantou/4359/2002(H9N2) | CY023957 | CY023958 | CY023959 | CY023960 | CY023961 | CY023962 | CY023963 | CY023964 |
| A/duck/Zhejiang/3/2002(H9N2) | FJ793297 | FJ793298 | FJ793299 | FJ793300 | FJ793301 | FJ793302 | FJ793303 | FJ793304 |
| A/duck/Korea/S13/2003(H9N2) | AY862714 | AY862698 | [AY862682](http://www.ncbi.nlm.nih.gov/entrez/viewer.fcgi?val=AY862682) | AY862602 | AY862650 | AY862634 | AY862618 | AY862666 |
| A/duck/Shantou/3460/2003(H9N2) | CY023965 | CY023966 | CY023967 | CY023968 | CY023969 | CY023970 | CY023971 | CY023972 |
| A/duck/Shantou/3577/2003(H9N2) | CY023989 | CY023990 | CY023991 | CY023992 | [CY023993](http://www.ncbi.nlm.nih.gov/entrez/viewer.fcgi?val=CY023993) | [CY023994](http://www.ncbi.nlm.nih.gov/entrez/viewer.fcgi?val=CY023994) | [CY023995](http://www.ncbi.nlm.nih.gov/entrez/viewer.fcgi?val=CY023995) | CY023996 |
| A/duck/Shantou/3658/2003(H9N2) | CY023997 | CY023998 | CY023999 | CY024000 | CY024001 | CY024002 | CY024003 | CY024004 |
| A/duck/Shantou/3728/2003(H9N2) | CY024005 | CY024006 | CY024007 | CY024008 | [CY024009](http://www.ncbi.nlm.nih.gov/entrez/viewer.fcgi?val=CY024009) | [CY024010](http://www.ncbi.nlm.nih.gov/entrez/viewer.fcgi?val=CY024010) | [CY024011](http://www.ncbi.nlm.nih.gov/entrez/viewer.fcgi?val=CY024011) | CY024012 |
| A/duck/Denmark/65047/04(H5N2) | DQ251449 | DQ251450 | DQ251451 | DQ251447 | DQ251452 | DQ251448 | DQ251453 | DQ251454 |
| A/duck/Hubei/WI/2004(H9N2) | DQ465397 | DQ465398 | DQ465399 | DQ465400 | DQ465401 | DQ465402 | DQ465403 | DQ465404 |
| A/duck/Shantou/163/2004(H9N2) | CY024013 | CY024014 | CY024015 | CY024016 | CY024017 | CY024018 | CY024019 | CY024020 |
| A/duck/Shantou/515/2004(H9N2) | CY024021 | CY024022 | CY024023 | CY024024 | CY024025 | CY024026 | [CY024027](http://www.ncbi.nlm.nih.gov/entrez/viewer.fcgi?val=CY024027) | CY024028 |
| A/duck/Shantou/7488/2004(H9N2) | CY024029 | CY024030 | CY024031 | CY024032 | CY024033 | CY024034 | CY024035 | CY024036 |
| A/duck/Guangxi/51/2005(H9N2) | EU086262 | EU086260 | EU086259 | EU086234 | EU086251 | EU086236 | EU086235 | EU086254 |
| A/duck/Jiangsu/3/2005(H9N2) | FJ793281 | FJ793282 | FJ793283 | FJ793284 | FJ793285 | FJ793286 | FJ793287 | FJ793288 |
| A/duck/Shantou/12560/2005(H9N2) | CY024037 | CY024038 | CY024039 | CY024040 | [CY024041](http://www.ncbi.nlm.nih.gov/entrez/viewer.fcgi?val=CY024041) | CY024042 | CY024043 | CY024044 |
| A/Eurasian wigeon/Netherlands/3/2005(H9N2) | CY043863 | CY043862 | CY043861 | CY043856 | CY043859 | CY043858 | CY043857 | CY043860 |
| A/Gadwall/Netherlands/1/2006(H9N2) | CY043871 | DQ226171 | CY043869 | CY043864 | CY043867 | CY043866 | CY043865 | CY043868 |
| A/Gf/HK/NT101/2003(H9N2) | DQ226182 | DQ226163 | DQ226160 | DQ226116 | DQ226149 | DQ226138 | DQ226105 | DQ226127 |
| A/Gf/HK/SSP607/2003(H9N2) | DQ226174 | [DQ226163](http://www.ncbi.nlm.nih.gov/entrez/viewer.fcgi?val=DQ226163) | DQ226152 | DQ226108 | DQ226141 | DQ226130 | DQ226097 | DQ226119 |
| A/goose/MN/5733-1/1980(H9N2) | CY005880 | CY005879 | CY005878 | CY006042 | CY005876 | CY005875 | CY005874 | CY005877 |
| A/Goose/Guangdong/1/96(H5N1) | AF144300 | [AF144301](http://www.ncbi.nlm.nih.gov/entrez/viewer.fcgi?val=AF144300) | [AF144302](http://www.ncbi.nlm.nih.gov/entrez/viewer.fcgi?val=AF144300) | AF144305 | AF144303 | AF144304 | AF144306 | AF144307 |
| A/Guinea fowl/Shantou/1677/2000(H9N2) | CY023973 | CY023974 | CY023975 | CY023976 | [CY023977](http://www.ncbi.nlm.nih.gov/entrez/viewer.fcgi?val=CY023977) | CY023978 | CY023979 | CY023980 |
| A/Guinea fowl/Shantou/2076/2001(H9N2) | CY024557 | CY024558 | CY024559 | CY024560 | CY024561 | CY024562 | CY024563 | CY024564 |
| A/Guinea fowl/Shantou/5852/2004(H9N2) | CY024341 | CY024342 | CY024343 | CY024344 | CY024345 | CY024346 | CY024347 | [CY024348](http://www.ncbi.nlm.nih.gov/entrez/viewer.fcgi?val=CY024348) |
| A/Guinea fowl/Shantou/630/2005(H9N2) | CY024493 | CY024494 | CY024495 | CY024496 | CY024497 | CY024498 | [CY024499](http://www.ncbi.nlm.nih.gov/entrez/viewer.fcgi?val=CY024499) | CY024500 |
| A/Guinea fowl/Shantou/8955/2005(H9N2) | CY024509 | CY024510 | CY024511 | CY024512 | CY024513 | CY024514 | [CY024515](http://www.ncbi.nlm.nih.gov/entrez/viewer.fcgi?val=CY024515) | CY024516 |
| A/guineafowl/HongKong/NT184/2003(H9N2) | AY664806 | [AY664788](http://www.ncbi.nlm.nih.gov/entrez/viewer.fcgi?val=AY664788) | AY664769 | AY664674 | AY664731 | AY664712 | AY664693 | AY664750 |
| A/laughing gull/Delaware/12/2006(H9N2) | CY041433 | CY041432 | CY041431 | CY041426 | CY041429 | CY041428 | CY041427 | CY041430 |
| A/mallard/ALB/17/1991(H9N2) | AY633171 | AY633170 | CY005158 | CY005991 | CY005156 | CY005155 | AY633165 | AY633168 |
| A/mallard/Alberta/11/1991(H9N2) | CY005153 | CY005152 | CY005151 | CY005990 | CY005149 | CY014591 | CY005148 | CY005150 |
| A/mallard/Netherlands/12/2000(H7N3) | CY005851 | [CY005850](http://www.ncbi.nlm.nih.gov/entrez/viewer.fcgi?val=CY005850) | [CY005849](http://www.ncbi.nlm.nih.gov/entrez/viewer.fcgi?val=CY005849) | CY014718 | CY005847 | CY005846 | CY005845 | CY005848 |
| A/mallard/Italy/33/01(H7N3) | AY586440 | [AY586437](http://www.ncbi.nlm.nih.gov/entrez/viewer.fcgi?val=AY586437) | [AY586432](http://www.ncbi.nlm.nih.gov/entrez/viewer.fcgi?val=AY586432) | AY586411 | AY586423 | AY586415 | AY586430 | AY586442 |
| A/mallard/Italy/43/01(H7N3) | AY586445 | [AY586438](http://www.ncbi.nlm.nih.gov/entrez/viewer.fcgi?val=AY586438) | [AY586431](http://www.ncbi.nlm.nih.gov/entrez/viewer.fcgi?val=AY586431) | AY586410 | AY586424 | AY586416 | AY586429 | AY586443 |
| A/mallard/Sweden/S90735/2003(H7N7) | FJ803184 | [FJ803185](http://www.ncbi.nlm.nih.gov/entrez/viewer.fcgi?val=FJ803185) | [FJ803186](http://www.ncbi.nlm.nih.gov/entrez/viewer.fcgi?val=FJ803186) | FJ803190 | FJ803188 | FJ803183 | FJ803189 | FJ803187 |
| A/mallard/Netherlands/2/2005(H4N2) | CY041257 | CY041256 | [CY041255](http://www.ncbi.nlm.nih.gov/entrez/viewer.fcgi?val=CY041255) | CY041250 | CY041253 | CY041252 | CY041251 | CY041254 |
| A/mallard/Netherlands/3/2005(H3N8) | CY041249 | CY041248 | [CY041247](http://www.ncbi.nlm.nih.gov/entrez/viewer.fcgi?val=CY041247) | CY041242 | CY041245 | CY041244 | CY041243 | CY041246 |
| A/mallard/Sweden/4/2005(H10N4) | CY043879 | CY043878 | CY043877 | CY043872 | CY043875 | CY043874 | CY043873 | CY043876 |
| A/mallard/Sweden/65/2005(H4N3) | CY041361 | CY041360 | [CY041359](http://www.ncbi.nlm.nih.gov/entrez/viewer.fcgi?val=CY041359) | CY041354 | CY041357 | CY041356 | CY041355 | CY041358 |
| A/mallard duck/AST/266/1982(H14N5) | / | CY005401 | CY005400 | / | CY005398 | / | / | CY005399 |
| A/mallard duck/AlB/321/1988(H9N2) | CY005147 | [CY005146](http://www.ncbi.nlm.nih.gov/entrez/viewer.fcgi?val=CY005146) | [CY005145](http://www.ncbi.nlm.nih.gov/entrez/viewer.fcgi?val=CY005145) | CY005989 | CY005143 | CY005142 | CY005141 | CY005144 |
| A/migratory duck/Jiang Xi/6568/2004(H4N6) | EF597496 | EF597462 | [EF597427](http://www.ncbi.nlm.nih.gov/entrez/viewer.fcgi?val=EF597427) | / | EF597357 | / | EF597301 | EF597392 |
| A/northern pintail/Alaska/44202-103/2006(H3N8) | / | EU557448 | EU557485 | EU557508 | EU557558 | EU557592 | EU557631 | EU557669 |
| A/ostrich/South Africa/9508103/1995(H9N2) | AF508640 | AF508618 | AF508662 | AF508554 | AF508596 | AF508575 | AF508684 | AF508705 |
| A/ostrich/Israel/1436/2003(H9N2) | EF492421 | EF492392 | EF492363 | AY738456 | EF492323 | EF492294 | EF492261 | DQ683040 |
| A/Parakeet/Chiba/1/1997(H9N2) | AB049153 | AB049155 | AB049157 | AB049159 | AB049161 | AB049163 | AB049165 | AB049167 |
| A/Parakeet/Narita/92A/1998(H9N2) | AB049154 | AB049156 | AB049158 | AB049160 | AB049162 | AB049164 | AB049166 | AB049168 |
| A/Partridge/Shantou/1800/2000(H9N2) | CY024117 | CY024118 | CY024119 | CY024120 | CY024121 | CY024122 | CY024123 | CY024124 |
| A/Partridge/Shantou/2063/2000(H9N2) | CY024205 | CY024206 | CY024207 | CY024208 | [CY024209](http://www.ncbi.nlm.nih.gov/entrez/viewer.fcgi?val=CY024209) | CY024210 | CY024211 | CY024212 |
| A/Partridge/Shantou/2158/2000(H9N2) | CY024293 | CY024294 | CY024295 | CY024296 | CY024297 | CY024298 | CY024299 | CY024300 |
| A/Partridge/Shantou/24/2000(H9N2) | CY024736 | CY023090 | CY024733 | CY023091 | CY023092 | CY024734 | CY024737 | CY024735 |
| A/Partridge/Shantou/5692/2000(H9N2) | CY024381 | CY024382 | CY024383 | CY024384 | CY024385 | CY024386 | CY024387 | CY024388 |
| A/Partridge/Shantou/2875/2001(H9N2) | CY023093 | CY023094 | CY023095 | CY023096 | [CY023097](http://www.ncbi.nlm.nih.gov/entrez/viewer.fcgi?val=CY023097) | CY023098 | CY023099 | CY023100 |
| A/Partridge/Shantou/3720/2001(H9N2) | CY023181 | CY023182 | CY023183 | CY023184 | CY023185 | CY023186 | CY023187 | CY023188 |
| A/Partridge/Shantou/4541/2001(H9N2) | CY023269 | CY023270 | CY023271 | CY023272 | CY023273 | CY023274 | CY023275 | CY023276 |
| A/Partridge/Shantou/4829/2001(H9N2) | CY023357 | CY023358 | CY023359 | CY023360 | CY023361 | CY023362 | CY023363 | CY023364 |
| A/Partridge/Shantou/3811/2002(H9N2) | CY024045 | CY024046 | CY024047 | CY024048 | CY024049 | CY024050 | CY024051 | CY024052 |
| A/Partridge/Shantou/4525/2002(H9N2) | CY024053 | CY024054 | CY024055 | CY024056 | CY024057 | CY024058 | CY024059 | CY024060 |
| A/Partridge/Shantou/49/2002(H9N2) | CY023445 | CY023446 | CY023447 | CY023448 | CY023449 | CY023450 | CY023451 | CY023452 |
| A/Partridge/Shantou/545/2002(H9N2) | CY023885 | CY023886 | CY023887 | CY023888 | CY023889 | [CY023890](http://www.ncbi.nlm.nih.gov/entrez/viewer.fcgi?val=CY023890) | CY023891 | CY023892 |
| A/Partridge/Shantou/2803/2003(H9N2) | CY024093 | CY024094 | CY024095 | CY024096 | CY024097 | CY024098 | [CY024099](http://www.ncbi.nlm.nih.gov/entrez/viewer.fcgi?val=CY024099) | CY024100 |
| A/Partridge/Shantou/3487/2003(H9N2) | CY024101 | CY024102 | CY024103 | CY024104 | [CY024105](http://www.ncbi.nlm.nih.gov/entrez/viewer.fcgi?val=CY024105) | CY023626 | [CY024107](http://www.ncbi.nlm.nih.gov/entrez/viewer.fcgi?val=CY024107) | [CY024108](http://www.ncbi.nlm.nih.gov/entrez/viewer.fcgi?val=CY024108) |
| A/Partridge/Shantou/3987/2003(H9N2) | CY024141 | CY024142 | CY024143 | CY024144 | CY024145 | CY024146 | CY024147 | CY024148 |
| A/Partridge/Shantou/4093/2003(H9N2) | CY024149 | CY024150 | CY024151 | CY024152 | CY024153 | CY024154 | CY024155 | CY024156 |
| A/partridge/Shantou/4321/2003(H9N2) | CY024165 | CY024166 | CY024167 | CY024168 | CY024169 | CY024170 | CY024171 | CY024172 |
| A/Partridge/Shantou/4889/2003(H9N2) | CY024221 | CY024222 | CY024223 | CY024224 | CY024225 | CY024226 | CY024227 | CY024228 |
| A/Partridge/Shantou/600/2003(H9N2) | CY024077 | CY024078 | CY024079 | CY024080 | [CY024081](http://www.ncbi.nlm.nih.gov/entrez/viewer.fcgi?val=CY024081) | CY024082 | CY024083 | CY024084 |
| A/Partridge/Shantou/1405/2004(H9N2) | CY024277 | CY024278 | [CY024279](http://www.ncbi.nlm.nih.gov/entrez/viewer.fcgi?val=CY024279) | CY024280 | CY024281 | CY024282 | [CY024283](http://www.ncbi.nlm.nih.gov/entrez/viewer.fcgi?val=CY024283) | CY024284 |
| A/Partridge/Shantou/1651/2004(H9N2) | CY024301 | CY024302 | CY024303 | CY024304 | [CY024305](http://www.ncbi.nlm.nih.gov/entrez/viewer.fcgi?val=CY024305) | CY024306 | CY024307 | CY024308 |
| A/Partridge/Shantou/25/2004(H9N2) | CY024229 | CY024230 | CY024231 | CY024232 | CY024233 | CY024234 | CY024235 | CY024236 |
| A/Partridge/Shantou/4648/2004(H9N2) | CY024325 | CY024326 | CY024327 | CY024328 | CY024329 | CY024330 | CY024331 | CY024332 |
| A/Partridge/Shantou/6004/2004(H9N2) | CY024357 | CY024358 | [CY024359](http://www.ncbi.nlm.nih.gov/entrez/viewer.fcgi?val=CY024359) | CY024360 | CY024361 | CY024362 | [CY024363](http://www.ncbi.nlm.nih.gov/entrez/viewer.fcgi?val=CY024363) | [CY024364](http://www.ncbi.nlm.nih.gov/entrez/viewer.fcgi?val=CY024364) |
| A/Partridge/Shantou/6266/2004(H9N2) | CY024469 | CY024366 | CY024367 | CY024368 | CY024369 | CY024370 | CY024371 | CY024372 |
| A/Partridge/Shantou/6415/2004(H9N2) | CY024389 | CY024390 | [CY024391](http://www.ncbi.nlm.nih.gov/entrez/viewer.fcgi?val=CY024391) | CY024392 | CY024393 | CY024474 | CY024395 | [CY024396](http://www.ncbi.nlm.nih.gov/entrez/viewer.fcgi?val=CY024396) |
| A/Partridge/Shantou/688/2004(H9N2) | CY024261 | CY024262 | CY024263 | CY024264 | CY024265 | CY024266 | [CY024267](http://www.ncbi.nlm.nih.gov/entrez/viewer.fcgi?val=CY024267) | [CY024268](http://www.ncbi.nlm.nih.gov/entrez/viewer.fcgi?val=CY024268) |
| A/Partridge/Shantou/7075/2004(H9N2) | CY024429 | CY024430 | CY024431 | CY024432 | CY024433 | CY024434 | CY024435 | CY024436 |
| A/Partridge/Shantou/7343/2004(H9N2) | CY024445 | CY024446 | CY024447 | CY024448 | [CY024449](http://www.ncbi.nlm.nih.gov/entrez/viewer.fcgi?val=CY024449) | [CY024450](http://www.ncbi.nlm.nih.gov/entrez/viewer.fcgi?val=CY024450) | [CY024451](http://www.ncbi.nlm.nih.gov/entrez/viewer.fcgi?val=CY024451) | CY024452 |
| A/Partridge/Shantou/7936/2004(H9N2) | CY024477 | CY024478 | [CY024479](http://www.ncbi.nlm.nih.gov/entrez/viewer.fcgi?val=CY024479) | CY024480 | CY024481 | CY024482 | [CY024483](http://www.ncbi.nlm.nih.gov/entrez/viewer.fcgi?val=CY024483) | CY024484 |
| A/Partridge/Shantou/22102/2005(H9N2) | CY024541 | CY024542 | [CY024543](http://www.ncbi.nlm.nih.gov/entrez/viewer.fcgi?val=CY024543) | CY024544 | [CY024545](http://www.ncbi.nlm.nih.gov/entrez/viewer.fcgi?val=CY024545) | CY024546 | CY024547 | [CY024548](http://www.ncbi.nlm.nih.gov/entrez/viewer.fcgi?val=CY024548) |
| A/Ph/HK/CSW1323/2003(H9N2) | DQ226173 | DQ226162 | DQ226151 | DQ226107 | DQ226140 | DQ226129 | DQ226096 | DQ226118 |
| A/pheasant/Ireland/PV18/1997(H9N2) | AF508647 | AF508625 | AF508669 | AF508560 | AF508603 | AF508581 | AF508691 | AF508712 |
| A/pheasant/HongKong/WF54/2003(H9N2) | AY664805 | AY664787 | AY664768 | AY664673 | AY664730 | AY664711 | AY664692 | [AY664749](http://www.ncbi.nlm.nih.gov/entrez/viewer.fcgi?val=AY664749) |
| A/pheasant/Shantou/3530/2003(H9N2) | CY024109 | CY024110 | CY024111 | CY024112 | [CY024113](http://www.ncbi.nlm.nih.gov/entrez/viewer.fcgi?val=CY024113) | CY024114 | CY024115 | CY024116 |
| A/pheasant/Shantou/3716/2003(H9N2) | CY024125 | CY024126 | CY024127 | CY024128 | CY024129 | CY024130 | CY024131 | CY024132 |
| A/pheasant/Shantou/4116/2003(H9N2) | CY024157 | CY024158 | CY024159 | CY024160 | CY024161 | CY024162 | CY024163 | CY024164 |
| A/pheasant/Shantou/4340/2003(H9N2) | CY024173 | CY024174 | CY024175 | CY024176 | CY024177 | CY024178 | CY024179 | CY024180 |
| A/pheasant/Shantou/443/2003(H9N2) | CY024061 | CY024062 | CY024063 | CY024064 | CY024065 | CY024066 | [CY024067](http://www.ncbi.nlm.nih.gov/entrez/viewer.fcgi?val=CY024067) | CY024068 |
| A/pheasant/Shantou/4469/2003(H9N2) | CY024189 | CY024190 | [CY024191](http://www.ncbi.nlm.nih.gov/entrez/viewer.fcgi?val=CY024191) | CY024192 | [CY024193](http://www.ncbi.nlm.nih.gov/entrez/viewer.fcgi?val=CY024193) | CY024194 | CY024195 | CY024196 |
| A/pheasant/Shantou/4709/2003(H9N2) | CY024213 | CY024214 | CY024215 | CY024216 | CY024217 | CY024218 | CY024219 | CY024220 |
| A/pheasant/Shantou/511/2003(H9N2) | CY024069 | CY024070 | CY024071 | CY024072 | CY024073 | CY024074 | CY024075 | CY024076 |
| A/pheasant/Shantou/2290/2004(H9N2) | CY024317 | CY024318 | [CY024319](http://www.ncbi.nlm.nih.gov/entrez/viewer.fcgi?val=CY024319) | CY024320 | CY024321 | CY024322 | [CY024323](http://www.ncbi.nlm.nih.gov/entrez/viewer.fcgi?val=CY024323) | CY024324 |
| A/pheasant/Shantou/45/2004(H9N2) | CY024237 | CY024238 | CY024239 | CY024240 | CY024241 | CY024242 | CY024243 | CY024244 |
| A/pheasant/Shantou/5588/2004(H9N2) | CY024333 | CY024334 | CY024335 | CY024336 | CY024337 | CY024338 | CY024339 | CY024340 |
| A/pheasant/Shantou/6607/2004(H9N2) | CY024405 | CY024406 | CY024407 | CY024408 | CY024409 | CY024410 | CY024411 | CY024412 |
| A/pheasant/Shantou/6893/2004(H9N2) | CY024421 | CY024422 | CY024423 | CY024424 | [CY024425](http://www.ncbi.nlm.nih.gov/entrez/viewer.fcgi?val=CY024425) | CY024426 | CY024427 | CY024428 |
| A/pheasant/Shantou/7315/2004(H9N2) | CY024437 | CY024438 | CY024439 | CY024440 | CY024441 | [CY024442](http://www.ncbi.nlm.nih.gov/entrez/viewer.fcgi?val=CY024442) | CY024443 | CY024444 |
| A/pheasant/Shantou/7501/2004(H9N2) | CY024453 | CY024454 | CY024455 | CY024456 | CY024457 | [CY024458](http://www.ncbi.nlm.nih.gov/entrez/viewer.fcgi?val=CY024458) | [CY024459](http://www.ncbi.nlm.nih.gov/entrez/viewer.fcgi?val=CY024459) | [CY024460](http://www.ncbi.nlm.nih.gov/entrez/viewer.fcgi?val=CY024460) |
| A/pheasant/Shantou/7814/2004(H9N2) | CY024461 | CY024462 | CY024463 | CY024464 | [CY024465](http://www.ncbi.nlm.nih.gov/entrez/viewer.fcgi?val=CY024465) | CY024466 | CY024467 | CY024468 |
| A/pheasant/Shantou/841/2004(H9N2) | CY024269 | CY024270 | CY024271 | CY024272 | [CY024273](http://www.ncbi.nlm.nih.gov/entrez/viewer.fcgi?val=CY024273) | CY024274 | [CY024275](http://www.ncbi.nlm.nih.gov/entrez/viewer.fcgi?val=CY024275) | [CY024276](http://www.ncbi.nlm.nih.gov/entrez/viewer.fcgi?val=CY024276) |
| A/pheasant/Shantou/111/2005(H9N2) | CY024485 | CY024486 | CY024487 | CY024488 | CY024489 | CY024490 | CY024491 | CY024492 |
| A/pheasant/Shantou/11551/2005(H9N2) | CY024517 | CY024518 | CY024519 | CY024520 | CY024521 | CY024522 | CY024523 | CY024524 |
| A/pheasant/Shantou/1578/2005(H9N2) | CY024501 | CY024502 | [CY024503](http://www.ncbi.nlm.nih.gov/entrez/viewer.fcgi?val=CY024503) | CY024504 | CY024505 | CY024506 | CY024507 | CY024508 |
| A/pheasant/Shantou/17033/2005(H9N2) | CY024525 | CY024526 | CY024527 | CY024528 | CY024529 | CY024530 | CY024531 | [CY024532](http://www.ncbi.nlm.nih.gov/entrez/viewer.fcgi?val=CY024532) |
| A/pheasant/Shantou/21583/2005(H9N2) | CY024533 | CY024534 | CY024535 | CY024536 | [CY024537](http://www.ncbi.nlm.nih.gov/entrez/viewer.fcgi?val=CY024537) | CY024538 | CY024539 | CY024540 |
| A/pigeon/Hongkong/Y233/1997(H9N2) | AF156432 | AF156418 | AF156446 | AF156375 | AF156404 | AF156393 | AF156460 | AF156474 |
| A/pigeon/Hongkong/FY6/1999(H9N2) | AF222623 | AF222633 | AF222643 | AF222607 | AF222615 | AF222653 | AF222663 | AF222673 |
| A/Pigeon/Nanchang/2-0461/2000(H9N2) | CY005512 | CY005511 | CY005510 | CY014613 | CY005509 | CY005508 | CY005507 | CY006020 |
| A/quail/Hongkong/AF157/1992(H9N2) | AF156437 | AF156424 | AF156451 | AF156381 | AF156410 | AF156399 | AF156465 | AF156479 |
| A/quail/Shanghai/8/1996(H9N2) | AF508661 | AF508639 | AF508683 | AF508574 | AF508617 | AF508595 | AF508704 | AF508726 |
| A/quail/Hongkong/G1/1997(H9N2) | AF156435 | AF156421 | AF156449 | AF156378 | AF156407 | AF156396 | AF156463 | AF156477 |
| A/quail/Hong kong/NT28/1999(H9N2) | AF222629 | AF222639 | AF222649 | AF186267 | AF186270 | AF222659 | AF222669 | AF222679 |
| A/quail/Hongkong/A17/1999(H9N2) | AF222622 | AF222632 | AF222642 | AF222606 | AF222614 | AF222652 | AF222662 | AF222672 |
| A/quail/Dubai/301/2000(H9N2) | EF063552 | EF063531 | EF063545 | EF063510 | EF063524 | EF063517 | EF063503 | EF063538 |
| A/quail/Dubai/302/2000(H9N2) | EF063553 | EF063532 | EF063546 | EF063511 | EF063525 | EF063518 | EF063504 | EF063539 |
| A/quail/Dubai/303/2000(H9N2) | EF063554 | EF063533 | EF063547 | EF063512 | EF063526 | EF063519 | EF063505 | EF063540 |
| A/quail/Nanchang/2-0460/2000(H9N2) | CY005506 | CY005505 | CY005504 | CY006018 | CY005503 | CY005502 | CY005501 | CY006019 |
| A/quail/Shantou/1310/2000(H9N2) | EF154836 | EF155347 | EF155274 | EF154909 | EF155128 | EF155055 | EF154982 | EF155201 |
| A/quail/Shantou/1318/2000(H9N2) | EF154837 | EF155348 | EF155275 | EF154910 | EF155129 | EF155056 | EF154983 | EF155202 |
| A/quail/Shantou/1820/2000(H9N2) | EF154838 | EF155349 | EF155276 | EF154911 | EF155130 | EF155057 | EF154984 | EF155203 |
| A/quail/Shantou/2061/2000(H9N2) | EF154839 | EF155350 | EF155277 | EF154912 | EF155131 | EF155058 | EF154985 | EF155204 |
| A/quail/Shantou/243/2000(H9N2) | EF154834 | EF155345 | EF155272 | EF154907 | EF155126 | EF155053 | EF154980 | EF155199 |
| A/quail/Shantou/2816/2000(H9N2) | EF154840 | EF155351 | EF155278 | EF154913 | EF155132 | EF155059 | EF154986 | EF155205 |
| A/quail/Shantou/782/2000(H9N2) | EF154835 | EF155346 | EF155273 | EF154908 | EF155127 | EF155054 | EF154981 | EF155200 |
| A/quail/Shantou/1158/2001(H9N2) | EF154843 | EF155354 | EF155281 | EF154916 | EF155135 | EF155062 | EF154989 | EF155208 |
| A/quail/Shantou/1235/2001(H9N2) | EF154844 | EF155355 | EF155282 | EF154917 | EF155136 | EF155063 | EF154990 | EF155209 |
| A/quail/Shantou/1242/2001(H9N2) | EF154845 | EF155356 | EF155283 | EF154918 | EF155137 | EF155064 | EF154991 | [EF155210](http://www.ncbi.nlm.nih.gov/entrez/viewer.fcgi?val=EF155210) |
| A/quail/Shantou/1425/2001(H9N2) | EF154846 | EF155357 | EF155284 | EF154919 | EF155138 | EF155065 | EF154992 | EF155211 |
| A/quail/Shantou/1461/2001(H9N2) | EF154847 | EF155358 | EF155285 | EF154920 | EF155139 | EF155066 | [EF154993](http://www.ncbi.nlm.nih.gov/entrez/viewer.fcgi?val=EF154993) | EF155212 |
| A/quail/Shantou/1555/2001(H9N2) | EF154848 | EF155359 | EF155286 | EF154921 | EF155140 | EF155067 | EF154994 | [EF155213](http://www.ncbi.nlm.nih.gov/entrez/viewer.fcgi?val=EF155213) |
| A/quail/Shantou/1912/2001(H9N2) | EF154849 | EF155360 | EF155287 | EF154922 | [EF155141](http://www.ncbi.nlm.nih.gov/entrez/viewer.fcgi?val=EF155141) | EF155068 | [EF154995](http://www.ncbi.nlm.nih.gov/entrez/viewer.fcgi?val=EF154995) | [EF155214](http://www.ncbi.nlm.nih.gov/entrez/viewer.fcgi?val=EF155214) |
| A/quail/Shantou/2111/2001(H9N2) | EF154850 | EF155361 | EF155288 | EF154923 | EF155142 | EF155069 | EF154996 | EF155215 |
| A/quail/Shantou/222/2001(H9N2) | EF154841 | EF155352 | EF155279 | EF154914 | EF155133 | EF155060 | [EF154987](http://www.ncbi.nlm.nih.gov/entrez/viewer.fcgi?val=EF154987) | EF155206 |
| A/quail/Shantou/2462/2001(H9N2) | EF154851 | EF155362 | [EF155289](http://www.ncbi.nlm.nih.gov/entrez/viewer.fcgi?val=EF155289) | EF154924 | EF155143 | EF155070 | EF154997 | EF155216 |
| A/quail/Shantou/4179/2001(H9N2) | EF154852 | EF155363 | EF155290 | EF154925 | EF155144 | EF155071 | EF154998 | EF155217 |
| A/quail/Shantou/4641/2001(H9N2) | EF154853 | EF155364 | EF155291 | EF154926 | EF155145 | EF155072 | EF154999 | EF155218 |
| A/quail/Shantou/4762/2001(H9N2) | EF154854 | EF155365 | EF155292 | EF154927 | EF155146 | EF155073 | EF155000 | EF155219 |
| A/quail/Shantou/5663/2001(H9N2) | EF154855 | EF155366 | EF155293 | EF154928 | EF155147 | EF155074 | EF155001 | EF155220 |
| A/quail/Shantou/5675/2001(H9N2) | EF154856 | EF155367 | [EF155294](http://www.ncbi.nlm.nih.gov/entrez/viewer.fcgi?val=EF155294) | EF154929 | EF155148 | EF155075 | EF155002 | [EF155221](http://www.ncbi.nlm.nih.gov/entrez/viewer.fcgi?val=EF155221) |
| A/quail/Shantou/850/2001(H9N2) | EF154842 | EF155353 | EF155280 | EF154915 | EF155134 | EF155061 | EF154988 | EF155207 |
| A/quail/Shantou/1038/2002(H9N2) | EF154862 | EF155373 | EF155300 | EF154935 | EF155154 | EF155081 | [EF155008](http://www.ncbi.nlm.nih.gov/entrez/viewer.fcgi?val=EF155008) | EF155227 |
| A/quail/Shantou/1551/2002(H9N2) | EF154863 | EF155374 | EF155301 | EF154936 | EF155155 | EF155082 | EF155009 | EF155228 |
| A/quail/Shantou/365/2002(H9N2) | EF154858 | EF155369 | EF155296 | EF154931 | EF155150 | EF155077 | EF155004 | EF155223 |
| A/quail/Shantou/384/2002(H9N2) | EF154859 | EF155370 | EF155297 | EF154932 | EF155151 | EF155078 | [EF155005](http://www.ncbi.nlm.nih.gov/entrez/viewer.fcgi?val=EF155005) | EF155224 |
| A/quail/Shantou/3851/2002(H9N2) | EF154864 | EF155375 | EF155302 | EF154937 | EF155156 | EF155083 | EF155010 | EF155229 |
| A/quail/Shantou/396/2002(H9N2) | EF154860 | EF155371 | EF155298 | EF154933 | [EF155152](http://www.ncbi.nlm.nih.gov/entrez/viewer.fcgi?val=EF155152) | EF155079 | [EF155006](http://www.ncbi.nlm.nih.gov/entrez/viewer.fcgi?val=EF155006) | EF155225 |
| A/quail/Shantou/4203/2002(H9N2) | EF154865 | EF155376 | EF155303 | EF154938 | EF155157 | EF155084 | EF155011 | EF155230 |
| A/quail/Shantou/4700/2002(H9N2) | EF154866 | EF155377 | EF155304 | EF154939 | EF155158 | EF155085 | EF155012 | EF155231 |
| A/quail/Shantou/69/2002(H9N2) | EF154857 | EF155368 | EF155295 | EF154930 | EF155149 | EF155076 | EF155003 | EF155222 |
| A/quail/Shantou/786/2002(H9N2) | EF154861 | EF155372 | EF155299 | EF154934 | EF155153 | EF155080 | EF155007 | EF155226 |
| A/quail/Shantou/1140/2003(H9N2) | EF154870 | EF155381 | EF155308 | EF154943 | EF155162 | EF155089 | EF155016 | EF155235 |
| A/quail/Shantou/149/2003(H9N2) | EF154867 | EF155378 | EF155305 | EF154940 | EF155159 | EF155086 | EF155013 | EF155232 |
| A/quail/Shantou/1780/2003(H9N2) | EF154871 | EF155382 | EF155309 | EF154944 | EF155163 | EF155090 | EF155017 | EF155236 |
| A/quail/Shantou/1978/2003(H9N2) | EF154872 | EF155383 | EF155310 | EF154945 | EF155164 | EF155091 | EF155018 | EF155237 |
| A/quail/Shantou/2608/2003(H9N2) | EF154873 | EF155384 | EF155311 | EF154946 | EF155165 | EF155092 | EF155019 | EF155238 |
| A/quail/Shantou/2615/2003(H9N2) | EF154874 | EF155385 | EF155312 | EF154947 | EF155166 | EF155093 | EF155020 | EF155239 |
| A/quail/Shantou/3008/2003(H9N2) | EF154875 | EF155386 | EF155313 | EF154948 | EF155167 | EF155094 | EF155021 | EF155240 |
| A/quail/Shantou/308/2003(H9N2) | EF154868 | EF155379 | EF155306 | EF154941 | [EF155160](http://www.ncbi.nlm.nih.gov/entrez/viewer.fcgi?val=EF155160) | EF155087 | EF155014 | EF155233 |
| A/quail/Shantou/335/2003(H9N2) | EF154869 | EF155380 | EF155307 | EF154942 | EF155161 | EF155088 | EF155015 | EF155234 |
| A/quail/Shantou/3502/2003(H9N2) | EF154877 | EF155388 | EF155315 | EF154950 | EF155169 | EF155096 | EF155023 | EF155242 |
| A/quail/Shantou/3700/2003(H9N2) | EF154878 | EF155389 | EF155316 | EF154951 | EF155170 | EF155097 | EF155024 | EF155243 |
| A/quail/Shantou/3768/2003(H9N2) | EF154879 | EF155390 | EF155317 | EF154952 | EF155171 | [EF155098](http://www.ncbi.nlm.nih.gov/entrez/viewer.fcgi?val=EF155098) | EF155025 | EF155244 |
| A/quail/Shantou/3856/2003(H9N2) | EF154880 | EF155391 | [EF155318](http://www.ncbi.nlm.nih.gov/entrez/viewer.fcgi?val=EF155318) | EF154953 | EF155172 | EF155099 | [EF155026](http://www.ncbi.nlm.nih.gov/entrez/viewer.fcgi?val=EF155026) | EF155245 |
| A/quail/Shantou/4038/2003(H9N2) | EF154881 | EF155392 | EF155319 | EF154954 | EF155173 | EF155100 | [EF155027](http://www.ncbi.nlm.nih.gov/entrez/viewer.fcgi?val=EF155027) | EF155246 |
| A/quail/Shantou/4044/2003(H9N2) | EF154882 | EF155393 | EF155320 | EF154955 | EF155174 | EF155101 | [EF155028](http://www.ncbi.nlm.nih.gov/entrez/viewer.fcgi?val=EF155028) | EF155247 |
| A/quail/Shantou/1475/2004(H9N2) | EF154886 | EF155397 | EF155324 | EF154959 | [EF155178](http://www.ncbi.nlm.nih.gov/entrez/viewer.fcgi?val=EF155178) | EF155105 | EF155032 | EF155251 |
| A/quail/Shantou/1865/2004(H9N2) | EF154887 | EF155398 | EF155325 | EF154960 | EF155179 | EF155106 | [EF155033](http://www.ncbi.nlm.nih.gov/entrez/viewer.fcgi?val=EF155033) | EF155252 |
| A/quail/Shantou/1883/2004(H9N2) | EF154888 | EF155399 | EF155326 | EF154961 | EF155180 | EF155107 | EF155034 | EF155253 |
| A/quail/Shantou/3060/2004(H9N2) | EF154889 | EF155400 | EF155327 | EF154962 | [EF155181](http://www.ncbi.nlm.nih.gov/entrez/viewer.fcgi?val=EF155181) | EF155108 | EF155035 | EF155254 |
| A/quail/Shantou/403/2004(H9N2) | EF154885 | EF155396 | EF155323 | EF154958 | EF155177 | EF155104 | [EF155031](http://www.ncbi.nlm.nih.gov/entrez/viewer.fcgi?val=EF155031) | EF155250 |
| A/quail/Shantou/6046/2004(H9N2) | EF154890 | EF155401 | EF155328 | EF154963 | EF155182 | EF155109 | EF155036 | EF155255 |
| A/quail/Shantou/6648/2004(H9N2) | EF154891 | EF155402 | EF155329 | EF154964 | EF155183 | EF155110 | EF155037 | EF155256 |
| A/quail/Shantou/6794/2004(H9N2) | EF154892 | EF155403 | EF155330 | EF154965 | EF155184 | EF155111 | EF155038 | EF155257 |
| A/quail/Shantou/7397/2004(H9N2) | EF154893 | EF155404 | EF155331 | EF154966 | EF155185 | EF155112 | EF155039 | EF155258 |
| A/quail/Shantou/7731/2004(H9N2) | EF154894 | EF155405 | EF155332 | EF154967 | [EF155186](http://www.ncbi.nlm.nih.gov/entrez/viewer.fcgi?val=EF155186) | [EF155113](http://www.ncbi.nlm.nih.gov/entrez/viewer.fcgi?val=EF155113) | EF155040 | EF155259 |
| A/quail/Shantou/11195/2005(H9N2) | EF154901 | EF155412 | EF155339 | EF154974 | EF155193 | EF155120 | EF155047 | EF155266 |
| A/quail/Shantou/13425/2005(H9N2) | EF154902 | EF155413 | EF155340 | EF154975 | [EF155138](http://www.ncbi.nlm.nih.gov/entrez/viewer.fcgi?val=EF155138) | EF155121 | [EF155048](http://www.ncbi.nlm.nih.gov/entrez/viewer.fcgi?val=EF155048) | EF155267 |
| A/quail/Shantou/15892/2005(H9N2) | EF154903 | EF155414 | EF155341 | EF154976 | EF155195 | EF155122 | EF155049 | EF155268 |
| A/quail/Shantou/19506/2005(H9N2) | EF154904 | EF155415 | EF155342 | EF154977 | EF155196 | EF155123 | [EF155050](http://www.ncbi.nlm.nih.gov/entrez/viewer.fcgi?val=EF155050) | [EF155269](http://www.ncbi.nlm.nih.gov/entrez/viewer.fcgi?val=EF155269) |
| A/quail/Shantou/20787/2005(H9N2) | EF154905 | EF155416 | EF155343 | EF154978 | [EF155197](http://www.ncbi.nlm.nih.gov/entrez/viewer.fcgi?val=EF155197) | EF155124 | EF155051 | EF155270 |
| A/quail/Shantou/21605/2005(H9N2) | EF154906 | EF155417 | EF155344 | EF154979 | [EF155198](http://www.ncbi.nlm.nih.gov/entrez/viewer.fcgi?val=EF155198) | EF155125 | EF155052 | EF155271 |
| A/quail/Shantou/2200/2005(H9N2) | EF154897 | EF155408 | EF155335 | EF154970 | EF155189 | EF155116 | EF155043 | EF155262 |
| A/quail/Shantou/299/2005(H9N2) | EF154895 | EF155406 | EF155333 | EF154968 | EF155187 | EF155114 | EF155041 | EF155260 |
| A/quail/Shantou/3143/2005(H9N2) | EF154898 | EF155409 | EF155336 | EF154971 | EF155190 | EF155117 | [EF155044](http://www.ncbi.nlm.nih.gov/entrez/viewer.fcgi?val=EF155044) | EF155263 |
| A/quail/Shantou/493/2005(H9N2) | EF154896 | EF155407 | [EF155334](http://www.ncbi.nlm.nih.gov/entrez/viewer.fcgi?val=EF155334) | EF154969 | [EF155188](http://www.ncbi.nlm.nih.gov/entrez/viewer.fcgi?val=EF155188) | [EF155115](http://www.ncbi.nlm.nih.gov/entrez/viewer.fcgi?val=EF155115) | EF155042 | EF155261 |
| A/quail/Shantou/5011/2005(H9N2) | EF154899 | [EF155410](http://www.ncbi.nlm.nih.gov/entrez/viewer.fcgi?val=EF155410) | EF155337 | EF154972 | EF155191 | EF155118 | EF155045 | EF155264 |
| A/quail/Shantou/8993/2005(H9N2) | EF154900 | EF155411 | [EF155338](http://www.ncbi.nlm.nih.gov/entrez/viewer.fcgi?val=EF155338) | EF154973 | EF155192 | EF155119 | EF155046 | [EF155265](http://www.ncbi.nlm.nih.gov/entrez/viewer.fcgi?val=EF155265) |
| A/quail/Guangxi/B1/2006(H9N2) | EU086299 | EU086297 | EU086294 | EU086283 | EU086289 | EU086287 | EU086285 | [EU086292](http://www.ncbi.nlm.nih.gov/entrez/viewer.fcgi?val=EU086292) |
| A/sanderling/Delaware/482/2006(H9N2) | CY041441 | CY041440 | CY041439 | CY041434 | CY041437 | CY041436 | CY041435 | CY041438 |
| A/SCK/HK/WF285/2003(H9N2) | DQ226177 | DQ226166 | DQ226155 | DQ226111 | DQ226144 | DQ226133 | DQ226100 | DQ226122 |
| A/SCK/HK/YU663/2003(H9N2) | DQ226180 | DQ226169 | DQ226158 | DQ226114 | DQ226147 | DQ226136 | DQ226103 | DQ226125 |
| A/Shorebird/Delaware/9/1996(H9N2) | AF156441 | AF156427 | AF156455 | AF156386 | AF156413 | / | AF156469 | AF156483 |
| A/Shorebird/DE/66/2003(H9N2) | CY005165 | CY005164 | / | / | CY005162 | / | CY005161 | CY005163 |
| A/shorebird/Delaware/249/2006(H9N2) | CY043919 | [CY043918](http://www.ncbi.nlm.nih.gov/entrez/viewer.fcgi?val=CY043918) | CY043917 | CY043912 | CY043915 | CY043914 | CY043913 | CY043916 |
| A/silky chicken/Hong Kong/SF44/1999(H9N2) | AF222631 | AF222641 | AF222651 | AF222613 | AF222621 | AF222661 | [AF222671](http://www.ncbi.nlm.nih.gov/entrez/viewer.fcgi?val=AF222671) | AF222681 |
| A/silky chicken/Shantou/1818/2000(H9N2) | CY024613 | CY024614 | CY024615 | CY024616 | CY024617 | CY024618 | CY024619 | [CY024620](http://www.ncbi.nlm.nih.gov/entrez/viewer.fcgi?val=CY024620) |
| A/silky chicken/Shantou/2619/2001(H9N2) | CY024677 | CY024678 | CY024679 | CY024680 | CY024681 | CY024682 | CY024683 | CY024684 |
| A/silky chicken/Korea/S3/2003(H9N2) | AY862710 | AY862694 | [AY862678](http://www.ncbi.nlm.nih.gov/entrez/viewer.fcgi?val=AY862678) | AY862598 | AY862646 | AY862630 | AY862614 | AY862662 |
| A/silky chicken/4600/2003(H9N2) | CY023245 | CY023246 | CY023247 | CY023248 | CY023249 | [CY023250](http://www.ncbi.nlm.nih.gov/entrez/viewer.fcgi?val=CY023250) | CY023251 | CY023252 |
| A/silky chicken/Shantou/1826/2004(H9N2) | CY023301 | CY023302 | CY023303 | CY023304 | CY023305 | CY023306 | CY023307 | CY023308 |
| A/silky chicken/Shantou/473/2004(H9N2) | CY023277 | CY023278 | CY023279 | CY023280 | CY023281 | CY023282 | CY023283 | CY023284 |
| A/silky chicken/Shantou/6020/2004(H9N2) | CY023365 | CY023366 | [CY023367](http://www.ncbi.nlm.nih.gov/entrez/viewer.fcgi?val=CY023367) | CY023368 | CY023369 | CY023370 | [CY023371](http://www.ncbi.nlm.nih.gov/entrez/viewer.fcgi?val=CY023371) | CY023372 |
| A/silky chicken/Shantou/7790/2004(H9N2) | CY023397 | [CY023398](http://www.ncbi.nlm.nih.gov/entrez/viewer.fcgi?val=CY023397) | [CY023399](http://www.ncbi.nlm.nih.gov/entrez/viewer.fcgi?val=CY023397) | [CY023400](http://www.ncbi.nlm.nih.gov/entrez/viewer.fcgi?val=CY023397) | [CY023401](http://www.ncbi.nlm.nih.gov/entrez/viewer.fcgi?val=CY023397) | [CY023402](http://www.ncbi.nlm.nih.gov/entrez/viewer.fcgi?val=CY023397) | [CY023403](http://www.ncbi.nlm.nih.gov/entrez/viewer.fcgi?val=CY023397) | [CY023404](http://www.ncbi.nlm.nih.gov/entrez/viewer.fcgi?val=CY023397) |
| A/silky chicken/Shantou/999/2004(H9N2) | CY023285 | CY023286 | CY023287 | CY023288 | CY023289 | CY023290 | CY023291 | CY023292 |
| A/silky chicken/Shantou/1169/2005(H9N2) | CY023429 | CY023430 | CY023431 | CY023432 | CY023433 | CY023434 | CY023435 | CY023436 |
| A/silky chicken/Shantou/2131/2005(H9N2) | CY023437 | CY023438 | CY023439 | CY023440 | CY023441 | CY023442 | CY023443 | CY023444 |
| A/silky chicken/Shantou/3581/2005(H9N2) | CY023453 | CY023454 | CY023455 | CY023456 | CY023457 | CY023458 | CY023459 | CY023460 |
| A/silky chicken/Shantou/459/2005(H9N2) | CY023421 | CY023422 | CY023423 | CY023424 | CY023425 | CY023426 | CY023427 | [CY023428](http://www.ncbi.nlm.nih.gov/entrez/viewer.fcgi?val=CY023428) |
| A/turkey/California/189/1966(H9N2) | AF156443 | AF156429 | AF156457 | AF156390 | AF156415 | AF156401 | AF156471 | DQ787799 |
| A/turkey/WI/1966(H9N2) | EU182278 | EU182279 | EU182280 | CY014663 | EU182281 | EU182282 | EU182283 | EU182284 |
| A/turkey/Wisconsin/1/1966(H9N2) | CY014670 | CY014669 | CY014668 | GQ247858 | CY014666 | CY014665 | CY014664 | CY014667 |
| A/turkey/Wisconsin/1966(H9N2) | DQ067437 | DQ067443 | DQ067442 | DQ067444 | DQ067440 | DQ067439 | DQ067438 | DQ067441 |
| A/turkey/TX/4-1-81/1981(H9N2) | EU982323 | EU982322 | EU982321 | EU982316 | EU982319 | EU982318 | EU982317 | EU982320 |
| A/turkey/TX/10-49-89/1989(H9N2) | EU982315 | EU982314 | [EU982313](http://www.ncbi.nlm.nih.gov/entrez/viewer.fcgi?val=EU982313) | EU982308 | EU982311 | EU982310 | EU982309 | EU982312 |
| A/turkey/Israel/90710/2000(H9N2) | EF492400 | EF492371 | EF492342 | AY738451 | EF492302 | EF492273 | EF492244 | DQ683025 |
| A/turkey/Israel/810/2001(H9N2) | EF492412 | EF492383 | EF492354 | EF492233 | EF492314 | EF492285 | [EF492270](http://www.ncbi.nlm.nih.gov/entrez/viewer.fcgi?val=EF492270) | DQ683033 |
| A/turkey/Israel/1013/2002(H9N2) | EF492417 | [EF492388](http://www.ncbi.nlm.nih.gov/entrez/viewer.fcgi?val=EF492388) | EF492359 | EF492237 | EF492319 | EF492290 | EF492257 | DQ683036 |
| A/turkey/Israel/619/2002(H9N2) | EF492408 | EF492379 | EF492350 | EF492229 | EF492310 | EF492281 | [EF492268](http://www.ncbi.nlm.nih.gov/entrez/viewer.fcgi?val=EF492268) | DQ683031 |
| A/turkey/Israel/965/2002(H9N2) | EF492416 | [EF492387](http://www.ncbi.nlm.nih.gov/entrez/viewer.fcgi?val=EF492387) | EF492358 | / | EF492318 | EF492289 | EF492271 | DQ683035 |
| A/turkey/Israel/1209/2003(H9N2) | EF492418 | EF492389 | EF492360 | EF492238 | EF492320 | EF492291 | EF492258 | DQ683037 |
| A/turkey/Israel/1562/2004(H9N2) | EF492423 | EF492394 | EF492365 | / | EF492325 | EF492296 | EF492263 | DQ683042 |
| A/turkey/Israel/1567/2004(H9N2) | EF492424 | EF492395 | EF492366 | EF492241 | EF492326 | EF492297 | [EF492264](http://www.ncbi.nlm.nih.gov/entrez/viewer.fcgi?val=EF492264) | DQ683043 |
| A/turkey/Israel/425/2005(H9N2) | EF492405 | EF492376 | EF492347 | EF492226 | EF492307 | EF492278 | EF492249 | EF492335 |
| A/turkey/Israel/747/2005(H9N2) | EF492409 | EF492380 | EF492351 | EF492230 | EF492311 | EF492282 | EF492252 | EF492338 |
| A/turkey/Israel/884/2005(H9N2) | EF492414 | EF492385 | EF492356 | EF492235 | EF492316 | EF492287 | [EF492255](http://www.ncbi.nlm.nih.gov/entrez/viewer.fcgi?val=EF492255) | EF492341 |
| A/turkey/Israel/89/2005(H9N2) | EF492402 | EF492373 | EF492344 | EF492223 | EF492304 | EF492275 | EF492246 | DQ683026 |
| A/turkey/Italy/1325/2005(H5N2) | CY022636 | [CY022635](http://www.ncbi.nlm.nih.gov/entrez/viewer.fcgi?val=CY022635) | CY022634 | CY022629 | CY022632 | CY022631 | CY022630 | CY022633 |
| A/turkey/Israel/1608/2006(H9N2) | FJ464713 | FJ464696 | FJ464679 | FJ464730 | FJ464646 | FJ464629 | FJ464612 | [FJ464663](http://www.ncbi.nlm.nih.gov/entrez/viewer.fcgi?val=FJ464663) |
| A/turkey/Israel/900/2007(H9N2) | FJ464708 | FJ464691 | FJ464674 | FJ464725 | FJ464641 | FJ464624 | FJ464607 | FJ464658 |
| A/turkey/Israel/689/2008(H9N2) | GQ140270 | GQ148869 | GQ140284 | GQ120554 | GQ148841 | GQ120541 | GQ148827 | GQ148855 |
| A/Wild Duck/Nanchang/2-0480/2000(H9N2) | CY005518 | AY180897 | CY005516 | CY006021 | / | / | [CY005513](http://www.ncbi.nlm.nih.gov/entrez/viewer.fcgi?val=CY005513) | [CY006022](http://www.ncbi.nlm.nih.gov/entrez/viewer.fcgi?val=CY006022) |
| A/Wild Duck/Shantou/4808/2001(H9N2) | AF523464 | AF523431 | AF523454 | AF523381 | AF523412 | AF523394 | [AF523484](http://www.ncbi.nlm.nih.gov/entrez/viewer.fcgi?val=AF523484) | AF523514 |
| A/Hong Kong/483/97(H5N1) | AF258839 | [AF258820](http://www.ncbi.nlm.nih.gov/entrez/viewer.fcgi?val=AF258820) | [AF257195](http://www.ncbi.nlm.nih.gov/entrez/viewer.fcgi?val=AF257195) | AF046097 | AF255746 | AF102668 | AF255367 | AF256180 |
| A/Hong Kong/486/97(H5N1) | AF115291 | [AF115293](http://www.ncbi.nlm.nih.gov/entrez/viewer.fcgi?val=AF115293) | [AF257196](http://www.ncbi.nlm.nih.gov/entrez/viewer.fcgi?val=AF257196) | AF084281 | AF255747 | AF084275 | AF255368 | AF256181 |
| A/HongKong/156/97(H5N1) | AF036363 | [AF036362](http://www.ncbi.nlm.nih.gov/entrez/viewer.fcgi?val=AF036362) | AJ289874 | AF046088 | AF036359 | AF036357 | AF036358 | [AF036360](http://www.ncbi.nlm.nih.gov/entrez/viewer.fcgi?val=AF036360) |
| A/Guangzhou/333/1999(H9N2) | AY043030 | AY043029 | AY043028 | AY043019 | AY043026 | AY043024 | AY043025 | AY043027 |
| A/Hongkong/1073/1999(H9N2) | AJ404630 | [AF258816](http://www.ncbi.nlm.nih.gov/entrez/viewer.fcgi?val=AF258816) | [AJ404637](http://www.ncbi.nlm.nih.gov/entrez/viewer.fcgi?val=AJ404637) | AJ404626 | AJ289871 | [AJ404629](http://www.ncbi.nlm.nih.gov/entrez/viewer.fcgi?val=AJ404629) | AJ278647 | AJ278649 |
| A/Hongkong/1074/1999(H9N2) | AF258836 | [AF258817](http://www.ncbi.nlm.nih.gov/entrez/viewer.fcgi?val=AF258817) | [AF257192](http://www.ncbi.nlm.nih.gov/entrez/viewer.fcgi?val=AF257192) | AJ404627 | AF255743 | AJ404628 | AF255364 | AF256177 |
| A/Korea/KBNP-0028/2000(H9N2) | EF620897 | EF620898 | EF620899 | EF620900 | EF620901 | EF620902 | EF620903 | EF620904 |
| A/HK/2108/2003(H9N2) | DQ226172 | DQ226161 | DQ226150 | DQ226106 | DQ226139 | DQ226128 | DQ226095 | DQ226117 |
| A/Netherlands/219/03(H7N7) | AY342413 | [AY340083](http://www.ncbi.nlm.nih.gov/entrez/viewer.fcgi?val=AY340083) | [AY342418](http://www.ncbi.nlm.nih.gov/entrez/viewer.fcgi?val=AY342418) | AY338459 | AY342425 | AY340079 | AY340089 | AY342422 |
| A/swine/Hong kong/9/1998(H9N2) | AF222816 | AF222818 | AF222820 | AF222810 | AF222814 | AF222812 | AF222822 | AF222824 |
| A/swine/Shandong/na/2003(H9N2) | DQ997418 | DQ997417 | DQ997424 | DQ997419 | DQ997422 | DQ997421 | DQ997420 | DQ997423 |
| A/swine/Shandong/nb/2003(H9N2) | DQ997427 | DQ997426 | DQ997432 | DQ997428 | DQ997430 | DQ997429 | DQ997425 | DQ997431 |
| A/swine/Shandong/W4/2003(H9N2) | EU516309 | EU516310 | EU516311 | EU516304 | EU516308 | EU516305 | EU516306 | EU516307 |
| A/swine/Guangdong/WXI/2004(H9N2) | EU516317 | EU516318 | EU516319 | EU516312 | EU516316 | EU516313 | EU516314 | EU516315 |
| A/swine/Jiangxi/1/2004(H9N2) | EU502898 | EU502899 | EU502900 | EU502893 | EU502897 | EU502894 | EU502895 | EU502896 |
| A/swine/Jiangxi/wx2/2004(H9N2) | EU502906 | EU502907 | [EU502908](http://www.ncbi.nlm.nih.gov/entrez/viewer.fcgi?val=EU502908) | EU502901 | EU502905 | EU502902 | EU502903 | EU502904 |
| A/swine/Korea/S190/2004(H9N2) | AY790304 | AY790303 | AY790302 | AY790297 | AY790300 | AY790299 | AY790298 | AY790301 |
| A/swine/Korea/S452/2004(H9N2) | AY790311 | AY790312 | AY790310 | AY790305 | AY790308 | AY790307 | AY790306 | AY790309 |
| A/swine/Guangxi/58/2005(H9N2) | EF612749 | EF612748 | EF612747 | EF612742 | EF612745 | EF612744 | EF612743 | EF612746 |
| A/swine/Guangxi/FS2/2005(H9N2) | EU086316 | EU086314 | EU086313 | EU086302 | EU086309 | EU086306 | EU086304 | [EU086310](http://www.ncbi.nlm.nih.gov/entrez/viewer.fcgi?val=EU086310) |
| A/swine/Guangxi/S11/2005(H9N2) | EU086332 | EU086330 | EU086329 | EU086319 | EU086325 | EU086323 | EU086320 | [EU086327](http://www.ncbi.nlm.nih.gov/entrez/viewer.fcgi?val=EU086327) |
| A/swine/Guangxi/S15/2005(H9N2) | EU086333 | EU086331 | EU086328 | EU086318 | EU086324 | EU086322 | [EU086321](http://www.ncbi.nlm.nih.gov/entrez/viewer.fcgi?val=EU086321) | EU086326 |
| A/swine/Shandong/nc/2005(H9N2) | DQ997435 | DQ997434 | DQ997433 | DQ997437 | DQ997439 | DQ997438 | DQ997436 | DQ997440 |
